# Supplementary material for: Dynamic Gene Regulatory Networks Drive Hematopoietic Specification and Differentiation
Source: Dev Cell. 2016 Mar 7;36(5):572–87. doi: 10.1016/j.devcel.2016.01.024 (PMC4780867; doi:10.1016/j.devcel.2016.01.024)
Supplement: Document S1. Supplemental Experimental Procedures, Figures S1–S7, and Table S6 [file mmc1.pdf]

Developmental Cell, Volume *36*

Supplemental Information

Dynamic Gene Regulatory Networks Drive Hematopoietic Specification and Differentiation

Debbie K. Goode, Nadine Obier, M.S. Vijayabaskar, Michael Lie-A-Ling, Andrew J. Lilly, Rebecca Hannah, Monika Lichtinger, Kiran Batta, Magdalena Florkowska, Rahima Patel, Mairi Challinor, Kirstie Wallace, Jane Gilmour, Salam A. Assi, Pierre Cauchy, Maarten Hoogenkamp, David R. Westhead, Georges Lacaud, Valerie Kouskoff, Berthold Göttgens, and Constanze Bonifer

Goode et al., Supplemental Materials

1. SUPPLEMENTAL FIGURES, FIGURE LEGENDS AND TABLES

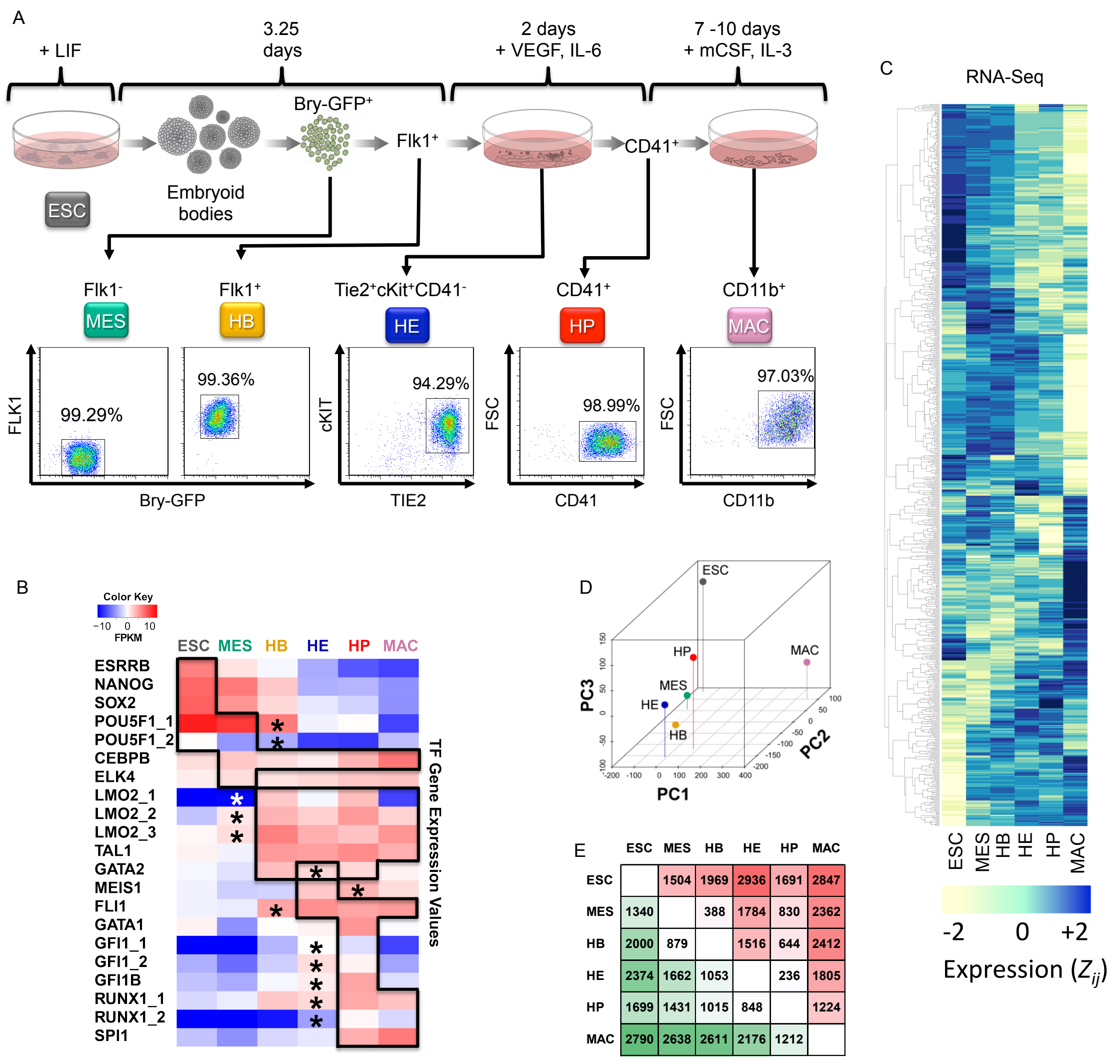

Figure S1 (related to Figure 1): (**A**) Schematic diagram of *in vitro* differentiation time course and isolation of cells at six different stages of development. ES cells are maintained in media supplemented with LIF, then allowed to differentiate into embryoid bodies. After just over three days, GFP positive cells (expressing Brachyury, Bry) are sorted into Flk1 negative (mesoderm, MES) and Flk1 positive (hemangioblast, HB) populations. Supplemented by cytokines (indicated), Flk1 positive cells are allowed to differentiate further towards hemogenic endothelium (HE), hematopoietic progenitors (HP) and finally macrophages (MAC). Each population is isolated using the sorting strategy indicated below. (**B**) Heatmap displaying of the expression of transcription factor genes

(e_{ij}, see supplemental methods) that are two fold up- or down- regulated between any two pairs of cell lines across the developmental time course and computed as detailed in the methods section. Stages selected for TF ChIP experiments that passed our quality control are outlined by the black border and those that failed are asterisked. Note that some genes generate different isoforms and are listed multiple times. (**C)** Clustering analysis of the expression of genes (Z_{ij}, see supplementary methods) that are two fold up- (yellow) or down- (blue) regulated between any two pairs of cell lines computed as given in the methods section. (**D)** Principal Component Analyses of RNA-Seq data. The cell lines are projected onto the top three principal components that contribute to almost 90% of the variance in the gene expression. The contribution of a given principal component to the variation observed in the gene expression data are: PC1=56.5%; PC2=21.5%; PC3=10.8%. **(E)** Number of differentially expressed genes (Fold change ≥ 2 and FPKM ≥ 1) with down-regulated genes indicated in green, and up-regulated genes indicated in red.

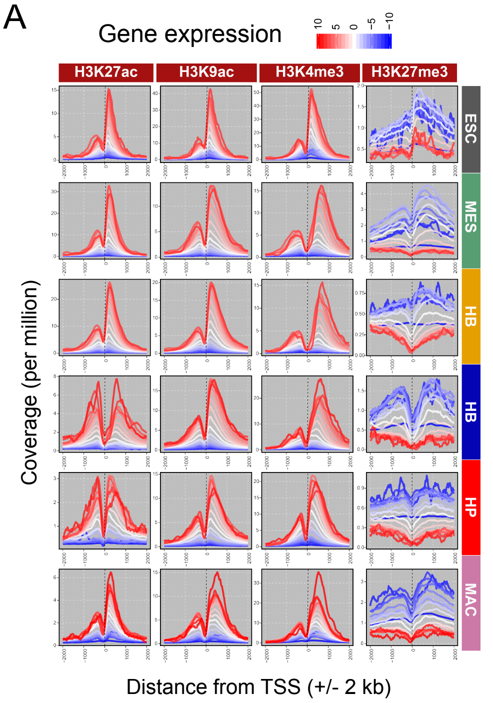

B

Chromatin State ID	DHS	H3K27AC	H3K9AC	H3K4ME3	H3K27ME3	Coarse grained state
	Emission Probabilities					
1	0.53	0.92	0.98	0.99	0.02	Active
2	0.31	0.10	0.71	0.81	0.02	Active
18	0.56	0.92	0.88	0.07	0.00	Active
19	0.33	0.47	0.13	0.04	0.01	Active
22	0.07	0.13	0.63	0.00	0.00	Active
3	0.10	0.02	0.50	0.71	0.37	Poised
6	0.38	0.31	0.42	0.67	0.83	Poised
11	0.04	0.04	0.03	0.01	0.78	Repressed
4	0.00	0.00	0.00	0.01	0.00	None
5	0.00	0.00	0.00	0.03	0.01	None
7	0.00	0.00	0.00	0.00	0.00	None
8	0.01	0.00	0.00	0.00	0.21	None
9	0.00	0.00	0.00	0.00	0.00	None
10	0.00	0.00	0.00	0.00	0.00	None
12	0.00	0.01	0.01	0.00	0.04	None
13	0.00	0.00	0.00	0.00	0.01	None
14	0.00	0.03	0.02	0.00	0.09	None
15	0.02	0.26	0.12	0.01	0.25	None
16	0.01	0.27	0.05	0.00	0.01	None
17	0.01	0.03	0.03	0.00	0.00	None
23	0.00	0.00	0.02	0.00	0.00	None
21	0.01	0.00	0.02	0.00	0.01	None
20	0.78	0.13	0.09	0.07	0.07	DHSOnly

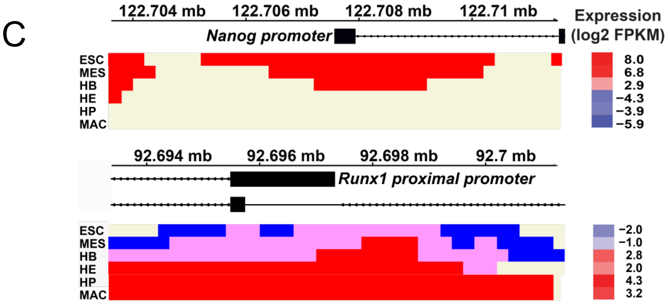

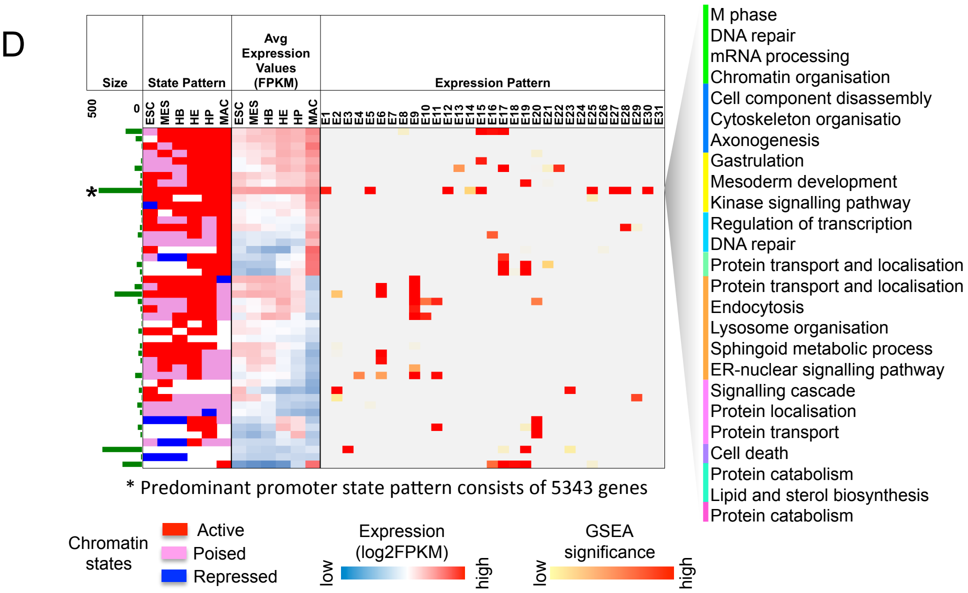

Figure S2 (related to Figure 2): **(A)** The average tag density profiles, calculated as coverage per million (cpm), of histone marks around the TSSs clustered according to the expression values of their associated genes. For each chromatin mark (column) in a given cell line (row) the tag counts (bin size of 20bp) were computed within ± 2kb of the TSSs of all coding regions and normalised as per million aligned reads. The TSSs were grouped

according to their expression values ($\log_2(FPKM)$) and for each group the tag counts were averaged for each bin and plotted. Therefore the plot shows the average tag density profile of a given histone mark around the TSS clustered according to the gene expression values. **(B)** Emission probabilities of the 23 states identified from the chromHMM (see supplementary methods section) model obtained from all four histone marks ChIP-seq and the DNaseI-seq data. The coarse-grained states used further in the analyses are given in the last column. **(C)** Example of the chromatin states at the promoters (promoter states) of *Nanog* and *Runx1* with their expression values given at the right. **(D)** Correlation between the promoter state patterns and gene expression patterns. The predominant chromatin states at the promoters were identified as described in the supplementary data analysis section. Promoters with the same state pattern (second column) were clustered together and their population sizes are given as a bar chart in the first column. The average expression values for the genes belonging to the promoters are given in the third column. The significance of overlap between a gene set belonging to a state pattern and an expression pattern (E1 to E31) is denoted as yellow to red boxes scaled according to *p*-values obtained from hypergeometric tests (Gene Set Enrichment Analyses, GSEA, see scale bar). *p*-values greater than 0.05 are given in grey. The functional descriptions given on the right relate to GO term enrichment for the 10 clusters of genes (denoted by colour coded bars, see Table S3) identified in Figure 2B. These genes are associated with the predominant promoter pattern (always active) indicated by the asterisk in the left hand bar chart.

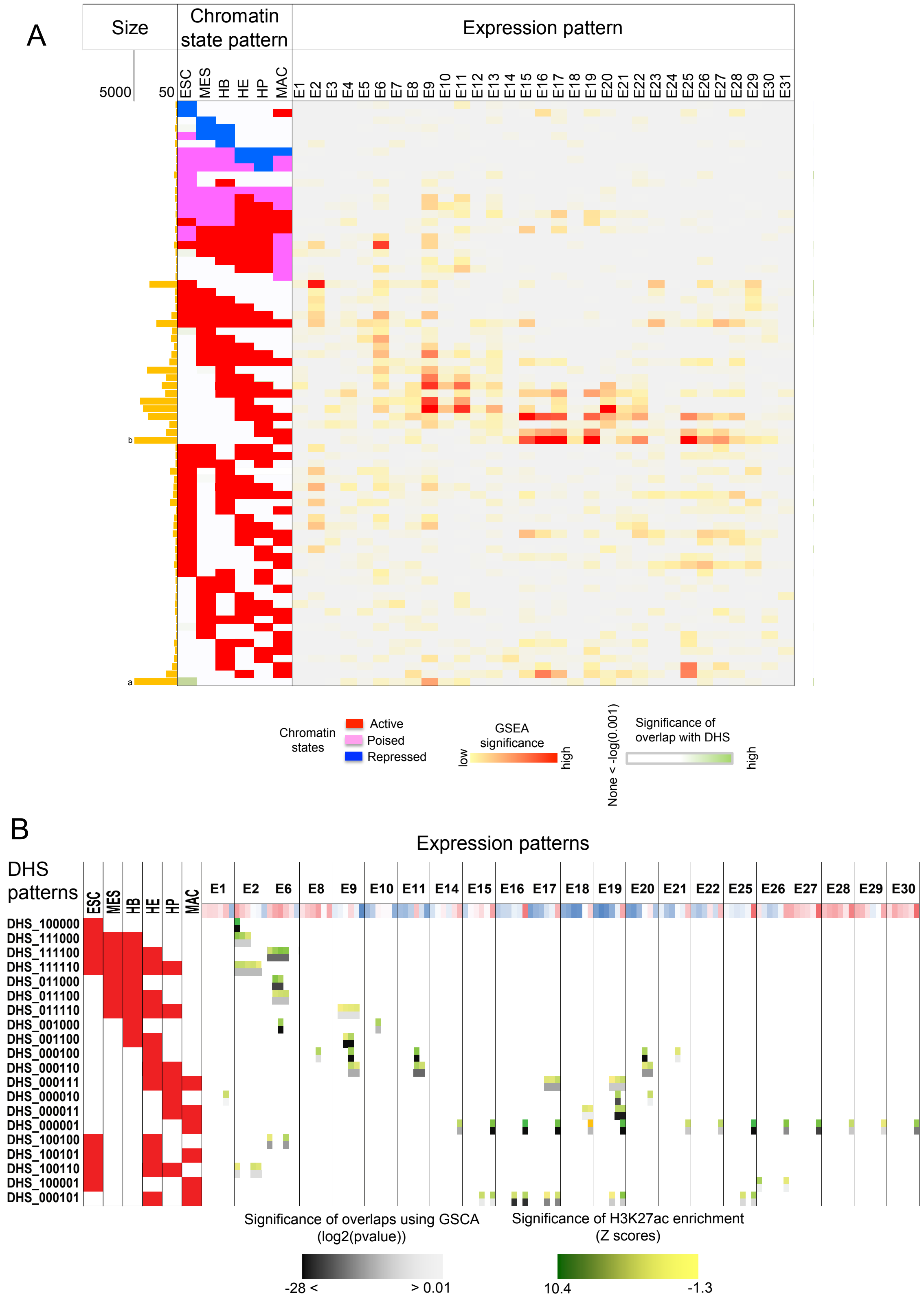

Figure S3 (related to Figure 3): (A) The predominant chromatin states for the distal DHSs were identified from the coarse-grained chromHMM model, similar to Figure S2D. The distal sites were clustered together based on matching chromatin state patterns (CSP) across all the six cell populations and only CSPs consisting of 50 or more elements were considered. In the heatmap (left panel) each row represents a CSP with the yellow bar chart to its left displaying the total number of regulatory sites (size) in each CSP. For a given set of genes associated with CSP (rows) and a specific expression pattern

(columns), the overlap significance is computed using hypergeometric testing and is adjusted for multiple testing using the Benjamini Hochberg method. The heatmap on the right panel shows the significance of overlaps (-log(p-value)) where the non-significant overlaps (p-value > 0.01) are shown in grey. a: total peaks 23114, b: total peaks 9837. Integrated into the CSP heatmap is a green heatmap showing the significance of overlap of each CSP with DHS in each cell population calculated from hypergeometric testing. A green colour observed in the CSP that is “None” indicates DHS without accompanying histone modifications. This is evident in the 23114 peaks in the last row (‘a’ in the left hand bar chart) which consists of DHSs without accompanying histone modifications. The next most prevalent CSP, b (total peaks 9837), consist of a stage specific pattern in macrophages. **(B)** Correlation of DHS-pattern frequencies (rows) with gene expression patterns (columns). The average log2(*FPKM*) for genes in a given expression pattern is plotted as the blue-red heatmap in the top row. DHS patterns are indicated at the left, using both a binary code (1=presence) and a grid depiction (red=presence) to denote presence at a specific developmental stage. GSCA was used to calculate the significance of overlaps between a given DHS pattern and an expression pattern and is plotted as a grey scale bar. Only those patterns that were significantly enriched are shown (p-value <0.01). For each DHS pattern, if the hypersensitive sites are open at a given cell stage, the significance of H3K27ac enrichment was calculated by bootstrapping and this is plotted as a yellow-green scale bar.

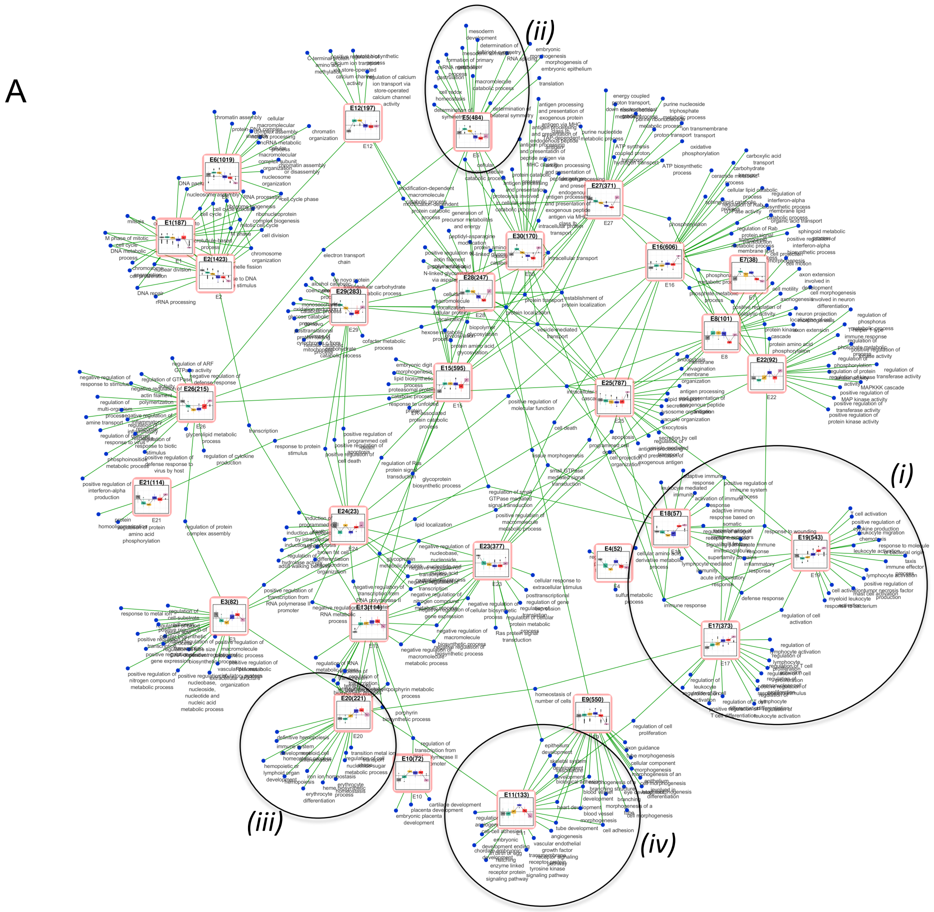

http://www.bioinformatics.leeds.ac.uk/labpages/hematopoiesis/wp-content/uploads/sites/3/2015/02/goNet_2.svg

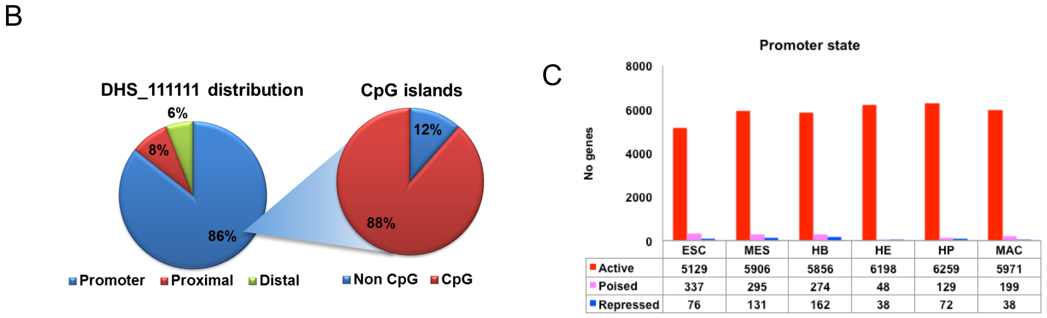

	ESC	MES	HB	HE	HP	MAC
Active	5129	5906	5856	6198	6259	5971
Poised	337	295	274	48	129	199
Repressed	76	131	162	38	72	38

Figure S4 (related to Figure 3 and 4): (A) Network of the top 20 GO terms with the best *p*-values in each of the 31 expression patterns depicted in Figure 1F. The node set comprises patterns E1 to E31 and the enriched GO terms and edges are drawn from the GO terms to the pattern in which it is over-represented. Details can be seen by zooming in or by visiting http://www.bioinformatics.leeds.ac.uk/labpages/hematopoiesis/expression-pattern-gene-ontology-network/. The layout aims at grouping the expression patterns with shared functions in the centre, with the periphery populated by expression patterns with unique functional annotations. A few important biological processes and the associated expression patterns are highlighted in circles (i) Immune response (ii) Mesoderm development (iii) Hematopoiesis and (iv) Vascular and endothelial development. The expression patterns in the centre share a large number of enriched GO terms and interestingly form a backbone of essential cellular housekeeping processes. **(B)**

Distribution of DHS_111111 relative to TSS: Promoter (±2kb), proximal (±20kb) and distal (> 20kb). The majority occur at promoters which predominantly consist of CpG islands (right hand pie chart). (**C)** The state of the promoter (active/poised/repressed) at genes associated with DHS_111111 regions.

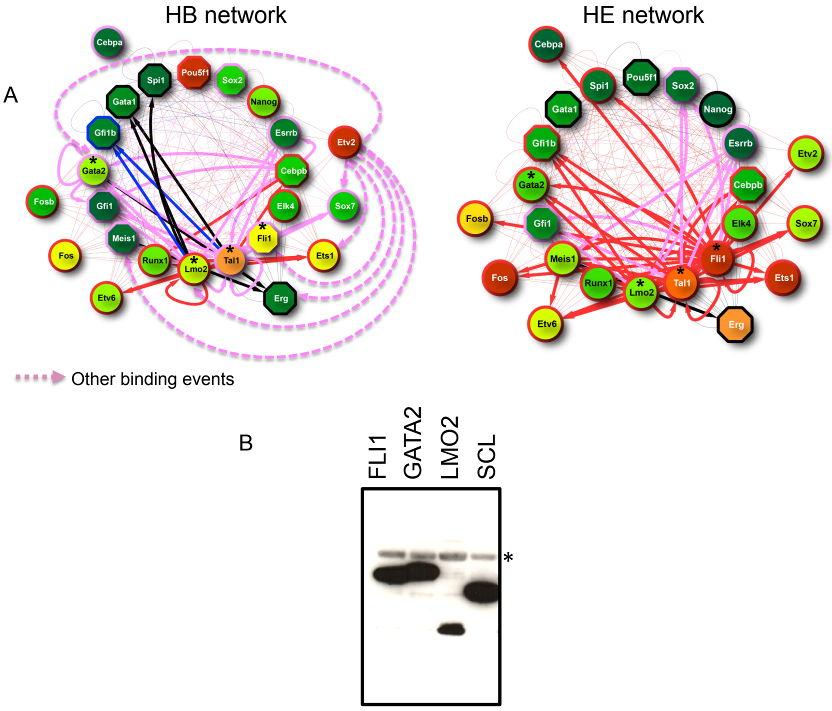

Figure S5 (related to Figure 4 and 5): (A) Expanded gene regulatory networks bordering the major hematopoietic transition T3. The 4 core reprogramming TFs identified in Figure 5 are indicated by asterisks. Additional TFs previously found to influence haematopoietic reprogramming are shown in peripheral nodes with binding events to these loci indicated by arrows. Dashed arrows indicate evidence of reciprocal binding events from independent ChIP-seq experiments at equivalent *in vitro* stages. Evidence from the ETV2-*Sox7* interaction comes from qPCR analyses of HA-tagged Etv2 ChIP experiments from day 3.5 EBs and pull-down assays (Behrens et al 2014). qPCR of *Etv2* null mice indicates that early *Fli1* expression (E8.0) is Etv2 dependent. This is corroborated by luciferase reporter gene assays and targeted mutagenesis of conserved binding sites in the Fli1 promoter and found to be cell-type specific (Abedin et al 2014). ChIP analyses of day 3-3.5 EBs identified an ETV2 binding event 1 kb upstream of the *Lmo2* TSS, corroborated by EMSA (Koyano-Nakagawa et al 2012). **(B)** Transduced cells express all four constructs. MEFs were transduced with indicated FLAG tagged transcription factor expressing viruses. Cells were harvested at day 4 and cell lysates were subjected to western blotting with anti-FLAG antibodies. * Nonspecific band indicating equal loading.

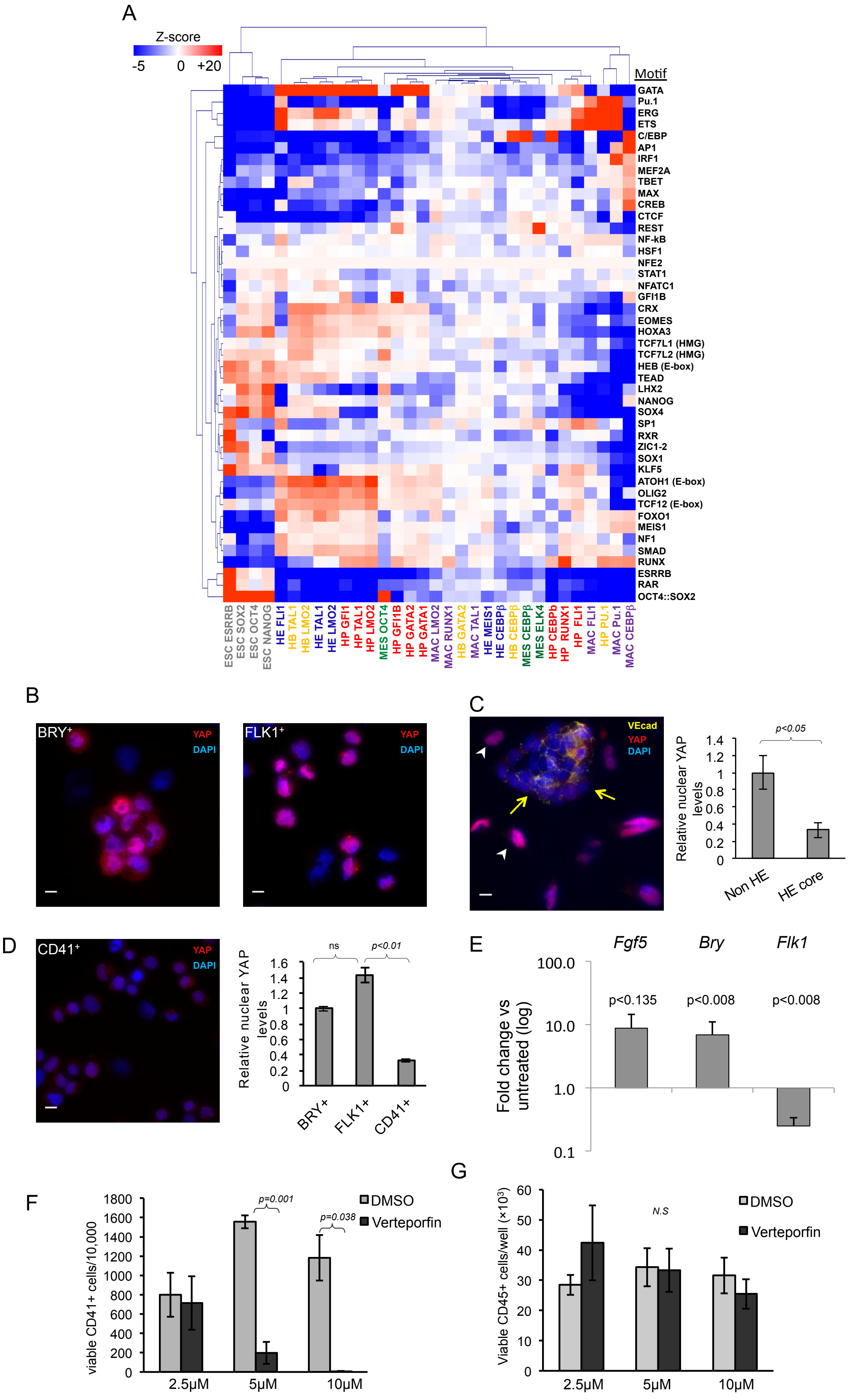

Figure S6 (related to Figure 6 and 7): (A) Relative co-localizing motif frequencies within ChIP peaks of the 32 transcription factors examined in this study (for used motifs see

Table S6). The motif Relative Enrichment (RE) scores within the ChIP peaks of 32 TFs were compared to RE scores of those motifs for a random set of peaks. Z scores were calculated from the mean and standard deviation of random RE scores using bootstrapping analysis. **(B, C & D)** TEAD activity peaks during the early phase of hematopoietic commitment. **(B,D)** Cytospins of EB derived $BRY^{+}/FLK1^{-}$ ME (BRY^{+}), $BRY^{+}/FLK1^{+}$ HB ($FLK1^{+}$) and $CD41^{+}$ HP populations. Cytospins were stained for TEAD co-activator YAP (red) and nuclei were visualised with DAPI (blue), scale bars represent 10µm. Graph shows the quantified relative nuclear YAP levels. Data are presented as mean intensity ± SEM, *n*=5, *t*-test. (**C)** HE culture stained for TEAD co-activator YAP (red). HE cores are identified as VE-cadherin positive (yellow) clustered cells. Relative nuclear YAP levels were calculated for the cells within HE cores (arrow) compared with non-HE surrounding cells (arrow head). Data are presented as mean intensity ± SEM, *n*=5 *t*-test. **(E)** $BRY^{+}/FLK1^{-}$ ME cells were cultured in the presence or absence of verteporfin (9.6 µM). After 48h RNA was isolated and the expression of *Fgf5*, *Bry* and *Flk1* was measured by qPCR. Gene expression in verteporfin treated cells was normalised to expression in DMSO control treated cells. Data for five biological replicates are presented as mean + SEM, Mann Whitney U. **(F)** Inhibition of TEAD – YAP interaction blocks hematopoietic differentiation and survival in explant cultures of cells from early but not late stages of hematopoietic specification. $FLK1^{+}/CD41^{-}$ HE enriched cells were sorted from E7.5 embryos by FACS and cultured for 4 days on irradiated OP9 treated with 2.5µM, 5µM and 10µM verteporfin or DMSO. Hematopoietic differentiation of HE cells was measured by staining with a CD41-PE antibody. **(G)** Verteporfin treatment of $CD45^{+}$ committed hematopoietic progenitor cells sorted from E10.5 embryos by FACS and cultured on irradiated OP9 in hematopoietic cell culture media (see Stroczynsca et al., 2009). Growth and survival of hematopoietic cells was assessed by staining with a CD45-FITC antibody.

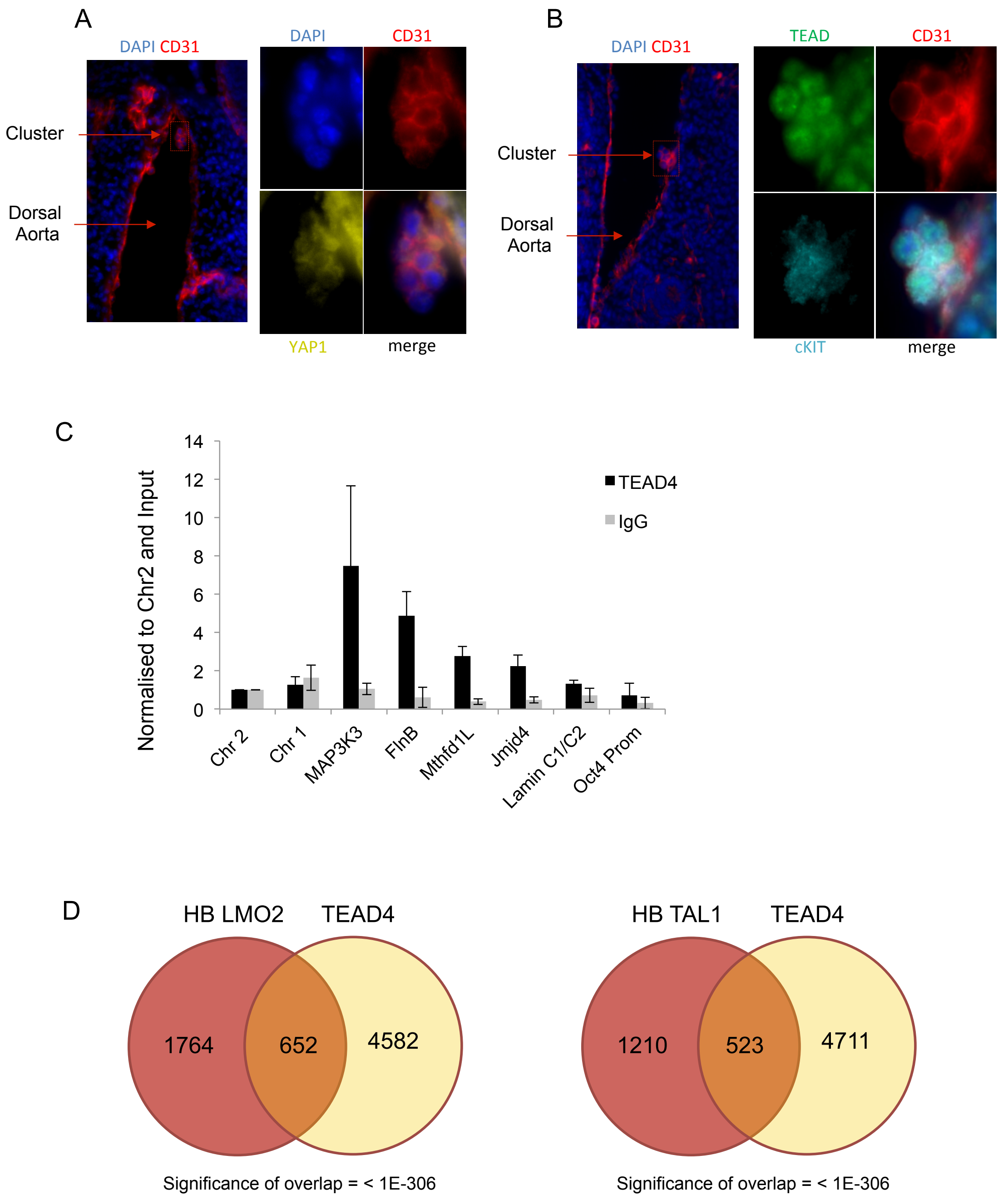

Figure S7 (related to Figure 7): **(A)** TEAD and YAP do not localize to the nucleus of hematopoietic cells in E10.5 dorsal aorta clusters. E10.5 embryo sections were stained as indicated. (A and B) Left panels 40x magnification of DAPI and CD31; right panels 100x magnification of individual and merged stainings as indicated. **(B)** Manual validation of ChIP-Seq experiments. Binding of TEAD4 to TEAD-motif carrying elements (*Map3k3, FlnB, Mthfd1L, LaminC1/C2, JmjD4)* but not to control genes (all others). Data presented are mean ± SD (*n*=3). **(C)** Venn diagrams demonstrating that a significant number of TEAD4 peaks overlap with TAL1 and LMO2 peaks in hemangioblasts (HBs). The significances were calculated as p-values from hypergeometric tests.

Supplementary Tables

2. Table S1 (related to Figure 1, 2)
(A) Antibodies used for ChIP-Experiments and details of sequencing runs, (B) Quality control of ChIP-Seq experiments

3. Table S2 (related to Figures 1, 2, S1, S2):

(A) Genes that are 2 fold differentially regulated during different transitions along the differentiation pathway, (B) Transcription factor genes that are 2-fold differentially regulated, (C) Over-represented GO terms in differentially regulated genes with FPKM >= 1 in at least one cell line

4. Table S3 (related to Figures 1, 2, 3, S1, S2, S4):
(A) Raw expression values of different expression clusters, (B) Enriched GO terms of expression clusters

5. Table S4 (Related to Figures 2 and S2): Enriched GO terms of genes with constitutively active promoters.

6. Table S5 (related to Figure 5): Sequence coverage of ChIP and DNaseI experiments, RNA-Seq data obtained in reprogramming experiments

7. Table S6 (related to Figures 6 and S6): Position weight matrices used for motif clustering

8. Table S7 (related to Figures 6 and S6): Z-scores determined for clustering of motifs enriched in pairwise comparisons of DHSs

9. Table S8 (related to Figures 7 and S7): KEGG pathway analysis of genes associated with TEAD4 peaks

Table S6:

Position Weight Matrices used for motif clustering analyses

LOGO	NAME
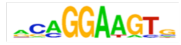	ETS1
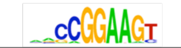	ETS
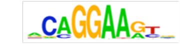	ERG
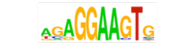	Pu.1
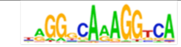	RXR
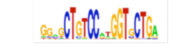	REST
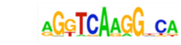	RAR
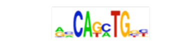	ATOH1
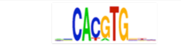	MAX
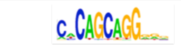	HEB
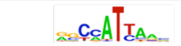	NANOG
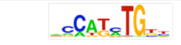	OLIG2
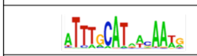	OCT4::SOX2
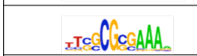	E2F
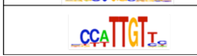	SOX2

LOGO	NAME
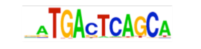	NFE2
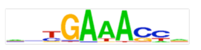	IRF4
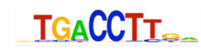	ESSRB
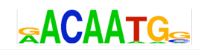	SOX9
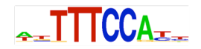	NFATC1
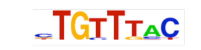	FOXO1
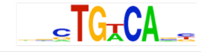	MEIS1
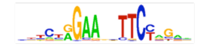	HSF1
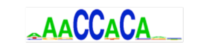	RUNX
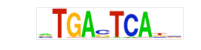	AP1
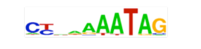	MEF2A
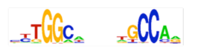	NF1
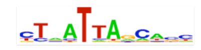	HOXA3
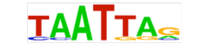	LHX2
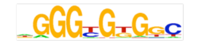	KLF5

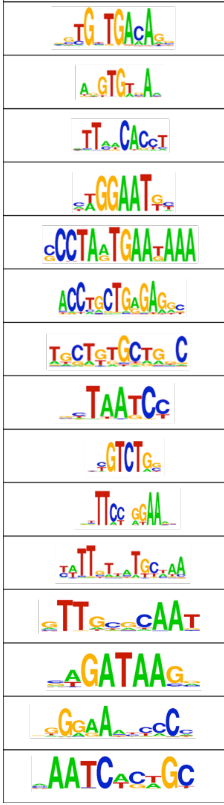

LOGO	NAME
	TBX20
	TBET
	EOMES
	TEAD
	SOX1
	ZIC1-2
	ZIC3-2
	CRX
	SMAD4
	STAT1
	OCT4
	C/EBP
	GATA
	NF-kB
	GFI1B

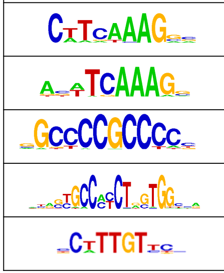

LOGO	NAME
	TCF7L1
	TCF7L2
	SP1
	CTCF
	SOX4

2. SUPPLEMENTAL EXPERIMENTAL PROCEDURES

Purification of cell populations

A mouse ES cell line carrying a Brachyury-GFP reporter gene (Fehling et al., 2003) was cultured on MEFs then differentiated as described previously (Sroczynska et al 2009). Both GFP and cell surface markers were used to isolate each cell population, as outlined in Figure S1A. Briefly, after differentiation of ESC into embryoid bodies, mesodermal cells were isolated by FACs sorting GFP/Brachyury (Bry) positive cells that have yet to express the endothelial marker Flk1. A proportion of these cells were allowed to differentiate towards hemangioblasts (HB, $Bry^+/Flk1^+$), hemogenic endothelium (HE, $Tie2^+/cKit^+/CD41^-$) and hematopoietic progenitors (HP, $CD41^+$). Finally macrophages were isolated by terminal differentiation of $CD41^+$ cells to those expressing the macrophage marker CD11b.

Isolation of cells for TEAD4 ChIP - Seq: A single cell suspension of ES cells was transferred into IVD media on 15 cm low adherence bacteriological plates (Sterilin) at a concentration of 2.5 X 104/ml. IVD media - IMDM supplemented with 15 % FCS, 100 units/ml Penicillin and 100 µg/ml Streptomycin, 1 mM glutamine, 0.15 mM MTG, 0.18 mg/ml Human transferrin (Roche 652202) and 50 µg/ml ascorbic acid. After 3.25 days the embryoid bodies were collected, briefly digested in Tryple-Express protease mix (Life Technologies) and gently dissociated. A single cell suspension was obtained by passing the cells through a cell strainer and resuspending in IMDM + 20 % FCS. $Flk1^+$ (CD309) cells were isolated using a biotinylated Flk1 antibody (eBioscience 13-5821) at 5 µl per 107 cells for 15 minutes on ice, followed by 2 washes with MACS buffer (PBS + 5 % BSA and 0.5 mM EDTA). Cells bound by the antibody were then isolated using MACS anti-biotin beads and MACS LS columns (Miltenyi Biotec) according to the manufacturer's instructions. Isolated Flk1+ve cells were subsequently used for chromatin immunoprecipitation.

Chromatin immunoprecipitation assays

ChIP-Seq: For each stage of development, cells were sorted, crosslinked and stored either as frozen cells (histone modification ChIP) or nuclei (TF ChIP) for subsequent ChIP assays, performed as described in detail previously (respectively Lichtinger et al 2012 and Wilson et al 2009; Forsberg et al 2000). The antibodies used in remaining ChIP assays are given in Table S1. For histone modifications around 2 million cells were used per experiment whereas 6-12.5 million cells were used for each TF ChIP assay, with the

exception of HEs, which constitute a rare transient cell population. In these cases an adapted carrier ChIP protocol (Zwart et al 2013) was used on around 1-2 million cells. Specifically 20 µg/ml recombinant histone 2B (M2505S; New England Biolabs) and 1 µg/ml mouse mRNA (cat# 338114, Qiagen) were added before the antibody at the immunoprecipitation step.

Manual ChIP from Flk1$^+$ hemangioblast cells: Chromatin immunoprecipitation was performed essentially as described in Lichtinger et al 2012 with the exception that the material was crosslinked with Di(N-succinimidyl)-glutarate for 45mins (DSG; Sigma) and 1% Formaldehyde for 10mins (Thermo Fisher) before quenching with 1/10th volume 2 M glycine. Primers used for ChIP analysis are shown in the table below.

Gene	Forward Primer	Reverse Primer
Oct4 promoter	TGGGCTGAAATACTGGGTTC	TTGAATGTTCGTGTGCCAAT
MAP3K3	TCACTGGTCTGGGGAGACTT	CCTTGCTATCTGATGGGCGA
FlnB	TGGGGTAGGGGCCAGATTAG	GACAATGGCTCCCTAGAGCTT
Jmjd4	TGCAAGCTTCGACAATGACC	TGCAGCTACGTACTCTTCCC
Lamin C1/C2	TCTCAGGAAACCGACCCATTC	CCCTCCGGTGAGTGTGAAAG
Mthfd1L	ATGCAAGCTGGGCAAATTCC	CCCAGAGTGCGGACAAAGAA

Reprogramming experiments: Embryonic day 14.5 murine embryonic fibroblasts (MEFs) were prepared as described previously (Sroczynska et al., 2009). Cells were isolated from iScl-2A-GFP transgenic mouse carrying rtTA and TRE-SCL cassettes allowing inducible expression of SCL upon addition of doxycycline. Cells negative for CD45/CD41/CD31/CD117 were sorted from low passage MEFs and seeded on gelatine coated 12 well plates (1.5x10^4 cells/well). The next day the cells were transduced with different combinations of lentiviral vectors expressing FLI1, GATA2 or LMO2 in the presence of 10µg/ml DEAE-dextran. After four hours, cells were placed in haematopoietic medium (1X IMDM supplemented with plasma-derived serum (PDS; Antech), 10% protein-free hybridoma medium (PFM; Gibco), 0.5 mM Ascorbic Acid, 4.5x10^{-4} M MTG, 2 mM L-glutamine, 80 mg/ml transferrin, 1% c-KIT ligand, 1% IL3, 1% of GM-CSF and 1% Thrombopoietin conditioned media, 4 U/ml Erythropoietin (Ortho-Biotech), 10 ng/ml M-CSF, 10 ng/ml IL6, 5 ng/ml IL11 (all from R&D Systems) and 50 µg/ml penicillin-streptomycin). Doxycycline (1µg/ml) was also added in some cases to induce SCL

overexpression and was replenished on days 5 and 9 to achieve continuous expression of SCL. Cultures were incubated at 37°C in 5%$C0_2$ for 19 days at which point the number of reprogrammed blood cell colonies was scored.

Immunostaining of in vitro differentiating cells: $BRY^+/FLK1^-$ ME, $BRY^+/FLK1^+$ HB and $CD41^+$ HB populations were sorted from day 3, 4 and 7 EB cultures respectively, and adhered to slides by cytospin (5 minutes 400 rpm). In order to generate HE, day 3 EB-derived $FLK1^+$ cells were cultured for 48 hours on gelatinised µ-Slides (Ibidi). Cells were fixed with 2% paraformaldehyde (Sigma), permeabilised in PBS 0.2% Triton X-100 (Sigma), washed and stained with PBS, 10% FCS, 0.2% Tween 20 (Sigma). Cells were pre-blocked for 15 minutes with PBS, 10% horse serum and incubated with 1:100 anti-YAP (YAP_D8H1X XP® #14074 NEB) overnight at 4°C, followed by anti-rabbit AF647 or AF488 secondary antibodies (both Invitrogen; 1:1000 dilution) for 1 hour. HE cores in blast culture were identified by staining with anti-VEcadherin AF647 conjugated antibody (Alexa Fluor® 647 anti-mouse CD144 # 138005, Biolegend) overnight at 4°C. Slides were mounted with ProLong Gold antifade reagent with DAPI (Invitrogen). Images were captured using an Axiovert 200M (Zeiss) at 40x magnification, and nuclear YAP levels were calculated using Image J software as previously described (Arques et al., 2012).

Immunostaining of embryos

E7.5 in decidua and E10.5 embryos were fixed in 4% Paraformaldehyde (PFA) for two hours, soaked in 30% sucrose and mounted in OCT compound. 7µm sections were blocked in PBS with 10% FCS, 0.05% Tween20 and 10% goat serum (DAKO) for 1 hour before the sections were incubated with primary antibodies at 4°C overnight in blocking buffer. Primary antibodies used in this study were rabbit Anti Pan-TEAD (D3F7L) (1/100); rabbit Anti YAP (D8HIX XP) (1/100); Purified Anti-Mouse Tie2 (Tek-CD202B) (14-5987-85) (1/100). Sections were washed 3x in PBST for 15 minutes each and then incubated with fluorochrome-conjugated secondary antibody at room temperature for 1 hour. Secondary antibodies used in this study include Alexa Fluor® 488 Goat Anti-Rat IgG (A11006, Life Technologies); Alexa Fluor® 647 $F(ab')_2$ Fragment of Goat Anti-Rabbit IgG (H+L) (A21246, Life Technologies). All secondary antibodies were used at 1/400 dilution. Sections were further washed 3x in PBS and mounted using Prolong Gold anti-fade medium with DAPI (Life Technologies). Images were taken using a low-light time lapse microscope (Leica) using the Metamorph imaging software and processed using ImageJ.

Inhibition of TEAD – YAP interaction with Verteporfin during ES cell differentiation.

The effect of TEAD inhibition during hematopoietic development was analysed in EB cultures in the absence or presence of the TEAD-YAP inhibitor Verteporfin (Liu-Chittenden et al., 2012, Brodowska et al., 2014) (Sigma). Verteporfin (stock solution 2.7 mM in DMSO) was added to day 1, 2, 3 or 4 EB cultures at a final concentration of 9.6μM. On day 7, EBs were disrupted with trypsin, stained with CD41-PE and CD45-brilliant violet 421(BD) and analysed by flow cytometry (LSRII BD). Cells were protected from light at all times. The $BRY^{+}/FLK1^{-}$ mesoderm enriched cell population was obtained from day 3 EB cultures using FACS and was re-plated in EB media in Ultra-Low Attachment plates (Corning/CoStar) at a concentration of 2E+5 cells / ml. The cells were cultured for 48 hours in the presence of 9.6 μM Verteporfin (Sigma) or vehicle (DMSO). Upon Verteporfin treatment the cells were protected from light at all times. RNA was extracted using the Spin Column RNA miniprep kit (NBS Biologicals). Complementary DNA was prepared using SuperScript III First-Strand Synthesis (Invitrogen). Quantitative PCR was performed using the Universal Probe Library System (Roche) using the following primers and probes: Fgf5 forward 5'-GTTTCCAGTGGAGCCCTTC-3', Fgf5 reverse 5'-GAGACACAGCAAATATTTCCAAAA-3' with probe 105; Bry forward 5'-CAGCCCACCTACTGGCTCTA-3', Bry reverse 5'-GAGCCTGGGGTGATGGTA-3' with probe 100; Flk1 forward 5'-AAAGCGGGACGAGGAGAG-3', Flk1 reverse 5'-CAGGTTGCACAGTAATTTCAGG-3' with probe 12.

Inhibition of TEAD – YAP interaction *ex vivo*

$FLK1^{+}/CD41^{-}$ HE enriched cells were sorted from E7.5 embryos by FACs and cultured for 4 days on irradiated OP9 in HE media (IMDM containing 10%FBS, 4mM L-Glutamine, 50Units/ml Pen/Strep, 0.6% transferrin, 2% LIF, 48.75μg/ml monothiolglycerol, 25 μg/ml ascorbic acid, 2%LIF, 1%KL supernatant, 10ng/ml oncostatin M, 1ng/ml bFGF and 1μg/ml osteopontin) with 2.5μM, 5μM and 10μM verteporfin or DMSO. Hematopoietic differentiation of HE cells was measured by staining with a CD41-PE antibody and flow cytometry (LSRII BD). $CD45^{+}$ committed hematopoietic cells were sorted from E10.5 embryos by FACs and cultured on irradiated OP9 in hematopoietic cell culture media (see Stroczynsca et al., 2009) with increasing concentrations of verteporfin or DMSO. Growth and survival of hematopoietic cells was assessed by staining with a CD45-FITC antibody before the addition of counting beads (Thermo Fisher) and subsequent flow cytometry analysis. The number of viable $CD45^{+}$ cells/well was calculated by the ratio of viable

CD45$^+$ cells to counting beads according to the manufacturer's instructions (Thermo Fisher).

RNA-Seq

RNA preparation and sequencing were performed as described previously (Lie-A-Ling et al 2014), generating 50bp single-end reads from a SOLiD 5500xl sequencer (#4456991, Life Technologies).

DNaseI-seq

1-3 million freshly sorted cells were digested with DNase I enzyme as described previously, and size selected for 50 - 300 bp fragments as described in detail in (Ptasinska et al., 2012).

Library generation and Sequencing

DNaseI and ChIP samples were amplified and sequenced according to the manufacturer's instructions. Initial samples (MES, HB and HP H3K4me3, HP H3K27me3 and HP H3K27ac) were processed using an ABI SOLiD 4 sequencer, for all other libraries sequencing was conducted on either the Illumina 2G Genome Analyzer,the Hi-Seq 2000 or the Hi-Seq 2500.

DATA ANALYSIS

Alignment

Sequences from all experiments were mapped onto the reference mouse genome version mm10, Genome Reference Consortium GRCm38. The adapters were removed from reads from the Illumina sequencer using CutAdapt (http://code.google.com/p/cutadapt/) and trimmed for low quality reads (less than 20). The quality control statistics for the reads were obtained using FastQC (http://www.bioinformatics.babraham.ac.uk/projects/fastqc/). The trimmed Illumina reads were aligned to the mouse genome using Bowtie (Langmead et al., 2009). The colour-space reads from the SOLiD sequencer (see Table S1) were aligned using SHRiMP (Rumble et al., 2009). The total number of aligned reads for all the experiments in this study is given in Table S5. Reads that were aligned to unique chromosomal positions were retained and duplicate reads were removed from the aligned data using Picard (http://broadinstitute.github.io/picard/). All further calculations were

performed on the filtered alignment data. The aligned RNA-seq and ChIP-seq reads were extended by 200bp from their 3' ends to generate density profiles in BigWig format. In the case of DNaseI-seq data, only the 5' ends of aligned reads were used for generating the density profiles. Separate density profiles for the positive and negative strand were generated for RNA-seq data. These tag densities can be viewed from our local instance of the UCSC genome browser (www.haemopoiesis.leeds.ac.uk).

Peak Calling

TF peaks were called using MACS (Feng et al 2011; Zhang et al 2008), whereby the p-value cutoffs were manually assessed after visually inspecting known reference genes (see Table S1). The quality of TF ChIP-seq data was further refined using the polyaPeak R-package (Wu and Ji, 2014). Enriched regions of histone acetylation (H3K27ac, and H3K9ac) were obtained using DFilter (default parameters) and MACS and the coordinates from DFilter (Kumar et al., 2013) that overlap with MACS peaks were considered as acetylation peaks for subsequent analyses. For each cell population, the respective ChIP inputs were used as control data during peak calling. DNaseI Hypersensitive sites (DHSs) are those that are called as peaks by DFilter (default parameters) in the DNaseI-seq data. The total number of TF and histone modification peaks and DHSs in the dataset is provided in Table S1.

RNA-seq data analysis

Identification of differentially expressed genes

The total number of tags that overlap with the genes (exonic region only) were obtained using HTSeq (Anders et al., 2015) and differential expression analysis for the coding genes were performed using DESeq (Anders and Huber, 2010; Anders et al., 2013) using the count data. Genes were said to be differentially expressed (DE) if there was a twofold change in expression between any two cell lines with a *p*-value less than or equal to 0.01. Over-represented Gene Ontology (GO) terms (Ashburner et al., 2000) identified using DAVID (Huang da et al., 2009a, b) as well as gene family classification (MSigDB (Liberzon et al., 2011)) can be seen in Table S2. We identified 8986 genes to be differentially expressed between any pair of cell types from our DESeq analysis (Figure S1E) that also have an expression value of at least 1 FPKM (from cufflinks) in any of our cell types.

Gene expression quantification

Cufflinks (Trapnell et al., 2012) was used to calculate the expression values as Fragments Per Kilobase per Million aligned reads (FPKM) from the aligned RNA-seq data. Both gene-

wise expression values and TSS based expression values were obtained using the *cuffdiff* function of cufflinks. The correlation between any two cell lines was obtained as the Pearson correlation coefficient of expression values over all genes. A correlation matrix was thus generated for all the cell lines and hierarchically clustered to study the relationship among cell lines as given in Figure 1B. The gene-wise expression values for each developmental stage were used to calculate the principal components using *princomp* package from R. The top three principal components were found to contribute to almost 90% (Figure S1D) of the variance observed in the expression data. Figure S1D shows the cell stages projected onto these three principal components.

Clustering of differentially expressed genes

The 9627 unique TSSs belonging to the 8986 DE genes with FPKM greater than 1 in at least one cell population (Table S2) were then clustered into 31 expression patterns (Figure 1F) using *k*-means and hierarchical clustering as follows. For each TSS (*i*) of a DE gene a pseudo-count $\gamma = 0.001$ was added to the TSS-based FPKM values and the binary logarithm of this value was considered as the expression value of the TSS in each cell type (*j*), $e_{ij} = log_2(FPKM_{ij} + \gamma)$. The expression data was further quartile normalised using the *LPE* package in R to give standardised expression values, and then converted to Z-scores ($z_{ij} = \frac{e_{ij} - \mu_i}{\sigma_i}$) where μ_i, is the mean expression and σ_i is standard deviation of the gene "*i*" across all the cell lines for clustering purposes. The Z-scores of the genes (TSSs) were initially clustered using *k*-means clustering with Euclidean distances (*stats* package in R) and the optimal number of clusters was determined to be 650 based on the lowest Bayesian Information Criterion (BIC) scores (Schwarz, 1978). The centroids of the *k*=650 clusters were calculated as the mean of Z scores of the members of the clusters. These centroids were then hierarchically clustered using the "complete linkage" agglomeration method (Lance, 1967) and the tree was cut into 31 patterns.

The GO terms that are over-represented (*p*-values less than 0.05) in genes that are up and down-regulated at different transition points (T1 to T5), and within different expression patterns, were obtained using DAVID and are summarised in Figure 1E, and also in Table S2 and Table S5, respectively. RNA-seq data for each cell-type were performed as replicates and both DESeq and Cufflinks, used in our analysis, are designed to account for the significance of reproducibility among the replicates

Functional network of expression patterns

The top 20 over-represented GO terms with the best *p*-values were identified for each of the 31 expression patterns. Undirected edges were drawn between the pattern and the shortlisted GO terms, and therefore GO terms that are shared among expression patterns will have a vertex degree of greater than one. Cytoscape (Cline et al., 2007) was used to generate a visual representation of the network using an edge-weighted spring embedded layout consisting of expression patterns and the associated GO terms (Figure S4A; http://www.haemopoiesis.leeds.ac.uk/data/networks/goNet.svg).

DNaseI-seq data analysis

The 50bp reads were aligned to mouse genome and the bigWig files were generated for visualisation as mentioned earlier. DNaseI-seq peaks or DNaseI Hypersensitive Sites (DHSs) were obtained using DFilter with recommended parameters (-bs=100 -ks=50 –refine). Pair-wise peak overlaps between DHSs of any two cell lines *a* and *b* were performed using BedTools (Quinlan and Hall, 2010) in order to calculate the fraction (M_{ab})

$$M_{ab} = \frac{N_{ab}}{N_a}$$

where N_{ab} is the total peaks that overlap (minimal overlap of 1bp), N_a is the total number peaks in set *a* and N_b is the total peaks in *b*. Given N_a, N_b, N_{ab} and the total DHSs in all cell lines, a hypergeometric test was used to compute the statistical significance of the overlap. It should be noted that M is an asymmetric matrix as $M_{ab} \neq M_{ba}$. M_{ab} is not considered, i.e. M_{ab}=0, if the p-value for the overlap is greater than 0.05, thereby generating a corrected overlap matrix *M'*. Pearson correlation coefficients were calculated between any two rows of the matrix *M'* and this resultant correlation matrix was hierarchically clustered. Finally, the R package cba was used for optimal ordering of nodes in the dendrogram and a heatmap was generated using the gplots R package and presented in Figure 1D.

Clustering of DHSs based on their binary patterns

The DHSs from all cell lines were standardised to 500 bp around the summit and merged using BedTools (Quinlan and Hall, 2010) if DHS sites overlap by at least 125bp, to form a master set of 96195 regions. The dynamics of chromatin accessibility is captured as binary (open=1; closed=0) clusters of DHSs. Each of the merged regions was given in a binary code of 6 digits based on the presence (*1*) or absence (*0*) of DHS in all the six cell types. For example, DHS_100000 is a hypersensitive site that is open only in ESC while DHS_000011 denotes a site that is open both in HP and MAC. All 96195 merged regions were then clustered based on their binary patterns and Figure 2D shows the most

predominant patterns (population size of the pattern greater than 100). A hypersensitive site is deemed as "distal" if it is greater than 2kb from the TSS of the nearest coding gene. *De novo* motifs were identified for the distal DHS sites and matched to known motifs using HOMER (Heinz et al., 2010). A motif was termed as over-represented if its *p*-value was less than 0.01 and its frequency of occurrence in the peaks (HOMER default cutoff) was at least 10%. Over-represented motifs obtained from stage specific distal DHSs (DHS_100000, DHS_010000, DHS_001000, DHS_000100, DHS_000010 and DHS_000001) were used to assemble a master set of PWMs for interrogating all distal DHSs (Table S6). Figure 6B shows the hierarchically clustered relative enrichment (RE) scores for each of these motifs across various DHS sets. RE scores were calculated as given below.

Motif identification and clustering

To identify motifs (identified by HOMER as above) that are relatively enriched in the distal DHS sites of one cell type compared to another we considered all possible cell-type to cell-type comparisons (Figure 6A). For each cell type (A) we defined 5 sets of DHS sites, these being DHS sites in (A) which are not shared with each of the 5 other cell types (B). For 6 cell types (A) this yields a total of 6x5=30 sets of DHS sites. For a given set *j* of DHSs (in Figure 4A), we defined a Relative Enrichment (RE) score for motif *i* in peak set *j* as

$$RE_{ij} = \frac{n_{ij}/M_j}{\sum_j n_{ij}/\sum_j M_j}$$

where n_{ij} is the number of peaks in each subset *j* *(j=1,2,…,30)* containing motif *i* *(i=1, 2,….,I)*, *I* is the total number of motifs used in the test, and M_j the total number of peaks in each subset *j* *(j=1,2,…,30).* A matrix was generated and the relative motif scores were displayed as a heatmap after hierarchical clustering with Euclidean distance and complete linkage. The heatmap was generated using Mev from TM4 microarray software suite (Saeed et al., 2006). The relative motif enrichments were also calculated in similar way within the TF ChIP-Seq peak sets (Figure S6A). A matrix was generated and the motif relative enrichments were displayed as a heatmap after hierarchical clustering with Euclidean distance and complete linkage. The TEAD motif co-localization within the ChIP-Seq peaks (Figure S6C) shows the log2 enrichment score for visualization. The statistical significance for a RE_{ij} score of a given motif *i* in peak set *j* is computed as Z-scores using bootstrapping (N=1000), where a random set of peaks is extracted from a global set of regions and *RE* is calculated. After N iterations the mean (μ_{ij}) and the standard deviation (σ_{ij}) are computed and the z-scores are computed as $Z_{ij} = \frac{RE_{ij} - \mu_{ij}}{\sigma_{ij}}$. The global set of

regions is a merged set of all the DHS for Figure 6B and a merged set of all TF peaks in Figure S6A. These Z-scores are provided in Table S7.

ChIP-seq data analysis

The single-end 50 bp reads were aligned to the mouse genome as mentioned earlier. The TF peaks were called using MACS (Feng et al 2011; Zhang et al 2008) and were standardised to 400bp around the peak summits. Fractional overlap matrix (*M'*) was calculated for the TF ChIP-seq peak sets similar to the methodology used for computing correlation among DHSs, as mentioned in DNaseI-seq analysis section. For robust analyses, correlation values among the TF ChIP-seq peaks were calculated from binding events that occurred in at least two different experiments. The hierarchically clustered correlations among the different TF binding sites (TFBS) are shown in Figure 1D.

The histone mark peaks were identified using DFilter with the following recommended parameters. For H3K27ac and H3K9ac the peaks were called using -bs=100 -ks=60 -std=2, while for H3K27me3 peaks were called using -bs=100 -ks=30 -nonzero -std=2, and H3K4me3 peaks were called using -bs=100 -ks=100 -std=2 options. The total peaks in each experiment are given in Table S1. The H3K27ac peaks were further refined as the consensus peak regions of both DFilter and MACS (default parameters) and this peak set is used as in our integrated data viewer.

http://www.bioinformatics.leeds.ac.uk/labpages/hematopoiesis/)

The average tag density profiles of the histone marks around the TSSs of coding genes, given in Figure S2A, were calculated as a function of the expression values of the TSS as given below.

Average tag density profile of histone marks

The average tag density profiles for each histone mark in a given cell line, around the TSS were generated by calculating the mean tag density normalised as coverage per million (CPM), within 2kb of the TSSs of all genes. The TSSs were grouped into different classes based on their expression values at each developmental stage. For example, a TSS is classified as 1 if its expression value e_{ij} defined above on log scale lies between 1 and 2, 2 if its expression value lies between 2 and 3 and so on until 10 which are given to any expression value greater than 10. The same classification applies to the negative scale of the expression values. The tag density profiles were computed individually for each expression class and rolling averages over 50bp windows were used to generate the

density profiles. The rolling averages were computed using *zoo* and the profiles were plotted using *ggplot2* in R.

Chromatin state identification

The chromatin states were learned using the DNaseI-seq and the histone marks ChIP-seq alignment data using chromHMM (Ernst and Kellis, 2012). The optimal number of states were derived from BIC scores (for three random initialisations, and 5000 iterations with a convergence value of less than 0.001) calculated as $-\ln L + k\ln(n)$ where $k = s(s+5)$, s is the number of states = $\{1, 2, .., 31\}$, n is the total number of bins the genome was divided into and $-\ln L$ is the log likelihood estimation obtained from chromHMM. The lowest BIC score was obtained for 23 states in all the three random initialisations. Based on the emission probabilities of the 23 optimal states, the states were further manually coarse-grained into four, namely the Active, Poised, Repressed or None (Figure S2B).

Promoter state clustering

The fractional contribution profile (FCP) of each of the four states to a promoter in a given cell line is obtained as given below (Figure 2A and Figure 2B). A promoter is defined as ±1kb from the TSS and the fractional occurrence of each of the four states is computed as the fraction of the total number of bases designated as Active, Poised, Repressed or None and is termed as Fractional Contribution Profile (FCP). FCPs were calculated for all the promoters at each stage of development and were used to hierarchical cluster the TSSs with the "complete linkage" method, based on euclidean distances. A single predominant state (with at least 30% occupancy) was assigned to each promoter and colour coded in the heatmap (Figure 2B left panel, Figure S2D). The standardised expression values (z) of the genes belonging to promoters that are always active (Figure 2B; right panel) were hierarchically clustered and the tree was cut into 10 different sub-clusters. The functional annotations of these clusters are given in Figure S2D (and in Table S3).

Integration of 4-dimensional data

Comparison of TF binding events and chromatin accessibility

The binary DHS patterns were obtained as given earlier and plotted with heatmap.2 (gplots package in R http://www.r-project.org/). Genomic coordinates of these patterns were intersected with the TF binding events that occurred in at least two different experiments using BedTools. The statistical significances of such overlaps were calculated as *p*-values using hypergeometric testing. Significant overlaps (p-value < 10^{-4}) were

overlaid onto the DHS heatmap as a dot plot (geom_point function from the ggplot2 package in R) and given in Figure 2D.

Integration of chromatin states at the promoter and expression pattern

Figure S2D describes the correlation between promoter states and expression patterns. The promoters were clustered if they have same Active/Repressed/Poised/None state in the six cell lines. In a similar process to Gene Set Enrichment Analysis (GSEA), the statistical significance of overlap between a given set of genes with the same promoter state pattern and the genes of an expression pattern is computed using hypergeometric testing (*stats* package from R). Figure S3A similarly shows the correlation between chromatin states of distal DHSs and expression patterns. A merged set of distal DHSs were taken and clustered based on their predominant chromatin state (see "Promoter state clustering" section). The statistical significance of the overlap between a set of distal regions with the same chromatin state pattern, and the expression pattern is computed similar to Figure S2D.

Correlation of TF binding events, DHS patterns, Histone H3K27 acetylation and gene expression dynamics

TF binding events with significant overlap (p-value < 10^{-4}) with the loci associated with 31 the gene set expression patterns were identified using Gene Set Control Analysis (GSCA) (Joshi et al., 2013). These data were overlaid as dotplots (see above) onto an expression heatmap (Figure 3B). The H3K27ac enrichment was calculated as the mean of the total number of H3K27ac tags (X) that overlaps with the TF binding events. After bootstrap analyses (5000 iterations of random sampling) z-scores were calculated as $\frac{X-\mu}{\sigma}$ where μ is the average of the mean enrichments over all iterations and σ is the standard deviation. The same analysis was done to examine the correlation between DHS patterns and histone acetylation levels in Figure S3B.

Transcription Factor Gene Regulatory Network

The 16 TFs for which we have ChIP-seq data were considered as nodes and the nodes coloured according to their expression values at each cell line (Figure 4). The shape of the node signifies whether there exists a DHS within 1kb of TSS and border colour signifies whether the promoter is in the state Active, Repressed, Poised or None. A directed edge from TF_a to TF_b indicates binding of a TF_a to the locus of the TF_b and the edge is

prominently displayed if TF_a binds to the locus at that stage. The edge is classified and colour coded according to the promoter state of TF_b.

Annotation data

Genomic definitions for genes annotated as coding genes by RefSeq (Pruitt et al 2014), were obtained using the table browser (Karolchik et al., 2004) from the UCSC genome browser (Kent et al., 2002). The gene names for transcription factors in mouse were obtained from AnimalTFDB (Zhang et al., 2012). Genomic segments that are CG rich were obtained from the Table Browser utility of the UCSC genome browser. The percentage of the DHS_111111 regions that contain one or more CpG island is given in Figure S4B.

Software

R (http://www.r-project.org/) was used extensively for statistical analyses and unless otherwise stated *p*-values were Benjamini Hochberg corrected. The heatmaps in Figure 6B and Figure S6A were generated using MeV (http://www.tm4.org/mev.html). All the peaks in this study were mapped to the TSS of nearest genes (RefSeq definitions) using ChIPpeakAnno (Zhu et al., 2010) package from Rusing default parameters. BedTools and GenomicRanges were used for relevant operations involving peaks. Over-represented Gene Ontology (GO) terms were identified using DAVID (Huang da et *al.*, 2009a, b) with GO_FAT_BP ontology and the default set of all mouse genes as the standard background set. Over-represented KEGG pathways for gene sets were obtained using DAVID similar to GO term enrichment analysis. Genes that belong to various families as classified by MSigDB (Liberzon et al., 2011), are preferentially highlighted in the GO tables given in Table S2, Table S3 and Table S4. The networks in Figure 4, Figure S5 and Figure S4A were generated using Cytoscape.

Supplementary References

Abedin MJ, Nguyen A, Jiang N, Perry CE, Shelton JM, Watson DK, Ferdous A. (2014). Fli1 acts downstream of Etv2 to govern cell survival and vascular homeostasis via positive autoregulation. Circ Res *114,* 1690-1699

Anders, S., and Huber, W. (2010). Differential expression analysis for sequence count data. Genome biology *11*, R106.

Anders, S., McCarthy, D.J., Chen, Y., Okoniewski, M., Smyth, G.K., Huber, W., and Robinson, M.D. (2013). Count-based differential expression analysis of RNA sequencing data using R and Bioconductor. Nature protocols *8*, 1765-1786.

Anders, S., Pyl, P.T., and Huber, W. (2015). HTSeq-a Python framework to work with high-throughput sequencing data. Bioinformatics *31*, 166-169.

Arqués O, Chicote, I., Tenbaum, S., Puig, I., and Palmer, G., H. (2012) Standardized relative quantification of immunofluorescence tissue staining. Protocol Exchange doi:101038/protex 2012008.

Ashburner, M., Ball, C.A., Blake, J.A., Botstein, D., Butler, H., Cherry, J.M., Davis, A.P., Dolinski, K., Dwight, S.S., Eppig, J.T., *et al.* (2000). Gene ontology: tool for the unification of biology. The Gene Ontology Consortium. Nature Genetics *25*, 25-29.

Behrens AN, Zierold C, Shi X, Ren Y, Koyano-Nakagawa N, Garry DJ, Martin CM.(2012). Sox7 is regulated by ETV2 during cardiovascular development. Stem Cells Dev. *23*,2004-2014.

Brodowska K, Al-Moujahed A, Marmalidou A, Meyer Zu Horste M, Cichy J, Miller, JW, Gragoudas E, Vavvas DG (2014). The clinically used photosensitizer Verteporfin (VP) inhibits YAP-TEAD and human retinoblastoma cell growth in vitro without light activation. Exp Eye Res.*124*:67

Cline, M.S., Smoot, M., Cerami, E., Kuchinsky, A., Landys, N., Workman, C., Christmas, R., Avila-Campilo, I., Creech, M., Gross, B., *et al.* (2007). Integration of biological networks and gene expression data using Cytoscape. Nature protocols *2*, 2366-2382.

Ernst, J., and Kellis, M. (2012). ChromHMM: automating chromatin-state discovery and characterization. Nature methods *9*, 215-216.

Fehling, H.J., Lacaud, G., Kubo, A., Kennedy, M., Robertson, S., Keller, G., and Kouskoff, V. (2003). Tracking mesoderm induction and its specification to the hemangioblast during embryonic stem cell differentiation. Development *130*, 4217-4227.

Feng, J., Liu, T., Qin, B., Zhang, Y., and Liu, X.S. (2012). Identifying ChIP-seq enrichment using MACS. Nature protocols 7, 1728-1740.

Forsberg EC, Downs KM, Bresnick EH. (2000). Direct interaction of NF-E2 with hypersensitive site 2 of the beta-globin locus control region in living cells. Blood *96*, 334-339.

Heinz, S., Benner, C., Spann, N., Bertolino, E., Lin, Y.C., Laslo, P., Cheng, J.X., Murre, C., Singh, H., and Glass, C.K. (2010). Simple combinations of lineage-determining transcription factors prime cis-regulatory elements required for macrophage and B cell identities. Mol Cell *38*, 576-589.

Huang da, W., Sherman, B.T., and Lempicki, R.A. (2009a). Bioinformatics enrichment tools: paths toward the comprehensive functional analysis of large gene lists. Nucleic acids research *37*, 1-13.

Huang da, W., Sherman, B.T., and Lempicki, R.A. (2009b). Systematic and integrative analysis of large gene lists using DAVID bioinformatics resources. Nature protocols *4*, 44-57.

Iacovino, M., Chong, D., Szatmari, I., Hartweck, L., Rux, D., Caprioli, A., Cleaver, O., and Kyba, M. (2011). HoxA3 is an apical regulator of haemogenic endothelium. Nat Cell Biol *13*, 72-78.

Joshi, A., Hannah, R., Diamanti, E., and Gottgens, B. (2013). Gene set control analysis predicts hematopoietic control mechanisms from genome-wide transcription factor binding data. Experimental hematology *41*, 354-366 e314.

Karolchik, D., Hinrichs, A.S., Furey, T.S., Roskin, K.M., Sugnet, C.W., Haussler, D., and Kent, W.J. (2004). The UCSC Table Browser data retrieval tool. Nucleic acids research *32*, D493-496.

Kent, W.J., Sugnet, C.W., Furey, T.S., Roskin, K.M., Pringle, T.H., Zahler, A.M., and Haussler, D. (2002). The human genome browser at UCSC. Genome research *12*, 996-1006.

Koyano-Nakagawa N, Kweon J, Iacovino M, Shi X, Rasmussen TL, Borges L, Zirbes KM, Li T, Perlingeiro RC, Kyba M, Garry DJ. (2012). Etv2 is expressed in the yolk sac hematopoietic and endothelial progenitors and regulates Lmo2 gene expression. Stem Cells. 30, 1611-23.

Kumar, V., Muratani, M., Rayan, N.A., Kraus, P., Lufkin, T., Ng, H.H., and Prabhakar, S. (2013). Uniform, optimal signal processing of mapped deep-sequencing data. Nature biotechnology *31*, 615-622.

Lance, G. N., and Williams, W. T. (1967). A General Theory of Classificatory Sorting Strategies
1. Hierarchical Systems. The Computer Journal *9*, 373-380.

Langmead, B., Trapnell, C., Pop, M., and Salzberg, S.L. (2009). Ultrafast and memory-efficient alignment of short DNA sequences to the human genome. Genome biology *10*, R25.

Liberzon, A., Subramanian, A., Pinchback, R., Thorvaldsdottir, H., Tamayo, P., and Mesirov, J.P. (2011). Molecular signatures database (MSigDB) 3.0. Bioinformatics *27*, 1739-1740.

Lichtinger, M., Ingram, R., Hannah, R., Muller, D., Clarke, D., Assi, S.A., Lie, A.L.M., Noailles, L., Vijayabaskar, M.S., Wu, M.*, et al.* (2012). RUNX1 reshapes the epigenetic landscape at the onset of haematopoiesis. Embo J *31*, 4318-4333.

Liu-Chittenden Y, Huang B, Shim JS, Chen Q, Lee SJ, Anders RA, Liu JO, Pan D. (2012) Genetic and pharmacological disruption of the TEAD-YAP complex suppresses the oncogenic activity of YAP. Genes Dev.*26*, 1300-5.

Olive, M., Williams, S.C., Dezan, C., Johnson, P.F., and Vinson, C. (1996). Design of a C/EBP-specific, dominant-negative bZIP protein with both inhibitory and gain-of-function properties. J Biol Chem *271*, 2040-2047.

Pruitt KD, Brown GR, Hiatt SM, Thibaud-Nissen F, Astashyn A, Ermolaeva O, Farrell CM, Hart J, Landrum MJ, McGarvey KM, Murphy MR, O'Leary NA, Pujar S, Rajput B, Rangwala SH, Riddick LD, Shkeda A, Sun H, Tamez P, Tully RE, Wallin C, Webb D, Weber J, Wu W, DiCuccio M, Kitts P, Maglott DR, Murphy TD, Ostell JM. (2014). RefSeq: an update on mammalian reference sequences. Nucleic Acids Res *42*, D756-63

Ptasinska A, Assi SA, Mannari D, James SR, Williamson D, Dunne J, Hoogenkamp M, Wu M, Care M, McNeill H, Cauchy P, Cullen M, Tooze RM, Tenen DG, Young BD, Cockerill PN, Westhead DR, Heidenreich O, Bonifer C. (2012) Depletion of RUNX1/ETO in t(8;21) AML cells leads to genome-wide changes in chromatin structure and transcription factor binding. Leukemia *26*):1829-41

Quinlan, A.R., and Hall, I.M. (2010). BEDTools: a flexible suite of utilities for comparing genomic features. Bioinformatics *26*, 841-842.

Rumble, S.M., Lacroute, P., Dalca, A.V., Fiume, M., Sidow, A., and Brudno, M. (2009). SHRiMP: accurate mapping of short color-space reads. PLoS computational biology *5*, e1000386.

Saeed, A.I., Sharov, V., White, J., Li, J., Liang, W., Bhagabati, N., Braisted, J., Klapa, M., Currier, T., Thiagarajan, M., et al. (2003). TM4: a free, open-source system for microarray data management and analysis. BioTechniques 34, 374-378.

Schwarz, G. (1978). Estimating the Dimension of a Model. 461-464.

Sroczynska, P., Lancrin, C., Pearson, S., Kouskoff, V., and Lacaud, G. (2009). In vitro differentiation of mouse embryonic stem cells as a model of early hematopoietic development. Methods Mol Biol *538*, 317-334.

Trapnell, C., Roberts, A., Goff, L., Pertea, G., Kim, D., Kelley, D.R., Pimentel, H., Salzberg, S.L., Rinn, J.L., and Pachter, L. (2012). Differential gene and transcript expression analysis of RNA-seq experiments with TopHat and Cufflinks. Nature protocols *7*, 562-578.

Wilson, N.K., Miranda-Saavedra, D., Kinston, S., Bonadies, N., Foster, S.D., Calero-Nieto, F., Dawson, M.A., Donaldson, I.J., Dumon, S., Frampton, J.*, et al.* (2009). The transcriptional program controlled by the stem cell leukemia gene Scl/Tal1 during early embryonic hematopoietic development. Blood *113*, 5456-5465.

Wu, H., and Ji, H. (2014). PolyaPeak: detecting transcription factor binding sites from ChIP-seq using peak shape information. PloS one *9*, e89694.

Zhang, Y., Liu, T., Meyer, C.A., Eeckhoute, J., Johnson, D.S., Bernstein, B.E., Nusbaum, C., Myers, R.M., Brown, M., Li, W., et al. (2008). Model-based analysis of ChIP-Seq (MACS). Genome biology 9, R137.

Zhang, H.M., Chen, H., Liu, W., Liu, H., Gong, J., Wang, H., and Guo, A.Y. (2012). AnimalTFDB: a comprehensive animal transcription factor database. Nucleic acids research *40*, D144-149.

Zhu, L.J., Gazin, C., Lawson, N.D., Pages, H., Lin, S.M., Lapointe, D.S., and Green, M.R. (2010). ChIPpeakAnno: a Bioconductor package to annotate ChIP-seq and ChIP-chip data. BMC bioinformatics *11*, 237.

Zwart W, Koornstra R, Wesseling J, Rutgers E, Linn S, Carroll JS.(2013). A carrier-assisted ChIP-seq method for estrogen receptor-chromatin interactions from breast cancer core needle biopsy samples. BMC Genomics *14,* 232
