# Supplementary material for: Dynamic Gene Regulatory Networks Drive Hematopoietic Specification and Differentiation
Source: Dev Cell. 2016 Mar 7;36(5):572–87. doi: 10.1016/j.devcel.2016.01.024 (PMC4780867; doi:10.1016/j.devcel.2016.01.024)
Supplement: Table S1. Antibodies Used for ChIP Experiments and Details of Sequencing Runs; Quality Control of ChIP-Seq Experiments, Related to Figures 1 and 2 [file mmc2.docx]

**Table S1: Related to Figure 1**

1. **Antibodies used for ChIP-Experiments and details of sequencing runs.**

|  | | **Peak Numbers^c^** | | | | | |
| --- | --- | --- | --- | --- | --- | --- | --- |
| **Experiment^a^** | **Antibodies^b^** | **ESC^1^** | **MES** | **HB** | **HE** | **HP** | **MAC** |
| RNA-seq |  | +^s^ | +^s^ | +^s^ | +^s^ | +^s^ | +^s^ |
| DNase-seq |  | 48646 | 21949 | 24729 | 33305 | 17734 | 36456 |
| H3K9ac | Abcam ab4441  & Millipore ABE18 | 104581*^1^ | 92319 | 103340 | 68802 | 51973 | 50076 |
| H3K27ac | Abcam ab4729 | 108485*^1^ | 102827 | 72240 | 53160 | 97254^s^ | 96108 |
| H3K4me3 | Millipore 04-745 | 18185*^1^ | 36394^s^ | 82788^s^ | 93963 | 78049^s^ | 48186 |
| H3K27me3 | Abcam ab6002 | 60652*^1^ | 23048 | 11405 | 266295 | 128006^s^ | 61967 |
| NANOG |  | 15071* |  |  |  |  |  |
| ESRRB |  | 33976* |  |  |  |  |  |
| SOX2 |  | 9937* |  |  |  |  |  |
| OCT4/POU5F1 | Santa Cruz sc8628 | 18189* | 608 |  |  |  |  |
| ELK4 | Santa Cruz sc13030 |  | 524 |  |  |  |  |
| C/EBPβ | Santa Cruz sc150 |  | 1177^3^ | 715 | 132^3^ | 2716^2^ | 55471 |
| SCL/TAL1 | Santa Cruz sc12984 |  |  | 1743^2^ | 2766 | 7097 | 72^2^ |
| LMO2 | R&D systems AF2726 |  |  | 2423^2^ | 3094 | 2976 | 513^3^ |
| GATA2 | Santa Cruz sc267 |  |  | 97^3^ |  | 530 |  |
| FLI1 | Santa Cruz sc356 |  |  |  | 24622^3^ | 3064^3^ | 5492^3^ |
| MEIS1 | Santa Cruz sc10599 |  |  |  | 1282^2^ |  |  |
| GATA1 | AbCam ab11963 |  |  |  |  | 250 |  |
| GFI1 | AbCam ab21061 |  |  |  |  | 1766 |  |
| GFI1B | Santa Cruz sc8559 |  |  |  |  | 2018 |  |
| RUNX1 | AbCam ab23980 |  |  |  |  | 977 | 72^2^ |
| PU.1  TEAD4 | Santa Cruz sc352  Abcam ab58310 |  |  | 5234 |  | 10943 | 82866 |

^a^ List of experiments, with DNase-seq followed by ChIP-seq experiments. ^b^ Relevant antibodies. ^c^ Peak numbers where applicable (number of times sequenced given in superscript if more than once); *Public data. ^1^ ENCODE data. Please note that the antibodies column does not apply to these datasets. (ESC: Esrrb, GSM288355; Nanog, GSM1082342; Pou5f1, GSM1082340; Sox2, GSM1082341). ^+^Two replicates. ^s^ Colour space reads from ABI-SOLiD sequencer. ^2^ Two technical replicates merged. ^3^ Three technical replicates merged.

**(B) Quality control of TF ChIP-seq experiments.**

| Cells | Antibody | qPCR Target seq | fold enrichment | Peak stringency |
| --- | --- | --- | --- | --- |
| MES | C/EBPβ | N/A | N/A | 1e-4 |
| MES | Elk4 | Fos_prom | 13.5 | 1e-5 |
| MES | POU5F1 | Gadd45_prom | 8.8 | 1e-9 |
| HB | C/EBPβ | N/A | N/A | 1e-4 |
| HB | GATA2 | N/A | N/A | 1e-4 |
| HB | LMO2 | Gata2+9.5 | 8.0 | 1e-4 |
| HB | TAL1 | Gata2+9.5 | 44.0 | 1e-5 |
| HE | C/EBPβ | Runx+171 | 6.0 | 1e-9 |
| HE | FLI1 | Lyl1_prom | 8.0 | 1e-3 |
| HE | FLI1 | Pu.1-14 | 6.0 | 1e-3 |
| HE | LMO2 | Lyl1_prom | 18.0 | 1e-4 |
| HE | LMO2 | Pu.1-14 | 4.2 | 1e-4 |
| HE | MEIS1 | Erg+85 | 4.4 | 1e-2 |
| HE | MEIS1 | Lyl1_prom | 5.5 | 1e-2 |
| HE | TAL1 | Lyl1_prom | 64.0 | 1e-6 |
| HP | C/EBPβ | Pu.1-14 | 24.5 | 1e-5 |
| HP | C/EBPβ | Hhex+1.5 | 4.0 | 1e-5 |
| HP | FLI1 | Hhex+1.5 | 13.0 | 1e-5 |
| HP | GATA1 | Nfe2-7.8 | 7.6 | 1e-8 |
| HP | GATA1 | Zfpm1+2.7 | 21.7 | 1e-8 |
| HP | GATA2 | Hhex+1.5 | 3.1 | 1e-6 |
| HP | GFI1 | Lyl1_prom | 8.6 | 1e-6 |
| HP | GFI1B | Erg+85 | 7.8 | 1e-7 |
| HP | LMO2 | Erg+85 | 5.4 | 1e-5 |
| HP | LMO2 | Hhex+1.5 | 37.7 | 1e-5 |
| HP | SPI1 | Hhex+1.5 | 8.6 | 1e-5 |
| HP | RUNX1 | Pu.1-14 | 9.6 | 1e-8 |
| HP | TAL1 | Hhex+1.5 | 8.5 | 1e-4 |
| MAC | C/EBPβ | Hhex+1.5 | 15.5 | 1e-3 |
| MAC | C/EBPβ | PU.1-14 | 222.0 | 1e-3 |
| MAC | FLI1 | N/A | N/A | 1e-4 |
| MAC | LMO2 | N/A | N/A | 1e-5 |
| MAC | SPI1 | Hhex+1.5 | 38.0 | 1e-6 |
| MAC | RUNX1 | N/A | N/A | 1e-7 |
| MAC | TAL1 | N/A | N/A | 1e-4 |

At least two different regions were analysed using qPCR. In general samples were sequenced where there was at least four fold enrichment of target sequence over both IgG and a negative control (inactive) region in chromosome 1. However, since binding events of these TFs have not previously been characterised in the cell types used here, enrichment was not always observed (N/A). As we were confident with the quality of the antibodies, we sequenced these samples regardless. The most stringent p-values for peak calling (right hand column) were determined visually by inspecting multiple regions of the genome.
