# Supplementary material for: Dynamic Gene Regulatory Networks Drive Hematopoietic Specification and Differentiation
Source: Dev Cell. 2016 Mar 7;36(5):572–87. doi: 10.1016/j.devcel.2016.01.024 (PMC4780867; doi:10.1016/j.devcel.2016.01.024)
Supplement: Document S2. Article plus Supplemental Information [file mmc9.pdf]

Resource

Developmental Cell

# Dynamic Gene Regulatory Networks Drive Hematopoietic Specification and Differentiation

## Graphical Abstract

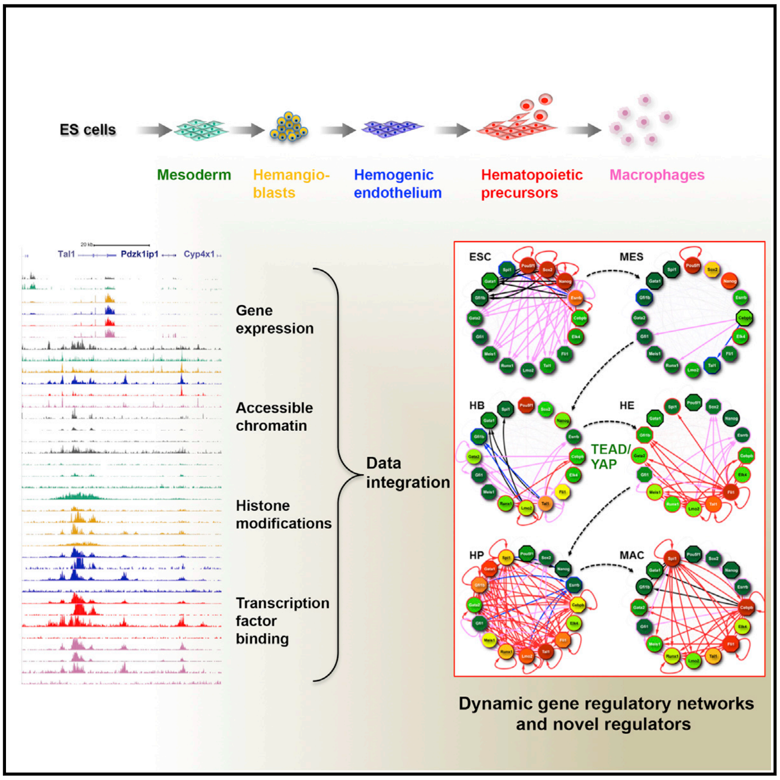


## Authors

Debbie K. Goode, Nadine Obier, M.S. Vijayabaskar, ..., Valerie Kouskoff, Berthold Göttgens, Constanze Bonifer

## Correspondence

c.bonifer@bham.ac.uk

## In Brief

Goode, Obier, Vijayabaskar et al. isolate cells at six different stages of hematopoietic differentiation, starting from embryonic stem cells, and perform a comprehensive multi-omics analysis of this developmental pathway. The data identify regulators of hematopoietic specification and highlight the minimum requirements for the reprogramming of non-blood cells to blood.

## Highlights

- Comprehensive genome-scale resource for studying embryonic blood cell specification
- Genome-scale definition of *cis* elements driving differential gene expression
- A gene regulatory network model for hematopoiesis aiding reprogramming experiments
- Analysis suggests a role for TEAD factors in hematopoietic specification

## Accession Numbers

GSE69101

Goode et al., 2016, Developmental Cell *36*, 572–587
March 7, 2016 ©2016 The Authors
http://dx.doi.org/10.1016/j.devcel.2016.01.024

Developmental Cell
Resource

# Dynamic Gene Regulatory Networks Drive Hematopoietic Specification and Differentiation

Debbie K. Goode,[1,5] Nadine Obier,[2,5] M.S. Vijayabaskar,[4,5] Michael Lie-A-Ling,[3] Andrew J. Lilly,[3] Rebecca Hannah,[1] Monika Lichtinger,[2] Kiran Batta,[3] Magdalena Florkowska,[3] Rahima Patel,[3] Mairi Challinor,[3] Kirstie Wallace,[3] Jane Gilmour,[2] Salam A. Assi,[2] Pierre Cauchy,[2] Maarten Hoogenkamp,[2] David R. Westhead,[4,6] Georges Lacaud,[3,6] Valerie Kouskoff,[3,6] Berthold Göttgens,[1,6] and Constanze Bonifer[2,6,*]

[1]Department of Haematology, Cambridge Institute for Medical Research and Wellcome Trust and MRC Cambridge Stem Cell Institute, Cambridge CB2 0XY, UK
[2]Institute of Cancer end Genomic Sciences, College of Medicine and Dentistry, University of Birmingham, Birmingham B152TT, UK
[3]CRUK Manchester Institute, University of Manchester, Manchester M20 4BX, UK
[4]School of Molecular and Cellular Biology, Faculty of Biological Sciences, University of Leeds, Leeds LS2 9JT, UK
[5]Co-first author
[6]Co-senior author
*Correspondence: c.bonifer@bham.ac.uk
http://dx.doi.org/10.1016/j.devcel.2016.01.024
This is an open access article under the CC BY license (http://creativecommons.org/licenses/by/4.0/).

## SUMMARY

**Metazoan development involves the successive activation and silencing of specific gene expression programs and is driven by tissue-specific transcription factors programming the chromatin landscape. To understand how this process executes an entire developmental pathway, we generated global gene expression, chromatin accessibility, histone modification, and transcription factor binding data from purified embryonic stem cell-derived cells representing six sequential stages of hematopoietic specification and differentiation. Our data reveal the nature of regulatory elements driving differential gene expression and inform how transcription factor binding impacts on promoter activity. We present a dynamic core regulatory network model for hematopoietic specification and demonstrate its utility for the design of reprogramming experiments. Functional studies motivated by our genome-wide data uncovered a stage-specific role for TEAD/YAP factors in mammalian hematopoietic specification. Our study presents a powerful resource for studying hematopoiesis and demonstrates how such data advance our understanding of mammalian development.**

## INTRODUCTION

Cellular identities in multicellular organisms are defined by their individual gene expression programs and are established in a series of cell fate changes starting from pluripotent cells of the embryo. The information on the balanced and coordinated up- and downregulation of gene expression is encoded in our genome and is read by transcription factors (TFs), which interact with the epigenetic regulatory machinery to program the chromatin of lineage-specific genes into active and inactive states. To understand the mechanisms by which TFs establish and maintain specific transcriptional programs, it is essential to investigate developing biological systems, as illustrated by studies in non-vertebrate models (Van Nostrand and Kim, 2011; Zinzen et al., 2009).

Embryonic blood cells arise from early mesodermal cells via hemangioblast and hemogenic endothelial intermediates (Medvinsky et al., 2011). Studies of chromatin programming and gene expression during the generation of mature blood cells from hematopoietic stem cells were instrumental in defining the concept that development at the level of chromatin is a gradual and hierarchical process starting long before the overt transcriptional activation of lineage-specific genes (Bonifer et al., 2008; Hoogenkamp et al., 2009; Org et al., 2015; Wamstad et al., 2012; Wang et al., 2015). This notion is illustrated by the regulatory circuit essential for macrophage differentiation, the gene encoding TF PU.1 (*Spi1*), and its target, the *Csf1r* growth factor receptor gene (reviewed in Bonifer et al., 2008). Both are targets of RUNX1, but *Spi1* expression is induced prior to *Csf1r*. Early *Spi1* induction follows an initial enhancer priming event by TFs upstream of RUNX1 followed by upregulation via autoregulation (Leddin et al., 2011; Lichtinger et al., 2012), whereas subsequent full expression of *Csf1r* requires the concerted action of RUNX1, PU.1, and PU.1-induced factors (Krysinska et al., 2007; Lichtinger et al., 2012). This example illustrates the complexity of the molecular mechanisms underlying the establishment of cell-type-specific expression profiles. However, the global transcriptional control mechanisms underlying such dynamic progression events have remained largely obscure, because of a lack of comprehensive information on TF binding and the dynamic nature of the chromatin template with which they interact. We also know very little about how such transcriptional control mechanisms are interlinked with outside signaling.

The developmental hierarchies of early embryonic hematopoiesis are recapitulated in differentiating embryonic stem cells (ESCs) (Lancrin et al., 2010), which provide a tractable system capable of generating the cell numbers required for performing multiple genome-wide assays on the same samples. Recent studies have investigated the function of individual regulators at specific

developmental stages, such as early mesodermal patterning functions of the TF SCL/TAL1 and the RUNX1-controlled transition from hemogenic endothelium to hematopoietic progenitors (HPs) (Lancrin et al., 2012; Lichtinger et al., 2012; Lie-A-Ling et al., 2014; Liu et al., 2015; Tanaka et al., 2012). However, while a number of studies have examined individual cell fate transitions or investigated the differentiation of mature blood cells from hematopoietic stem cells (Garber et al., 2012; Lara-Astiaso et al., 2014; Tsankov et al., 2015), no study to date has reported an integrated genome-scale analysis of an entire developmental time course from early ESCs to fully defined blood cells.

In this study, we surveyed the global transcriptional journey from the ESC to the terminally differentiated state of macrophages via blood precursor cells by generating data for RNA sequencing (RNA-seq), DNase sequencing (DNA-seq), and chromatin immunoprecipitation sequencing (ChIP-seq) for histone marks and 16 different TFs across six sequential developmental stages. To facilitate access across the wider scientific community, we have integrated all genome-scale datasets into an online resource with advanced browse, search, and analysis capabilities. We have exploited our datasets to assemble a core regulatory network model that was able to inform the design of TF-mediated reprogramming strategies for the production of blood cells from fibroblasts. Furthermore, computational analysis of regulatory elements revealed the nature of TFs involved in stage-specific priming of distal elements, and informed functional validation experiments identifying TEAD/YAP interaction as a stage-specific regulator of early murine blood specification in vitro and in vivo. Finally, we identified TEAD target genes and their associated pathways, thus significantly enhancing our understanding of the signaling processes driving embryonic blood cell development.

## RESULTS

### Capturing a Complete Developmental Pathway using Genome-Scale Technologies

To study the specification of hematopoietic cells and their further differentiation, we employed mouse ESC in vitro differentiation to purify well-defined intermediate cell populations en route from pluripotent ESCs to adherent macrophages (Lancrin et al., 2009; Sroczynska et al., 2009), making use of a *Brachyury* GFP reporter (Fehling et al., 2003) and surface marker expression. Full details of this strategy are given in Figure S1A. In brief, pluripotent ESCs differentiate to mesoderm (MES) cells (Bry:GFP$^{+}$/Flk1$^{-}$), which then progress to the hemangioblast (HB) stage (Bry:GFP$^{+}$/Flk1$^{+}$) with smooth muscle, endothelial, and hematopoietic potential, followed by the hemogenic endothelium (HE) stage that has both endothelial and hematopoietic potential (CD41$^{-}$/Tie2$^{+}$/Kit$^{+}$). HE cells then undergo the endothelial-hematopoietic transition (EHT) involving a shape change, after which they are fully committed to blood (CD41$^{+}$ cells). CD41$^{+}$ cells were further differentiated to generate CD11b$^{+}$ macrophages (MAC). From purified cells we determined global gene expression profiles by RNA-seq and mapped the full set of *cis*-regulatory elements at each developmental stage by global DNaseI hypersensitive site (DHS) mapping (DNaseI-seq). We used ChIP-seq to generate global maps of TF binding for key regulators across this entire developmental pathway as well as global patterns of H3K4me3, H3K9ac, H3K27ac, and H3K27me3 histone modifications to investigate how TFs programmed the chromatin landscape. TFs were chosen according to the cell type in which they were expressed (Figure S1B), and all integrative analysis of ChIP and DHS data was focused on genomic regions found in at least two independent biological experiments (Table S1). Our datasets were complemented with published data for undifferentiated mouse ESCs (Chen et al., 2008; Whyte et al., 2013). The quality of this data resource is exemplified in a browser window depiction of sequence tags aligning to the *Tal1* locus (Figure 1A), which encodes a key regulator of early blood specification (Shivdasani et al., 1995; Wilson et al., 2009).

Initially, we used RNA-seq to investigate the dynamic changes of gene expression across the six differentiation stages and how these changes were reflected in the simultaneous changes in chromatin structure. To this end, we clustered RNA-seq (Figure 1B; Tables S2A and S2B) and DNaseI-seq data (Figure 1C). For both features, samples clustered in line with the known developmental progression, with an early cluster consisting of the ESC, and the more closely related MES and HB and a later cluster made up of HE and HP with the macrophage samples clustering separately. We then performed a similar analysis using TF binding data (Figure 1D). While cell-type-specific clustering of specific TF binding events were evident in ESCs and for certain TFs (e.g. FLI1) in HPs and MACs, others (such as C/EBPβ) showed patterns predominantly driven by the identity of the factor rather than the tissue type (Figure 1D).

To facilitate inspection of individual genes and generate a resource for further data analysis, we developed a web interface to allow streamlined access for the wider scientific community: http://www.haemopoiesis.leeds.ac.uk/data_analysis/. The web portal provides access to both raw and processed data as well as user-driven analysis options. These include queries for specific genes and gene sets across our multi-omics datasets, as well as the visualization of all our data through a custom installation of the UCSC genome browser. In the following sections, we describe how our data can be explored to inform the functional validation of potential mechanisms.

### Identification of the Complete Set of Differentially Active *cis*-Regulatory Elements Driving Hematopoietic Specification

We next inspected the nature of genes changing expression at each cellular transition. 9,627 transcripts from 8,986 genes were dynamically expressed during the developmental time course (Figures S1C–S1E; Tables S2A and S2B). Expression changes between any two sequential developmental stages (transitions T1 to T5, Figure 1E) showed specific enrichment for functionality with the ensuing stage of development for upregulated genes (e.g. T4 shows enrichment for hematopoiesis), and alternative cell fates for downregulated genes (e.g. T4 angiogenesis, heart/muscle development; Table S2C).

To capture dynamic expression patterns across the entire developmental pathway and correlate such changes with alterations in chromatin structure and TF binding, we performed unsupervised/k-means clustering, which identified 31 major expression clusters E1 to E31 (Figure 1F and Table S3A) representing different gene ontology (GO) categories (Table S3B and Figure S4A). For example, E17–E19 represent clusters with

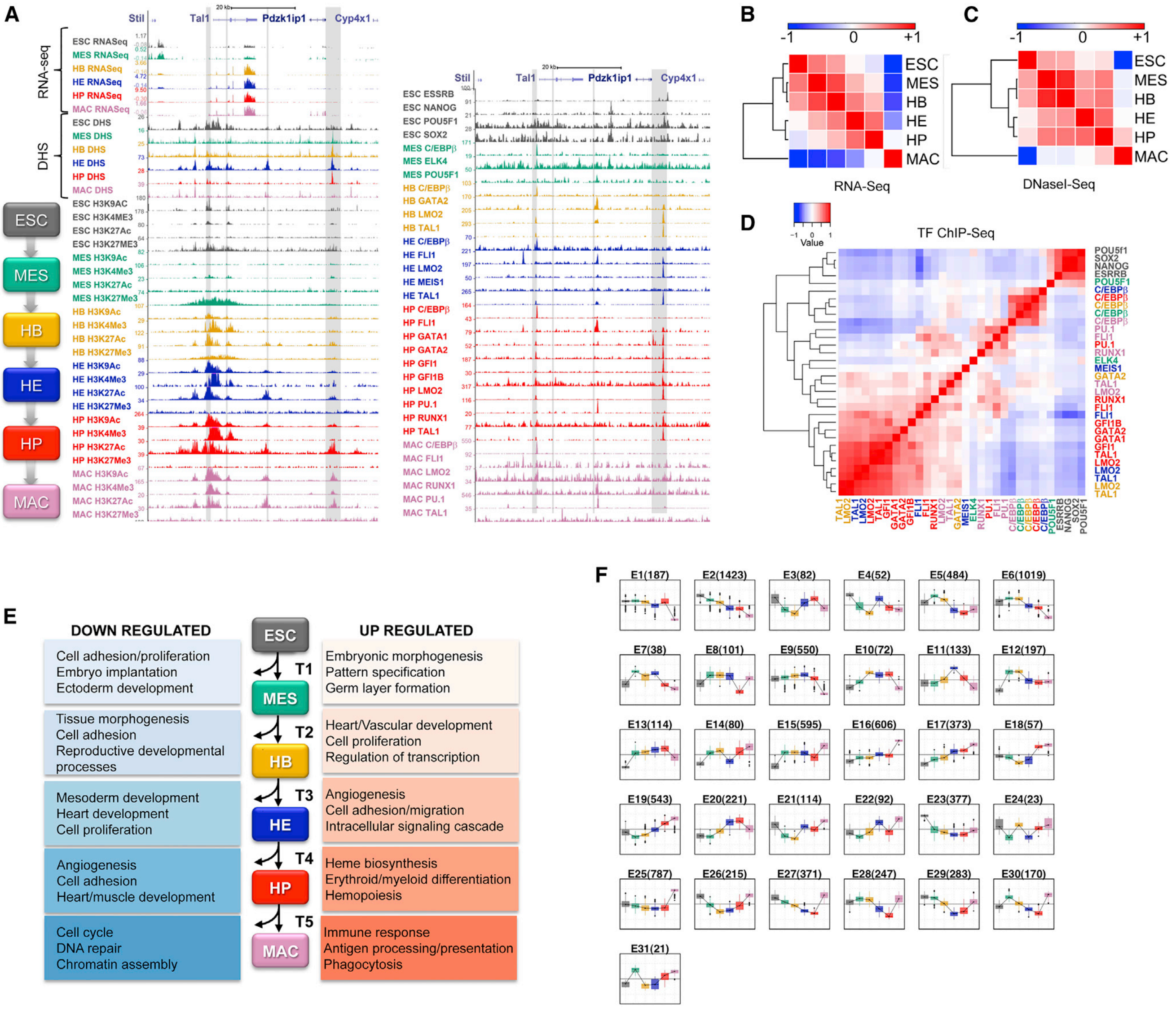


**Figure 1. Integrated Global Data over a Whole Developmental Pathway**

(A) UCSC browser screenshot depicting the *Tal1* locus aligning RNA-seq, DNaseI-seq, and ChIP-seq data from the six stages of development depicted in the left-hand flow chart. The stage-specific color scheme is used in all subsequent figures. Panels display ChIP-seq data for four histone modifications (left) and 16 different TFs (right) plus DHS data. The grayed-out regions indicate known regulatory regions: from left to right, promoters 1a and 1b, enhancers +19 and +40.

(B–D) Hierarchical clustering of cell populations based on the normalized expression values of the genes (B), normalized correlation among the DHS sites (C), and correlation among the TF sites (D). The correlations were normalized between −1 and +1 to preserve the color scale. ESC, embryonic stem cell; HB, hemangioblast; HE, hemogenic endothelium; HP, hematopoietic progenitors, MES, mesoderm.

(E) Functional enrichment for genes that are differentially regulated during developmental transitions (T1–T5) in the progression of hematopoietic commitment.

(F) The expression dynamics of the differentially expressed genes in the pathway given in (A) that are clustered into 31 patterns. The standardized expression values (zij) of the differentially regulated genes in the developmental pathway (Figure S1E) were clustered into 31 expression patterns, and the plot shows the expression profiles of these patterns. The methodology is detailed in Supplemental Experimental Procedures.

increased expression in macrophages, and all are enriched for functions relating to the immune response. Similarly, pattern E11 with upregulation in HE and downregulation in HP is enriched for functions relating to vasculogenesis and adhesion, whereas pattern E20 with upregulation toward HP is enriched for functions relating to hematopoiesis (Figure S4Ai–S4Aiv). Thus, our expression dataset defines distinct gene sets relevant for specific developmental transitions during early blood specification.

We next investigated the correlation between expression kinetics and dynamic changes of chromatin at the gene promoters (Figures 2A and S2A) by using ChromHMM, which was reported as an automated computational system for annotating chromatin states (Ernst and Kellis, 2012). We modified this methodology to integrate both histone modifications and DNaseI accessibility data. The latter indicates regions of chromatin bound by TFs (Cockerill, 2011) and allows for the distinction between inactive

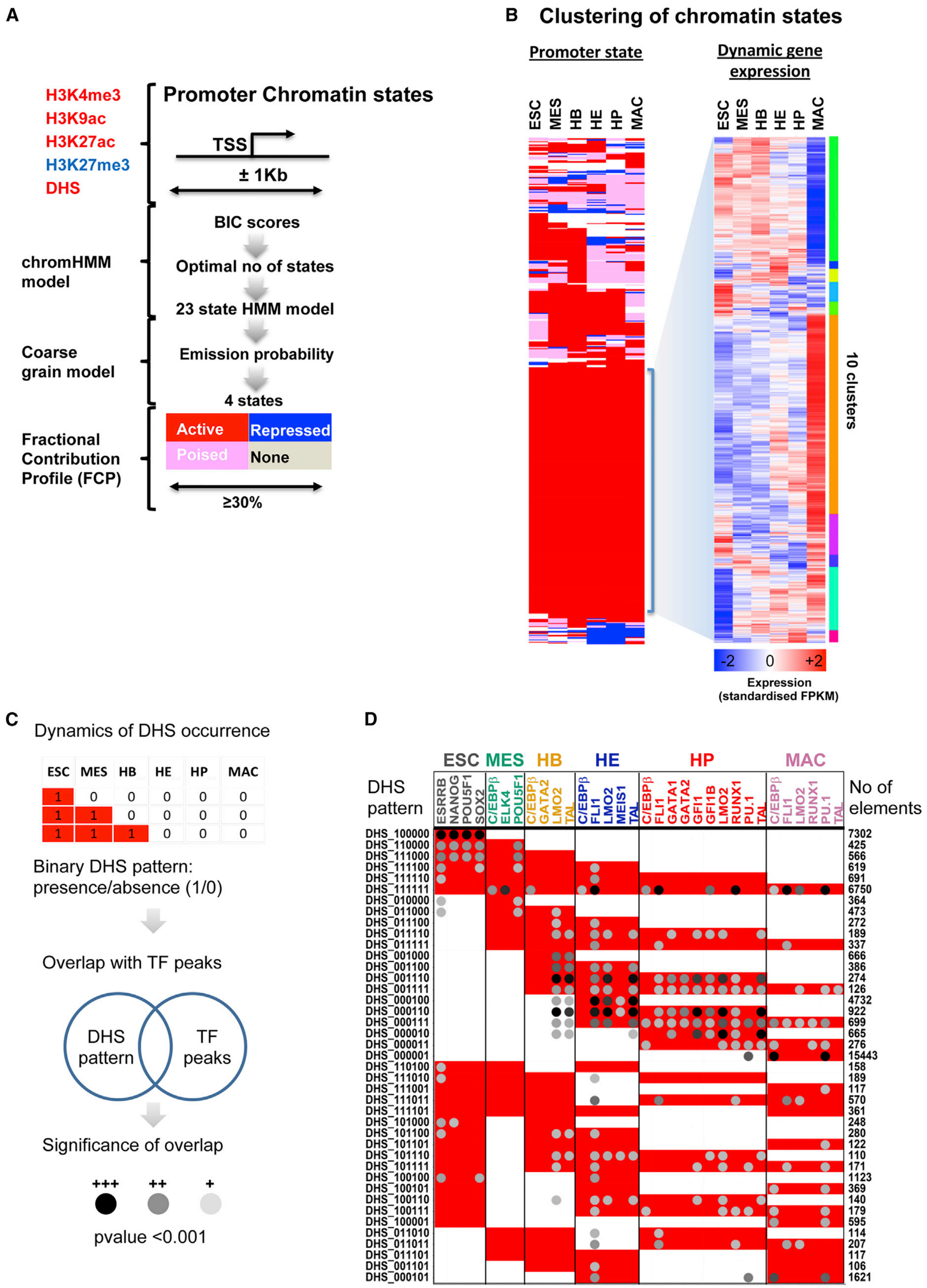


*(legend on next page)*

chromatin regions (absence of DHS) and repressed/poised regions carrying H3K27me3. An initial number of 23 chromatin states (see Figure S2B) was further compressed, providing a simple four-state model of active (DHS marked with H3K4me3 and acetylated H3), repressed (marked with H3K27me3), poised (DHS marked with H3K27me3 but also acetylated H3 and/or marked with H3K4me3), or unmarked chromatin (Figure 2A). Examples (*Nanog*, *Runx1*) for dynamic alterations in promoter state are shown in Figure S2C, demonstrating that such changes occur gradually, both during the transition from the active to the inactive state and during gene activation. This behavior is also evident on a global scale with all differentially expressed genes (Figure S2D). It was proposed that poised promoters of key regulatory genes are held in this state until developmental cues shift the balance from poised to active or repressed states (as in the case of *Runx1*). The promoters of some genes highly expressed in macrophages indeed transit through a poised state, which in many cases is already evident in ESCs (Figure S2D). However, transitions from the unmarked or repressed state are more frequent (Figure S2D, last row at the bottom).

A direct correlation between promoter state and gene expression is not seen with all differentially expressed genes (Figure 2B). Promoters of around one-third of differentially regulated genes are persistently in the active state despite highly dynamic gene expression (Figure 2B, highlighted). When this gene set was investigated for GO term enrichment, we found that most of their functions were "housekeeping" roles pertinent to regulation of cell cycle, protein catabolism, transport, and localization (Figures 2B and S2D; Table S4).

To link gene expression with the chromatin state of distal *cis*-regulatory elements, we associated them with their nearest genes and correlated changes in their chromatin state with the 31 gene expression patterns across the differentiation pathway (Figures S3A and S3B depict the actual expression patterns as heatmaps). This comparison demonstrates a strong correlation between the dynamics of the chromatin state of distal elements and gene expression, indicating that most of these elements function as enhancers. We noted that the number of distal elements that displayed a poised or repressed chromatin state was small. These results add to the increasing evidence that cell-type-specific spatiotemporal expression patterns are largely driven by distal regulatory elements (Lara-Astiaso et al., 2014) and in addition demonstrate that such elements are in either the active or inactive chromatin state.

## Chromatin Dynamics and TF Binding Determines the Differential Activity of *cis*-Regulatory Elements

We next addressed the question of which TFs were responsible for the cell-stage-specific opening of chromatin. We therefore determined dynamic DHS patterns during the differentiation time course and classified DHS patterns using a binary code with six digits (Figures 2C and 2D). We then performed pairwise comparisons between all our DHS patterns with each of the 32 TF ChIP-seq experiments (our own and publicly available data). Linking a set of regulatory genomic regions to annotated gene sets is sensitive to the varying sizes of the intergenic regions. We therefore used gene-set control analysis (GSCA) (Joshi et al., 2013), a tool designed to account for the differing sizes of such regions, to calculate pairwise correlation between TF ChIP-seq peak sets and expression gene sets, thus identifying all significant overlaps between TF binding events and DHS appearance. Figure 2D shows the most prevalent patterns of open chromatin over the six stages of development overlaid with the most significant TF binding events which in general, but not always, correlate with DHS presence. The most frequent DHS patterns are stage specific, over half of which involved DHS present only in macrophages (000001, 15,443 DHSs) and a quarter in ESC (100000, 7,302 DHSs). Notably, DHSs exclusively open in the HE (000100, 4,732 DHSs) are already primed by TF binding in HBs. The remaining patterns represent approximately 30% of all DHS whereby the majority of all patterns are continuous over at least two developmental stages. A common DHS pattern is 111111 (6,750 occurrences), the majority of which are CpG island promoters (Figure S4B) with a constitutively active chromatin state (Figure S4C). This class of DHS also contains the majority of binding events for C/EBP$\beta$ prior to the HP/MAC stages, suggesting a more widespread role of this transcription factor in development than previously thought.

Early binding of both LMO2 and TAL1 is highly significant in regulatory elements whose chromatin is first opened in HBs, HEs, or HPs, and include binding prior to the appearance of overt DHS sites, which is indicative of TF-mediated enhancer priming (see pattern DHS_000110). PU.1 binding shows significant overlap with DHS patterns in HPs but is also found at sites that only become hypersensitive in MACs. This suggests that PU.1 can prime MAC-specific regulatory regions already in early multipotent progenitors, lending weight to the finding that it is capable of opening chromatin (Garber et al., 2012; Natoli et al., 2011; Barozzi et al., 2014; Heinz et al., 2010, 2015).

We next correlated the statistical significance of the dynamics of distal DHS patterns with dynamic gene expression patterns (Figure S3B). This again demonstrates that the dynamics of chromatin accessibility at distal sites correlates well with the dynamics of gene expression (Figure S3B).

## The Complex Interplay between Chromatin Dynamics, Gene Expression, and TF Binding Events

Our next analysis determined the combinatorial pattern of TF-DNA interactions driving target gene expression at key stages of blood development. We therefore interrogated the 31

**Figure 2. Chromatin Programming during Progressive Lineage Commitment**

(A) Schematic diagram of the method used to coarse grain the 23-state chromatin model to four potential chromatin states.

(B) Clustering of promoters (1Kb up or downstream of the transcription start site, TSS) based on their chromatin state patterns (left) and the clustering of the expression pattern of genes that are constitutively expressed (right).

(C and D) Integration of DHS pattern and TF binding. (C) Methodology of the integrative analysis of chromatin dynamics and TF binding events. (D) TF binding events and the p values denoting the significance of overlap are depicted as gray-scale density plots, shown as dots. Integration of DHS (rows) and TF binding (columns) patterns across the six stages with the population size of each DHS pattern given on the right-hand side. For significance calculations only DHS patterns with a population size >100 were considered.

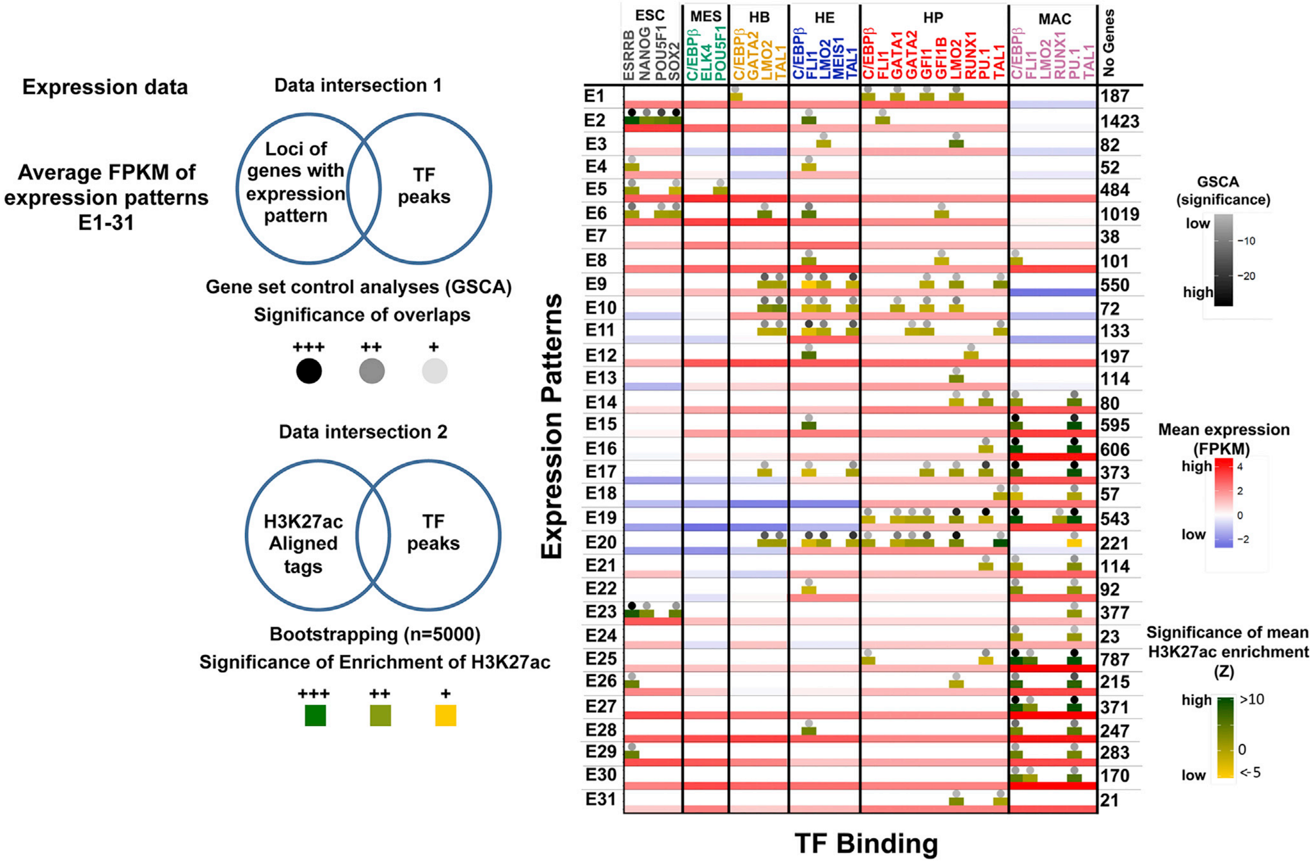


**Figure 3. Integration of Chromatin Dynamics, TF Binding Events, and Gene Expression during Hematopoietic Specification**

(Left) Flow diagram of data integration. The average expression values ($log_{10}$(FPKM)) of genes in expression patterns E1 to E31 were calculated for each developmental stage. The significance ($p < 0.0001$) of the overlap between the genes in each expression pattern and a given TF ChIP-seq peak set was obtained using gene-set control analysis. Z scores were obtained from the mean enrichment of H3K27ac in TF binding sites at these loci using bootstrapping. (Right) Average expression levels for each pattern (rows) are shown as a red-blue heatmap (see key), with columns for each cell type labeled at the top. The columns are further divided into TF ChIP-seq experiments, and the significant overlap between TF binding events and gene sets belonging to each expression pattern are depicted by gray-scale density plots shown as dots (see key). Significant overlap of these binding events and H3K27ac sites are also shown as a density plot depicted by yellow-green boxes (see key).

expression clusters (Figure 1F) to ascertain (1) whether expression patterns correlated with enriched binding of any of our examined 32 TF datasets to these genes, and (2) how such binding events correlated with histone H3K27 acetylation at this position. For visual inspection, the TF binding and histone acetylation data were then overlaid onto a heatmap summarizing gene expression for patterns E1–E31 (Figure 3). This analysis shows the overall correlation between dynamic transcription factor binding, histone acetylation, and gene expression. The genes expressed in patterns E17–E20 are associated with increased gene expression during hematopoiesis, all showing early low-level induction prior to high-level expression (Table S3). This induction is associated with significant binding of hematopoietic regulators, but not MEIS1. Highly significant early binding of LMO2/TAL1 in HB and FLI1/LMO2/TAL1 in HE occurs in genes expressed in patterns E9–E11. All three patterns are associated with binding of the repressor GFI1 in HP and with the repression of gene expression in macrophages. Patterns E9 and E11 involve upregulation of genes in the major HB-HE transition but then downregulation in HPs. Both sets of genes are enriched for functions relating to vasculogenesis, heart development, and cell adhesion (Figure S4A). Our results therefore highlight GFI1 as a candidate regulator involved in downregulating genes involved in non-hematopoietic cell fates following the HE to HP transition. This is consistent with data in the mouse that demonstrate a failure of EHT in the combined absence of GFI1 and GFIB in addition to the continued expression of endothelial genes (Lancrin et al., 2012; Lie-A-Ling et al., 2014; Thambyrajah et al., 2016). In summary, our analysis provides a highly informative integrated view of the dynamic relationships between gene expression, chromatin state, and TF binding.

## A Dynamic Core Gene Regulatory Network Driving Blood Development

To uncover the hierarchy of transcription factors driving blood specification forward, we generated gene regulatory network (GRN) representations connecting all 16 TFs analyzed by ChIP-seq, with separate representations for all six stages of development. To visualize different features, we illustrated multiple different data types within a single GRN representation at each

locus (Figure 4). Annotation for each of the six sequential developmental stages provided effective representation of the dynamics of cellular states, highlighted the chromatin features of the promoter of each gene locus, and indicated how interactions between a core set of key regulators drives developmental progression and terminal differentiation.

In ESCs all four pluripotency TFs participate in a highly connected core network circuit and already at this stage bind loci for hematopoietic TFs, including *Cebpb*, *Elk4*, *Gata2*, *Lmo2*, *Meis1*, *Runx1*, and *Tal1*, which display open or poised chromatin at their promoters, but also bind *Gfi1b*, *Gata1*, and *Spi1*, whose promoters are organized in closed-unmarked/repressed chromatin. As early as the HB stage, several hematopoietic regulator genes are upregulated, including *Tal1* and *Lmo2*, which exhibit autoregulation and co-regulate multiple genes. These include *Fli1* and *Meis1*, both of which are upregulated upon differentiation into HE. This stage is characterized by the involvement of LMO2, TAL1, and FLI1 (and in some cases MEIS1) in co-regulating genes for multiple hematopoietic TFs, revealing a densely connected GRN composed of potential feedback loops, which is likely to set the stage for the next step of hematopoietic commitment.

The HP stage shows highest expression for many of the key hematopoietic TFs, with binding events being complex and combinatorial. All ten TFs tested at this stage bind to *Gfi1*, *Gfi1b*, and *Runx1*, and nine out of ten bind to *Cebpb* and *Tal1* (the exceptions being RUNX1 and GFI1B, respectively). LMO2, TAL1, and to some extent FLI1 continue to co-bind and at this stage all target genes are shared with GFI1, consistent with the results shown in Figure 1D. FLI1 no longer binds to *Lmo2* or *Meis1*, both of which are strongly upregulated. While *Gata1* is upregulated by a combination of GFI1/LMO2/TAL1, *Gata2* is bound by C/EBPβ, TAL1, LMO2, FLI1, GATA1, GFI1, and GFI1B and is downregulated, uncovering a potential feedback mechanism regulating this TF within the network.

In macrophages, part of the HP-specific network is deconstructed with the further downregulation of early hematopoietic regulator genes such as *Gata2* and *Tal1* and a strong increase in the expression of PU.1 and C/EBPβ, which dominate global binding patterns. Within the GRN both TFs already share many target genes in HPs, including all genes encoding for experimental TFs (14 loci in total). Nine of these loci (*Cebpb*, *Elk4*, *Fli1*, *Gata2*, *Lmo2*, *Meis1*, *Runx1*, *Spi1*, *Tal1*) are also bound by FLI1 at this stage where *Lmo2* and *Tal1* are downregulated. Taken together, our datasets provide deep insights into the regulatory processes that control the dynamic rewiring of network connections during blood cell specification and differentiation. In the remaining part of this article we provide examples of how these data can be used to gain insights into the regulation of hematopoietic specification.

## Hierarchy Matters: TAL1/LMO2, but Not FLI1/GATA2, Can Reprogram Fibroblasts into Hematopoietic Cells

A number of recent publications reported a variety of TF combinations capable of generating blood cells via the reprogramming route (Batta et al., 2014; Elcheva et al., 2014; Pereira et al., 2013; Riddell et al., 2014; Sandler et al., 2014). We reasoned that the most likely factors capable of activating such a program would be those that (1) are expressed first during blood specification and (2) bind to a large number of genes required for blood cell development. Four tested factors fulfill these criteria, GATA2, TAL1, LMO2, and FLI1, with all of their respective genes being activated at the hemangioblast stage (Figure S1B). Figure S5A shows an extended transcriptional network highlighting TFs that have been used for reprogramming experiments demonstrating that most binding events within the hematopoietic transcriptional network involve TAL1/LMO2 which interact with one another, autoregulate, and bind *Fli1* and *Gata2*. Moreover, these factors synergize in driving hematopoietic development in zebrafish (Patterson et al., 2007). We therefore tested the hypothesis that TAL1/LMO2 overexpression would be sufficient to activate the hematopoietic developmental program in mouse embryonic fibroblasts (MEFs). To this end we transduced wild-type MEFs or MEFs carrying a doxycycline-inducible allele of *Tal1* with different combinations of expression vectors for the four factors (Figure 5A) and ensured that each construct was efficiently overexpressed (Figure S5B). We then scored the number of hematopoietic colonies (Figure 5B) and measured the activation of a blood-cell-specific gene expression program using RNA-seq (Figure 5C). These experiments show that (1) reprogramming generates cells with a gene expression profile that is highly correlated with that of HPs, (2) TAL1 and LMO2 are sufficient for reprogramming, (3) both are also necessary even in the presence of GATA2 and FLI1, and (4) GATA2 and FLI1 alone cannot reprogram efficiently even in the presence of either TAL1 or LMO2. Figure 5D shows that at least 13 important hematopoietic regulator genes are bound by the LMO2/TAL1 complex during the HB-HE transition, far exceeding those by any of the other tested factors. In addition, in HP, TAL1 and LMO2 cooperate to upregulate a battery of genes encoding downstream factors whose expression is upregulated, such as *Runx1*, *Gata1*, and *Spi1 (Pu.1)*. These experiments demonstrate that the integrated analysis of time course TF binding, chromatin accessibility/modification, and expression data enables to highlight those factors that are on top of the hierarchy of tissue specification and are involved in the activation of the majority of genes governing lineage-specific gene expression programs. We believe that this principle will be applicable in multiple developmental settings.

## Identification of Factors Driving Key Stages of Blood Specification: A Role for TEAD/YAP

We next used our dataset to identify transcriptional regulators of blood cell specification. We reasoned that cell-stage-specific regulators would leave their mark in the epigenome by occupying their respective binding motifs within cell-stage-specific DHS. To capture all relevant regulatory regions at the genome-wide scale, we used our chromatin accessibility data to perform a pairwise comparison of distal DHS from one cell type with all others as outlined in Figure 6A. For each set of DHSs unique to a given cell population, we determined relative enrichment for sequence motifs and performed a clustering analysis against cognate motifs of TFs expressed in these cells. Our analyses recovered the known role of specific factors in the relevant cell types. In ESCs the pluripotency factor motifs form a distinct cluster, whereas the RUNX motif is predominantly enriched at the HP stage (Figure 6B, blue arrow), where this TF is critically required (Chen et al., 2009; Lancrin et al., 2009). These results were

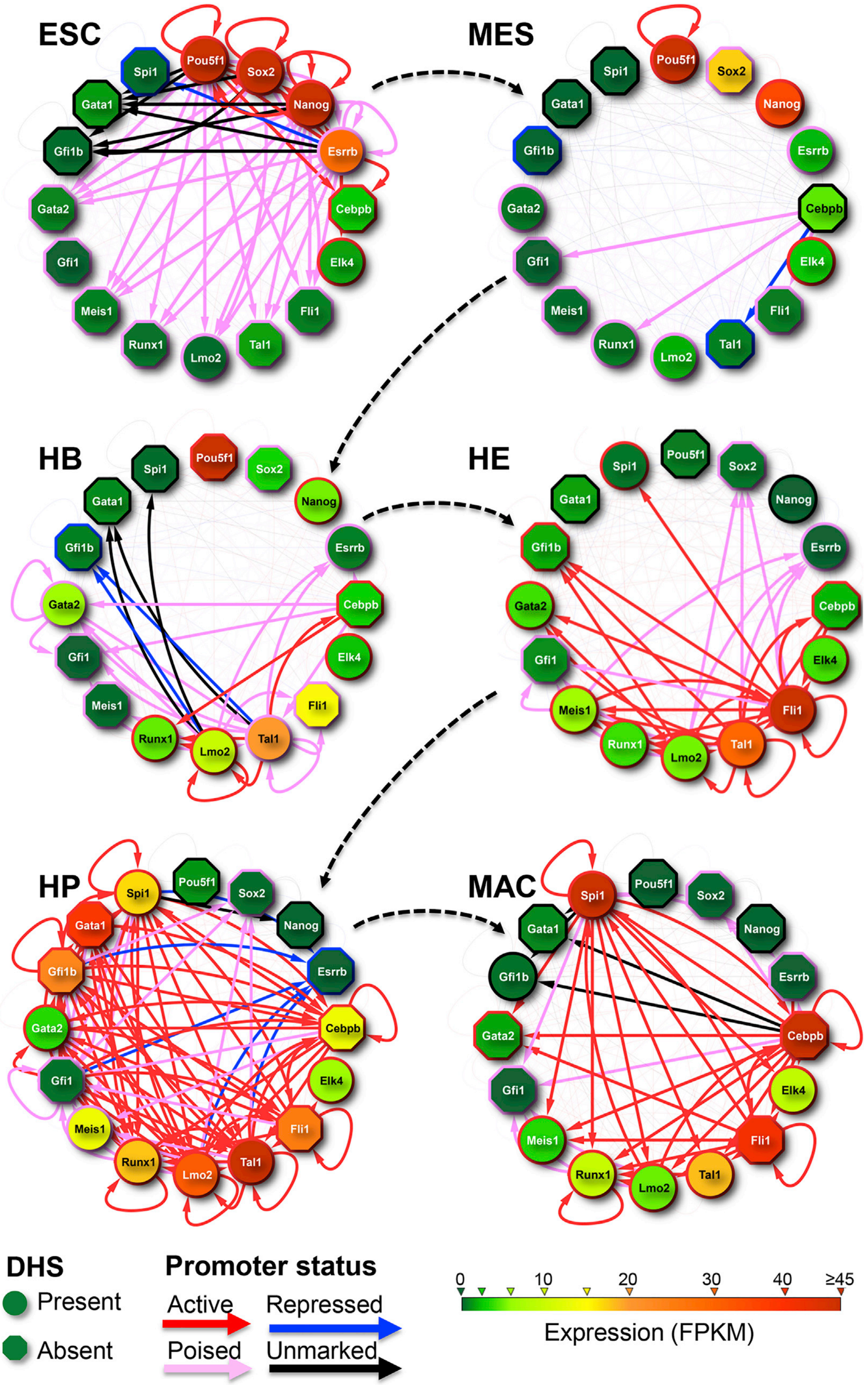


(legend on next page)

confirmed by analyzing enriched motifs within the TF ChIP-seq peaks (Figure S6A). Besides confirming the presence of the motifs for the assayed factors, we also discovered a significant cell-stage-specific enrichment of co-localizing motifs. An example is the significant enrichment of GATA motifs near the TAL1/LMO2 complex over several developmental stages, confirming the important role of this factor in forming a complex regulating hematopoietic genes (Wadman et al., 1997; Wilson et al., 2010). We also observed a strong enrichment of TCF7L1/2 motifs in TAL1/LMO2 peaks at the HB state, which are also predominant in the cell-specific DHS at this stage. TCF/LEF are mediators of Wnt signaling and have been shown to regulate hematopoietic specification (Sturgeon et al., 2014), raising the possibility that at this developmental stage elements binding the TAL1/LMO2 complex are WNT-signaling responsive.

The same analysis also uncovered enriched motifs for factors not yet linked with mammalian hematopoietic development, such as a significant enrichment for TEAD binding motifs early in hematopoietic development, specifically at the HB stage (Figure 6B, red arrow). TEAD motifs significantly co-localized with peaks for LMO2 and TAL1 in HBs and with TAL1/LMO2/FLI1 in the HE, but not in HPs (Figure 6C). TEAD transcriptional activity is controlled by the Hippo signaling network, which has emerged as a highly conserved pathway controlling cell proliferation, cell shape, organ size, and cell fate decisions in several differentiation pathways, including hematopoiesis in *Drosophila* (Dong et al., 2007; Ferguson and Martinez-Agosto, 2014; Milton et al., 2014). In mammals, when the Hippo pathway is activated, the MST and LATS kinases phosphorylate the transcriptional co-regulator YAP, which is then sequestered in the cytoplasm and consequently cannot form a complex with its nuclear DNA binding partner TEAD. When Hippo signaling is inactive, YAP interacts with TEAD factors in the nucleus to positively or negatively regulate Hippo signaling-responsive target genes (Yu and Guan, 2013).

To test whether TEAD factors are involved in regulating mammalian hematopoietic specification, we first looked for the presence of TEAD and YAP and any change in nuclear localization of YAP in mouse embryos. Figure 7A shows cross sections of developing blood islands from the yolk sac of embryonic day 7.5 (E7.5) mouse embryos stained with antibodies against TIE2 to identify endothelial cells, TEAD (upper panel) and YAP (lower panel). The images demonstrate a nuclear localization of YAP prior to the EHT but a cytoplasmic localization in hematopoietic cells. Staining of hematopoietic clusters emerging from the dorsal aorta from E10.5 mouse embryos (Figures S7A and S7B) also shows the absence of nuclear YAP in committed hematopoietic cells. The same is true for in vitro differentiated cells, where YAP is predominantly localized in the nucleus at the HB stage (Figures S6B and S6C) and then is localized in the cytoplasm in CD41$^{+}$ HP cells (Figure S6D), indicating a precise temporal regulation of TEAD activity during hematopoietic specification.

To test whether the interaction of YAP with TEAD factors is important for hematopoietic differentiation, we performed both in vitro and ex vivo experiments using verteporfin, which specifically inhibits TEAD-YAP complex formation, thus mimicking the Hippo pathway activation (Liu-Chittenden et al., 2012). Culturing ESC-derived embryoid bodies with the inhibitor blocked the emergence of CD41$^{+}$ HPs, but only when administered at early time points prior to day 5 of embryoid body (EB) culture when the emergence of blood cells occurs (Figures 7B and 7C). During normal HB development from mesodermal cells, the expression of *Fgf5* and *Bry* are downregulated concomitant with the upregulation of *Flk1* expression (Fehling et al., 2003). This process was abolished after treatment of isolated Bry$^{+}$ ME cells with verteporfin (Figure S6E). We also explanted FLK1$^{+}$/CD41$^{-}$ cells from E7.5 embryos and differentiated them into CD41$^{+}$ hematopoietic precursor cells on stromal cultures in the presence or absence of verteporfin (Figure S6F), and compared their response to cultured committed CD45$^{+}$ hematopoietic cells from E10.5 embryos (Figure S6G). Again, the addition of the inhibitor inhibited blood cell emergence and survival prior to, but not after hematopoietic specification. Moreover, genes encoding YAP and all TEAD factors were downregulated from the HP stage onward with expression essentially absent in macrophages (Table S2A). Together, these data suggest that (1) TEAD/YAP interaction is required at early stages of hematopoietic commitment and (2) YAP localizes outside of the nucleus after the EHT, suggesting that Hippo signaling is switched on in these cells.

## Identification of TEAD Target Genes

Having established an important role of TEAD and YAP interaction in hematopoietic specification, we performed ChIP-seq for TEAD4 from in vitro differentiated Flk1$^{+}$ hemangioblast cells to map its target genes. We mapped 5,234 TEAD4 binding regions (with manual validations shown in Figure S7C), including the *Tal1* locus (Figure 7D). Two-thirds of the binding sites occur in distal regions. Approximately 30% of all TEAD peaks (1,563) are in DHS that are active throughout all stages of differentiation (DHS pattern 111111, Figure 3, left panel, and Figures S4B and S4C). Most of these sites (1,342) are promoters, indicating that TEAD may fine-tune the expression of CG island promoters that predominate in such sites. TEAD binding sites in distal peaks are found predominantly in stage-specific DHS. A significant number of TEAD4 binding sites overlap with LMO2 and TAL1 binding sites in HB (Figure S7D), which is in concordance with the enrichment for TEAD motifs in early LMO2 and TAL1 binding events (Figures 6B and 6C).

Analysis of pathways enriched in TEAD4 bound genes (Table S8) revealed focal adhesion and Rap1 signaling as well as Wnt and transforming growth factor β signaling to be the top-scoring

**Figure 4. Dynamic Gene Regulatory Network Driving Hematopoietic Specification**

For each developmental stage the 16 TFs used in ChIP-seq experiments are shown as nodes in a GRN. The color of each node corresponds to the level of gene expression (see key). The chromatin accessibility at each promoter is shown as open/circular (DHS presence) or closed/octagonal (DHS absence), and the border color of the node corresponds to the promoter state according to the coarse-grain four-state model mentioned in Figure 2 (see key). Arrows indicate binding events of a TF (source) at loci encoding all TFs (target). The arrow color relates to the promoter state of the target TF encoding gene. No emanating arrow from a node indicates absence of ChIP data. For information about which ChIP experiments were conducted in which cell type, see Figure S1B. Note that we did not include C/EBPβ binding in the ES cell GRN, since the publicly available datasets did not contain the respective data.

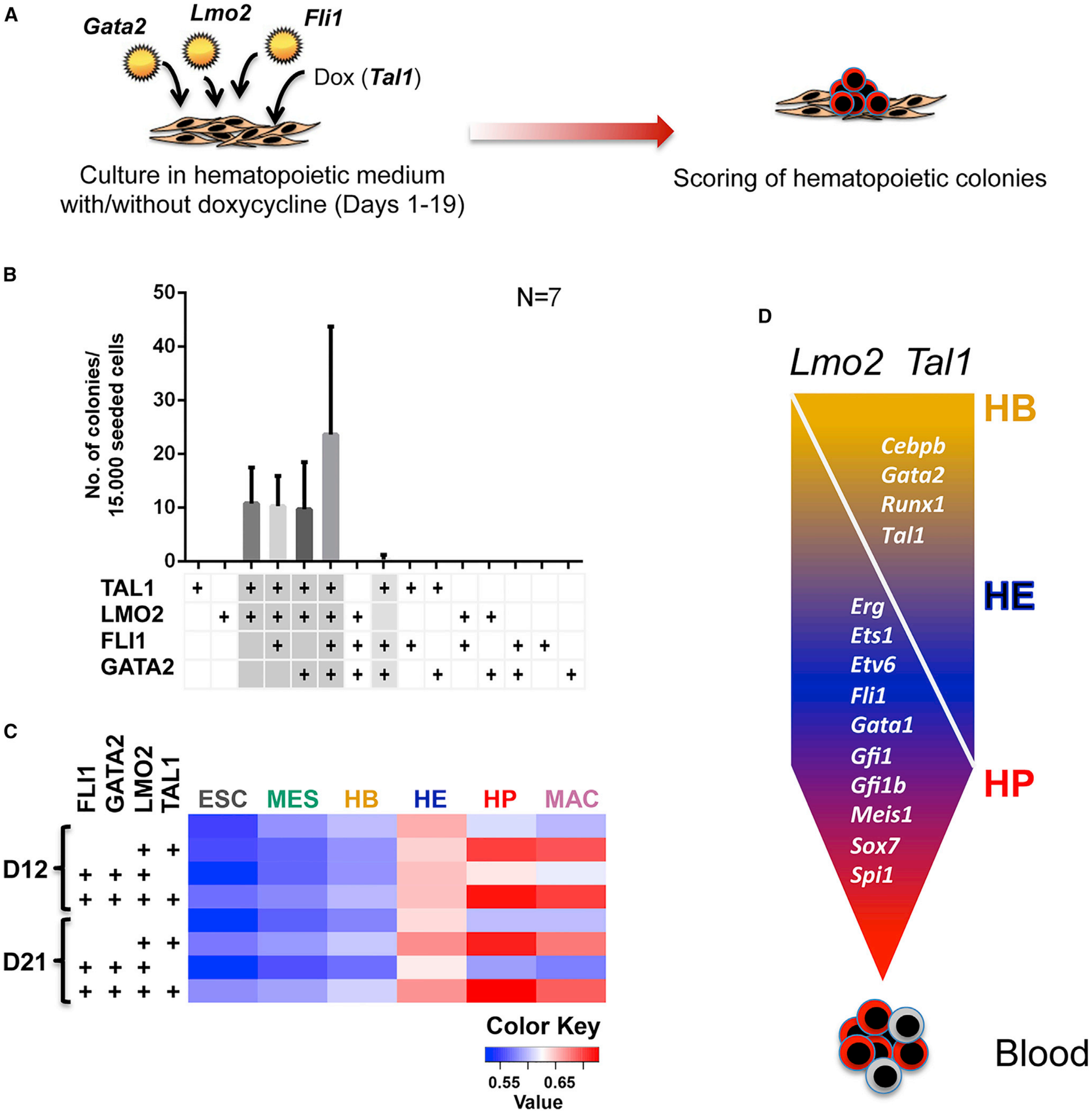


**Figure 5. Gene Regulatory Network Analysis Is Informative for Reprogramming Success**

(A) Schematic of reprogramming experiments.

(B) Bar chart showing the number of hematopoietic colonies generated from fibroblast cells after overexpression of different combinations of TAL1, LMO2, FLI1 and GATA2 as indicated in the table below. Grayed areas in this table highlight successful production of hematopoietic colonies. The number of hematopoietic colonies generated from fibroblast cells after overexpression of *Tal1*, *Lmo2*, *Fli1*, *Gata2*, and the indicated combinations of these four TF encoding genes. Data presented are mean ± SEM of individual experiments (n = 7).

(C) Correlation coefficient analyses of gene expression profiles generated by RNA-seq from hematopoietic cells generated by reprogramming from MEFs at day 12 and day 21 (D12 and D21) of the experiment with gene expression patterns generated from in vitro differentiated cells. The heatmap shows the correlation between expression data from each stage (columns) and each experiment (rows). All red/pink rectangles show correlation values that are significantly different from all blue rectangles (Z transform test, $p < 0.01$).

(D) Model depicting the hierarchy of transcriptional regulation during hematopoietic differentiation. Target TF genes are listed in the arrow, with those that are upregulated after binding events listed on the left and those that are downregulated listed on the right.

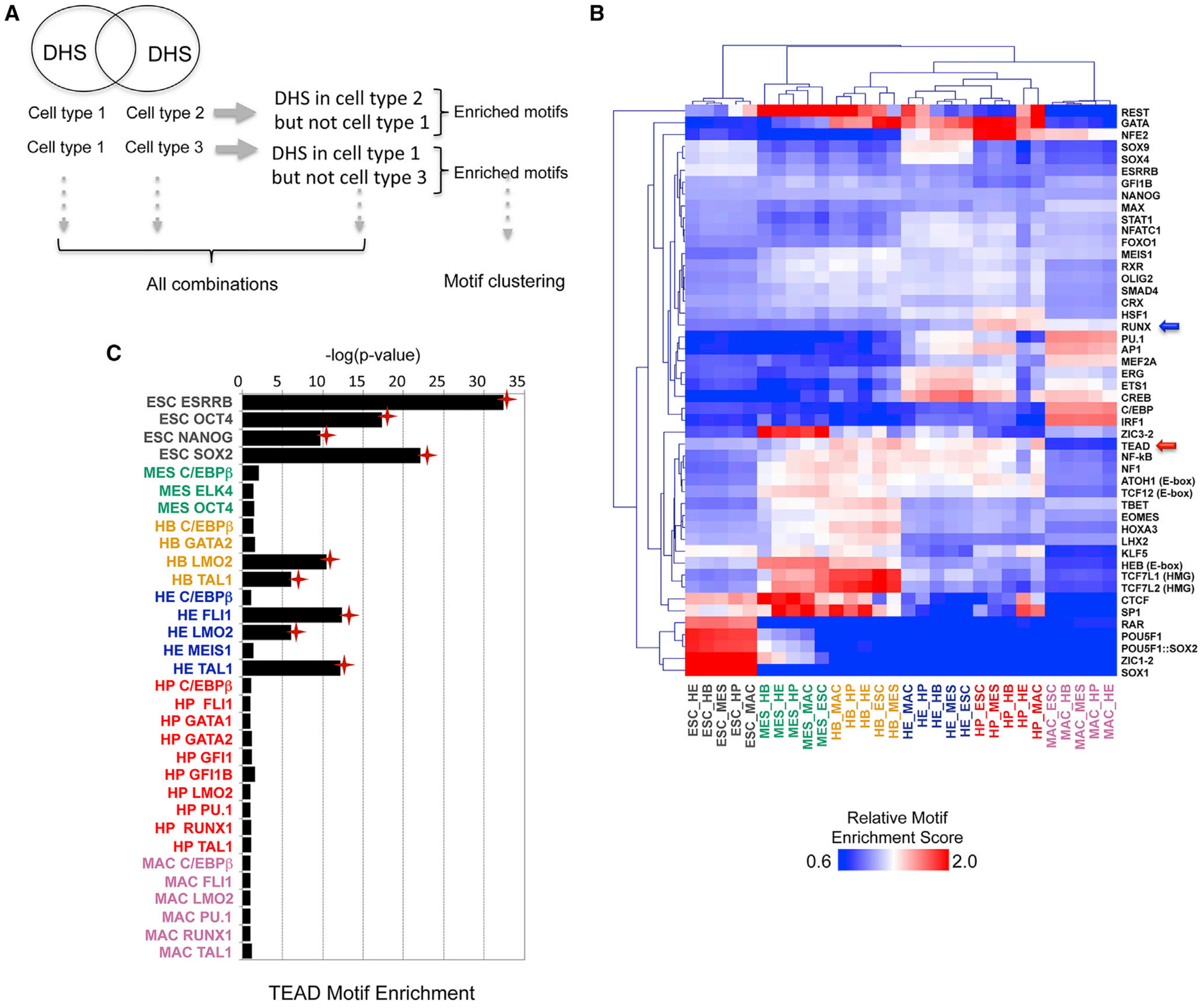


**Figure 6. Chromatin and Transcription Factor Binding Dynamics and Identification of Regulators**

(A) Schematic representation of the methodology for pairwise motif clustering at distal DHS (for used motifs see Table S6).

(B) Relative motif enrichment (RE) scores for the motifs in DHS unique to a given cell type compared with each of the other five cell types were clustered. RUNX and TEAD motifs are indicated by blue and red arrows, respectively. The significance of the RE scores were computed using the bootstrapping method (Table S7).

(C) Significance of co-localization of TEAD motifs in TF ChIP peaks. Red stars indicate significant co-localization.

pathways, all of which are known to influence hematopoietic and endothelial specification. In addition, a number of genes important for hematopoiesis are bound by TEAD, including the gene for the hematopoietic master regulator RUNX1 as well as *Kit*, which encodes a growth factor receptor crucial for the growth of hematopoietic precursor cells. In summary, our data indicate that TEAD factors target genes that regulate hematopoietic specification during the critical period leading to the EHT.

## DISCUSSION

### A Gene Regulatory Network Model for Hematopoietic Specification in the Embryo

The comprehensive experimental and computational studies of embryonic blood cell development reported here elucidate in fine detail how the interplay between cell-stage-specific TFs and the chromatin landscape drives differential gene expression during ontogeny. As indicated by the presence of a DHS, the majority of active cell-type-specific distal *cis*-regulatory elements correlate with significant binding of the measured TFs. Our data show a high complexity of developmental stage-specific TF assembly, with some factors binding first to be joined by others later in development, and with chromatin modifications following suit. Concurrent with the long-standing concept of developmental priming of distal *cis*-regulatory elements, a number of genes are bound by TFs in progenitor stages prior to high-level expression later in development. Recent experiments have shown that the early binding of TFs to distal elements of lineage-specific genes is required for the correct timing of gene activation and the repression of alternative fates (Lichtinger

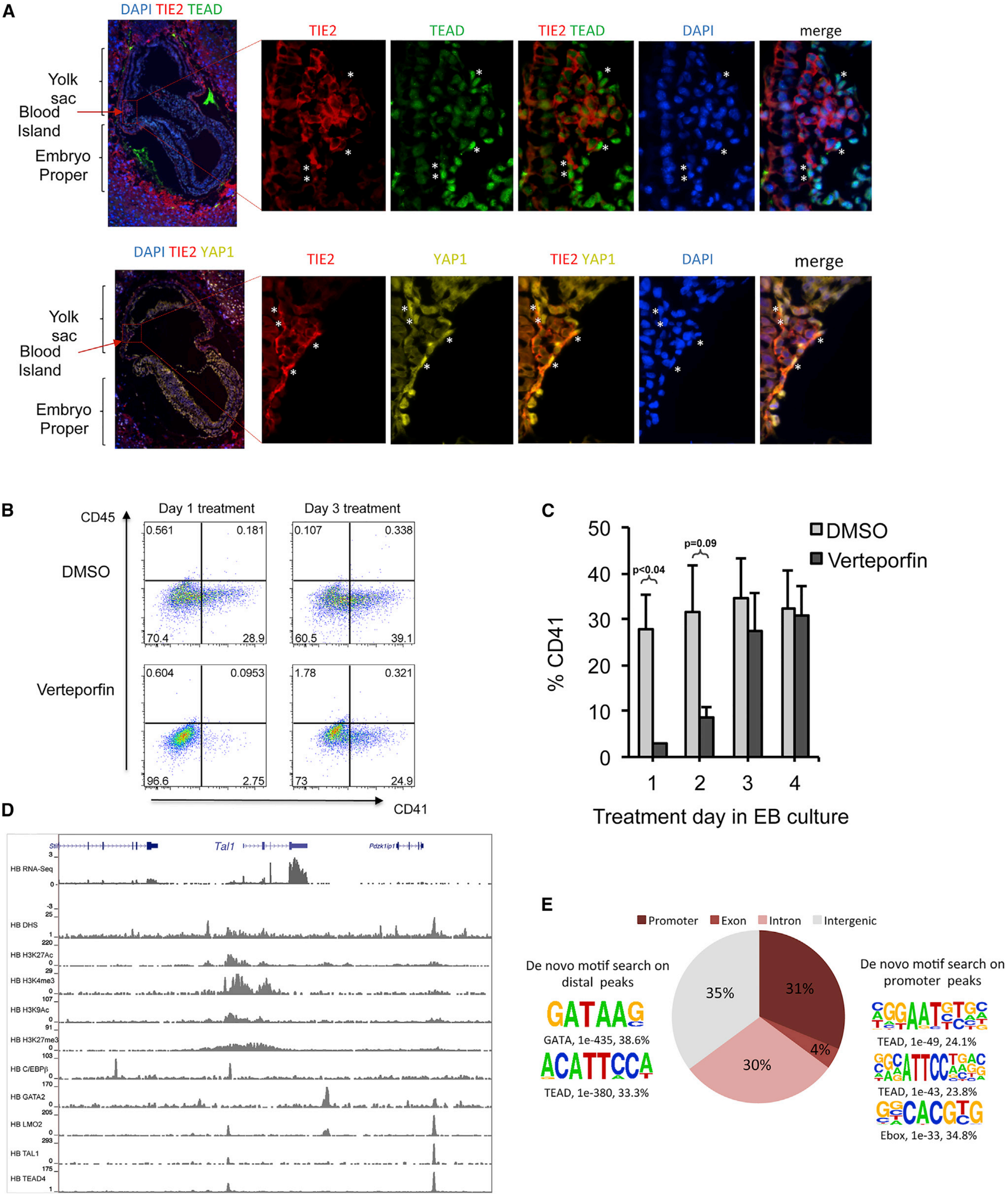


**Figure 7. A Role for TEAD Factors in Early Hematopoietic Specification**

(A) TEAD and YAP localize to the nucleus of a subset of TIE2$^{+}$ endothelium within yolk sac blood island of E7.5 embryos. E7.5 embryo sections were stained as indicated. Asterisks mark TIE2$^{+}$ cells on the outer edge of the developing blood island, which show nuclear localization for both TEAD and YAP. Cells within the blood island are maturing primitive erythrocytes and do not show nuclear localization for either TEAD or YAP.

(legend continued on next page)

et al., 2012; Org et al., 2015). Early transcription factor binding is therefore required to express genes at the correct developmental stage and at the appropriate level.

## Recapitulation of Early Developmental Processes during Reprogramming

The activation of a hematopoietic gene expression program in unrelated cells by external factors requires the alteration of the chromatin and transcriptional landscape and the activation of hematopoietic genes. Our dynamic GRN model provided essential clues about which factors (TAL1 and LMO2) were the most likely to succeed in this task and which ones would not. However, the actual molecular mechanism by which this occurs requires further investigation. E-Box motifs are widespread in the genome and are used by a variety of factors; we therefore suggest that the interaction with the bridging molecule LMO2 (Wadman et al., 1997) is crucial in bringing in additional factors. LMO2 forms part of a complex consisting of various E-box binding proteins as well as GATA, ETS, or RUNX1, and recruits LDB1 (Mylona et al., 2013). The latter mediates interactions between enhancer and promoter elements (Deng et al., 2012). The ETS factor ETV2 (ER71) is expressed in hemangioblasts and has been shown to be absolutely required for hematopoietic specification (Liu et al., 2015). This factor, together with GATA2 and TAL1, is capable of inducing enhanced hemangioblast formation during ES cell differentiation (Liu et al., 2013). However, TAL1 expression could rescue the hematopoietic defect in $ETV2^{-/-}$ cells, but FLI1 and GATA2 could not (Wareing et al., 2012), and when included in preliminary reprogramming experiments, ETV2 was incapable of generating hematopoietic colonies, on its own or in combination with either LMO2 or TAL1 (data not shown). These findings highlight the central role of the TAL1/LMO2 complex in driving a blood-cell-specific gene expression program and indicate that factors too far upstream in the hematopoietic specification hierarchy cannot substitute for hematopoietic factors.

## TEAD Factors Regulate Hematopoietic Specification in Mice

Our study provided an example of how our data resource can be used to advance our understanding of hematopoietic specification, in this case by using the differential analysis of enriched motifs in DHS. This type of analysis confirmed the role of known factors (such as RUNX1) but also identified a number of potential cell-stage-specific regulators of hematopoietic specification, leading to the identification of motifs for the Hippo-regulated TEAD TFs enriched specifically in MES, HB, and HE. Immunostaining analyses confirmed the presence of YAP in the nucleus of cells at stages prior to the EHT in both ESCs and embryo-derived cells, but not thereafter. Our functional studies show that the interaction of TEAD factors and the YAP co-factor in the nucleus at this early stage is strictly required for the formation of hematopoietic precursor cells from both sources.

How could TEAD factors impact on hematopoietic specification in the embryo? The analysis of their target genes in this study suggests that the TEAD/YAP complex may integrate the response to different types of signaling via interaction with genes encoding numerous signaling molecules whose balance controls hematopoietic specification such as bone morphogenetic proteins, NODAL and WNT, and fibroblast growth factor receptors (Pouget et al., 2014; Simoes et al., 2011). Moreover, TEAD/YAP interacts with genes of the Rap1 pathway, which controls cell adhesion dynamics and is essential for embryonic, but not adult hematopoiesis (Satyanarayana et al., 2010). Several recent studies have shown that the TEAD/YAP complex cooperates with tissue-specific TFs to either activate or repress gene expression. In human ESCs, TEAD/YAP interact with OCT4 to maintain the expression of pluripotency genes and represses mesendodermal genes, whereby repression can be overcome by the activation of BMP and Wnt signaling (Beyer et al., 2013; Estaras et al., 2015). The footprint of this interaction in the epigenome can be seen in our data as a co-localization of TEAD motifs with binding motifs for pluripotency factors in ESCs (Figures 6B and S6A). Our data show that in developing hematopoietic cells TEAD peaks co-localize with TAL1/LMO2 complexes, and SCL/TAL1 motif co-association continues to be significant up to the HE stage, but, again, not thereafter. This finding suggests that the factor complexes on such *cis*-regulatory elements may respond to Hippo signaling before, but not after the EHT. We have previously shown that genes associated with TAL1 and FLI1 binding in the HE are highly enriched for genes regulating cell shape and focal adhesion. During the EHT RUNX1 is strongly upregulated and relocates TAL1 and FLI1 to new binding sites after the EHT (Lichtinger et al., 2012), thus explaining the absence of co-localizing TEAD motifs in HPs. The expression of all TEADs as well as YAP is downregulated during terminal differentiation of HPs (Table S1A), thus uncoupling *cis*-element activity from TEAD-mediated signaling processes. Our analysis suggests the presence of a dynamic GRN of tissue-specific and lineage-specifying TFs that is intricately connected with signaling-responsive TFs such as TEAD or TCF7L1/2. Our data resource will enable numerous further functional and computational studies to examine the role of these different factors and thus gain insights into the molecular control mechanisms that underpin crucial steps in blood specification.

## EXPERIMENTAL PROCEDURES

A detailed description of all experiments can be found in Supplemental Experimental Procedures. All animal work was performed under regulation in accordance with the United Kingdom Animal Scientific Procedures Act (ASPA) 1986. Animal experiments were approved by the Animal Welfare and Ethics Review Body (AWERB) of the Cancer Research UK Manchester Institute.

(B and C) TEAD activity is required during the early phase of hematopoietic commitment. The TEAD-YAP inhibitor verteporfin (9.6 μM) or DMSO vehicle was added on day 1, 2, 3, or 4 of EB culture. Day-1 EB corresponds to mesoderm (MES) commitment, day 2–3 to hemangioblast (HB), commitment and day 4–5 to HE and HP specification. The frequency of $CD41^+$ hematopoietic cells was determined on day 7. (B) Representative FACS plots. (C) Quantification of the percentage of $CD41^+$ cells from $n \geq 3$ independent experiments. Data presented are mean ± SEM, paired t test.

(D) Genome browser screenshot showing TEAD4 binding to the *Tal1* locus in HB together with other chromatin and binding features.

(E) Genomic distribution of TEAD4 peaks together with TF binding motifs enriched in distal (left) and proximal (right) peaks.

### Isolation of Cell Populations

A mouse ES cell line carrying a brachyury (Bry) GFP$^+$ reporter gene was cultured as described by Sroczynska et al. (2009). GFP and cell surface marker staining were used to identify each cell population by cell sorting. MES cells were (Bry$^+$Flk1$^-$), HB cells were (Bry$^+$/Flk1$^+$), HE cells were Tie2$^+$/cKit$^+$/CD41$^-$), and HPs were CD41$^+$. Macrophages were isolated by differentiation of CD41$^+$ cells to CD11b-expressing cells.

### Chromatin Immunoprecipitation followed by Next-Generation Sequencing

For each stage of differentiation cells were sorted, crosslinked, and stored either as frozen cells (histone modification ChIP) or nuclei (TF ChIP) for subsequent ChIP assays, performed as described previously (Lichtinger et al., 2012; Wilson et al., 2009). A full list of antibody sources is given in Supplemental Experimental Procedures.

DNaseI and ChIP samples were amplified and sequenced according to the manufacturer's instructions. Some samples (MES, HB and HP H3K4me3, HP H3K27me3, and HP H3K27ac) were processed using an ABI SOLiD 4 sequencer, and subsequently either the Illumina 2G Genome Analyzer or the Hi-Seq 2000 were used.

### RNA Sequencing

RNA preparation and sequencing were performed as described previously (Lie-A-Ling et al., 2014).

### DNaseI Sequencing

One to three million freshly sorted cells were digested with DNaseI enzyme as described in detail previously (Ptasinska et al., 2014) and size selected for 50- to 300-bp fragments.

### Reprogramming Experiments

E14.5 murine wild-type fibroblasts (MEFs) or from iTal1-2A-GFP transgenic mice carrying rtTA and TRE-TAL1 cassettes allowing inducible expression of TAL1 upon addition of doxycycline were prepared as described previously (Sroczynska et al., 2009). Reprogramming experiments were carried out with either wild-type (n = 5) or iTal1-2A-GFP (n = 2) MEFs as described by Batta et al. (2014).

### Inhibition of TEAD/YAP Interaction In Vitro and Ex Vivo

Verteporfin was added to day-1, -2, -3, or -4 EB cultures at a final concentration of 9.6 μM, and EB-derived cells were stained with CD41-PE and CD45-brilliant violet 421 (Becton Dickinson) and analyzed by flow cytometry. BRY$^+$/FLK1$^-$ mesoderm-enriched population was cultured for 48 hr with or without verteporfin. Expression of *Fgf5*, *Bry*, and *Flk1* was measured by RT-PCR. FLK1$^+$/CD41$^-$ HE-enriched cells were sorted from E7.5 embryos by fluorescence-activated cell sorting (FACS) and cultured for 4 days on irradiated OP9 in HE media, and hematopoietic differentiation of HE cells was measured by staining with a CD41-PE antibody. CD45$^+$ committed hematopoietic cells were sorted from E10.5 embryos and cultured on OP9 with verteporfin or DMSO. Growth and survival of hematopoietic cells was assessed by FACS after staining with a CD45-fluorescein isothiocyanate antibody.

### Immunostaining of Embryos

E7.5 embryos were fixed in 4% paraformaldehyde, mounted, and stained with rabbit Pan-TEAD (D3F7L, New England Biolabs) (1:100), rabbit YAP (D8HIX XP, New England Biolabs) (1:100), or purified anti-mouse Tie2 (Tek-CD202B, 14-5987-85, eBioscience) (1:100). Secondary antibodies were Alexa Fluor 488 goat anti-rat immunoglobulin G (IgG) (A11006, Life Technologies) and Alexa Fluor 647 F(ab′)2 fragment of goat anti-rabbit IgG (H + L) (A21246, Life Technologies).

### Data Analysis

A detailed description of all bioinformatics methods can be found in Supplemental Experimental Procedures.

## ACCESSION NUMBERS

All data and associated analyses are provided in our web server http://www.bioinformatics.leeds.ac.uk/labpages/hematopoiesis/. The tag densities for all our experiments have been integrated into the genome browser in http://www.haemopoiesis.leeds.ac.uk. Data can also be found at: http://codex.stemcells.cam.ac.uk/. The raw fastq, aligned bam, peak, and tag density files are available from the NCBI Gene Expression Omnibus portal (GEO: GSE69101).

## SUPPLEMENTAL INFORMATION

Supplemental Information includes Supplemental Experimental Procedures, seven figures, and eight tables and can be found with this article online at http://dx.doi.org/10.1016/j.devcel.2016.01.024.

## AUTHOR CONTRIBUTIONS

D.K.G., N.O., M.L., M.L.A.L., A.J.L., M.F., K.B., M.C., K.W., J.G., and M.H. performed experiments; M.S.V., H.R., S.A.A., and P.C. analyzed data. The senior authors (D.R.W., G.L., V.K., B.G., and C.B.) jointly obtained funding, conceived, and directed the study, and, together with D.K.G., wrote the paper. Within the consortium, D.R.W. directed data analysis/integration and creation of the website; G.L. and V.K. oversaw cell differentiation and purification, RNA-seq data generation, reprogramming and the in vivo and inhibitor studies; B.G. oversaw the transcription factor ChIP data generation and further data analysis; and C.B. was the project coordinator and oversaw chromatin data generation as well as further data analysis and integration.

## ACKNOWLEDGMENTS

This work was funded by a Longer Larger (LoLa) consortium grant from the Biotechnology and Biological Sciences Research Council, UK, to the senior authors and the corresponding author, computing infrastructure grants from the Wellcome Trust and National Institute for Health Research to B.G., grants from Cancer Research UK to G.L. and V.K., and funding from the Bloodwise charity to C.B.

Received: May 28, 2015
Revised: December 4, 2015
Accepted: January 26, 2016
Published: February 25, 2016

## REFERENCES

Barozzi, I., Simonatto, M., Bonifacio, S., Yang, L., Rohs, R., Ghisletti, S., and Natoli, G. (2014). Coregulation of transcription factor binding and nucleosome occupancy through DNA features of mammalian enhancers. Mol. Cell *54*, 844–857.

Batta, K., Florkowska, M., Kouskoff, V., and Lacaud, G. (2014). Direct reprogramming of murine fibroblasts to hematopoietic progenitor cells. Cell Rep. *9*, 1871–1884.

Beyer, T.A., Weiss, A., Khomchuk, Y., Huang, K., Ogunjimi, A.A., Varelas, X., and Wrana, J.L. (2013). Switch enhancers interpret TGF-beta and Hippo signaling to control cell fate in human embryonic stem cells. Cell Rep. *5*, 1611–1624.

Bonifer, C., Hoogenkamp, M., Krysinska, H., and Tagoh, H. (2008). How transcription factors program chromatin—lessons from studies of the regulation of myeloid-specific genes. Semin. Immunol. *20*, 257–263.

Chen, X., Xu, H., Yuan, P., Fang, F., Huss, M., Vega, V.B., Wong, E., Orlov, Y.L., Zhang, W., Jiang, J., et al. (2008). Integration of external signaling pathways with the core transcriptional network in embryonic stem cells. Cell *133*, 1106–1117.

Chen, M.J., Yokomizo, T., Zeigler, B.M., Dzierzak, E., and Speck, N.A. (2009). Runx1 is required for the endothelial to haematopoietic cell transition but not thereafter. Nature *457*, 887–891.

Cockerill, P.N. (2011). Structure and function of active chromatin and DNase I hypersensitive sites. FEBS J. *278*, 2182–2210.

Deng, W., Lee, J., Wang, H., Miller, J., Reik, A., Gregory, P.D., Dean, A., and Blobel, G.A. (2012). Controlling long-range genomic interactions at a native locus by targeted tethering of a looping factor. Cell *149*, 1233–1244.

Dong, J., Feldmann, G., Huang, J., Wu, S., Zhang, N., Comerford, S.A., Gayyed, M.F., Anders, R.A., Maitra, A., and Pan, D. (2007). Elucidation of a universal size-control mechanism in *Drosophila* and mammals. Cell *130*, 1120–1133.

Elcheva, I., Brok-Volchanskaya, V., Kumar, A., Liu, P., Lee, J.-H., Tong, L., Vodyanik, M., Swanson, S., Stewart, R., Kyba, M., et al. (2014). Direct induction of haematoendothelial programs in human pluripotent stem cells by transcriptional regulators. Nat. Commun. *5*, 4372.

Ernst, J., and Kellis, M. (2012). ChromHMM: automating chromatin-state discovery and characterization. Nat. Methods *9*, 215–216.

Estaras, C., Benner, C., and Jones, K.A. (2015). SMADs and YAP compete to control elongation of beta-catenin:LEF-1-recruited RNAPII during hESC differentiation. Mol. Cell *58*, 780–793.

Fehling, H.J., Lacaud, G., Kubo, A., Kennedy, M., Robertson, S., Keller, G., and Kouskoff, V. (2003). Tracking mesoderm induction and its specification to the hemangioblast during embryonic stem cell differentiation. Development *130*, 4217–4227.

Ferguson, G.B., and Martinez-Agosto, J.A. (2014). Yorkie and Scalloped signaling regulates Notch-dependent lineage specification during *Drosophila* hematopoiesis. Curr. Biol. *24*, 2665–2672.

Garber, M., Yosef, N., Goren, A., Raychowdhury, R., Thielke, A., Guttman, M., Robinson, J., Minie, B., Chevrier, N., Itzhaki, Z., et al. (2012). A high-throughput chromatin immunoprecipitation approach reveals principles of dynamic gene regulation in mammals. Mol. Cell *47*, 810–822.

Heinz, S., Benner, C., Spann, N., Bertolino, E., Lin, Y.C., Laslo, P., Cheng, J.X., Murre, C., Singh, H., and Glass, C.K. (2010). Simple combinations of lineage-determining transcription factors prime cis-regulatory elements required for macrophage and B cell identities. Mol. Cell *38*, 576–589.

Heinz, S., Romanoski, C.E., Benner, C., and Glass, C.K. (2015). The selection and function of cell type-specific enhancers. Nat. Rev. Mol. Cel. Biol. *16*, 144–154.

Hoogenkamp, M., Lichtinger, M., Krysinska, H., Lancrin, C., Clarke, D., Williamson, A., Mazzarella, L., Ingram, R., Jorgensen, H., Fisher, A., et al. (2009). Early chromatin unfolding by RUNX1: a molecular explanation for differential requirements during specification versus maintenance of the hematopoietic gene expression program. Blood *114*, 299–309.

Joshi, A., Hannah, R., Diamanti, E., and Gottgens, B. (2013). Gene set control analysis predicts hematopoietic control mechanisms from genome-wide transcription factor binding data. Exp. Hematol. *41*, 354–366.e314.

Krysinska, H., Hoogenkamp, M., Ingram, R., Wilson, N., Tagoh, H., Laslo, P., Singh, H., and Bonifer, C. (2007). A two-step, PU.1-dependent mechanism for developmentally regulated chromatin remodeling and transcription of the c-fms gene. Mol. Cell Biol. *27*, 878–887.

Lancrin, C., Sroczynska, P., Stephenson, C., Allen, T., Kouskoff, V., and Lacaud, G. (2009). The haemangioblast generates haematopoietic cells through a haemogenic endothelium stage. Nature *457*, 892–895.

Lancrin, C., Sroczynska, P., Serrano, A.G., Gandillet, A., Ferreras, C., Kouskoff, V., and Lacaud, G. (2010). Blood cell generation from the hemangioblast. J. Mol. Med. (Berl) *88*, 167–172.

Lancrin, C., Mazan, M., Stefanska, M., Patel, R., Lichtinger, M., Costa, G., Vargel, O., Wilson, N.K., Moroy, T., Bonifer, C., et al. (2012). GFI1 and GFI1B control the loss of endothelial identity of hemogenic endothelium during hematopoietic commitment. Blood *120*, 314–322.

Lara-Astiaso, D., Weiner, A., Lorenzo-Vivas, E., Zaretsky, I., Jaitin, D.A., David, E., Keren-Shaul, H., Mildner, A., Winter, D., Jung, S., et al. (2014). Immunogenetics. Chromatin state dynamics during blood formation. Science *345*, 943–949.

Leddin, M., Perrod, C., Hoogenkamp, M., Ghani, S., Assi, S., Heinz, S., Wilson, N.K., Follows, G., Schonheit, J., Vockentanz, L., et al. (2011). Two distinct auto-regulatory loops operate at the PU.1 locus in B cells and myeloid cells. Blood *117*, 2827–2838.

Lichtinger, M., Ingram, R., Hannah, R., Muller, D., Clarke, D., Assi, S.A., Lie-A-Ling, M., Noailles, L., Vijayabaskar, M.S., Wu, M., et al. (2012). RUNX1 reshapes the epigenetic landscape at the onset of haematopoiesis. EMBO J. *31*, 4318–4333.

Lie-A-Ling, M., Marinopoulou, E., Li, Y., Patel, R., Stefanska, M., Bonifer, C., Miller, C., Kouskoff, V., and Lacaud, G. (2014). RUNX1 positively regulates a cell adhesion and migration program in murine hemogenic endothelium prior to blood emergence. Blood *124*, e11–20.

Liu, F., Bhang, S.H., Arentson, E., Sawada, A., Kim, C.K., Kang, I., Yu, J., Sakurai, N., Kim, S.H., Yoo, J.J.W., et al. (2013). Enhanced hemangioblast generation and improved vascular repair and regeneration from embryonic stem cells by defined transcription factors. Stem Cell Rep. *1*, 166–182.

Liu, F., Li, D., Yu, Y.Y., Kang, I., Cha, M.J., Kim, J.Y., Park, C., Watson, D.K., Wang, T., and Choi, K. (2015). Induction of hematopoietic and endothelial cell program orchestrated by ETS transcription factor ER71/ETV2. EMBO Rep. *16*, 654–669.

Liu-Chittenden, Y., Huang, B., Shim, J.S., Chen, Q., Lee, S.-J., Anders, R.A., Liu, J.O., and Pan, D. (2012). Genetic and pharmacological disruption of the TEAD-YAP complex suppresses the oncogenic activity of YAP. Genes Dev. *26*, 1300–1305.

Medvinsky, A., Rybtsov, S., and Taoudi, S. (2011). Embryonic origin of the adult hematopoietic system: advances and questions. Development *138*, 1017–1031.

Milton, C.C., Grusche, F.A., Degoutin, J.L., Yu, E., Dai, Q., Lai, E.C., and Harvey, K.F. (2014). The Hippo pathway regulates hematopoiesis in *Drosophila melanogaster*. Curr. Biol. *24*, 2673–2680.

Mylona, A., Andrieu-Soler, C., Thongjuea, S., Martella, A., Soler, E., Jorna, R., Hou, J., Kockx, C., van Ijcken, W., Lenhard, B., et al. (2013). Genome-wide analysis shows that Ldb1 controls essential hematopoietic genes/pathways in mouse early development and reveals novel players in hematopoiesis. Blood *121*, 2902–2913.

Natoli, G., Ghisletti, S., and Barozzi, I. (2011). The genomic landscapes of inflammation. Genes Dev. *25*, 101–106.

Org, T., Duan, D., Ferrari, R., Montel-Hagen, A., Van Handel, B., Kerenyi, M.A., Sasidharan, R., Rubbi, L., Fujiwara, Y., Pellegrini, M., et al. (2015). Scl binds to primed enhancers in mesoderm to regulate hematopoietic and cardiac fate divergence. EMBO J. *34*, 759–777.

Patterson, L.J., Gering, M., Eckfeldt, C.E., Green, A.R., Verfaillie, C.M., Ekker, S.C., and Patient, R. (2007). The transcription factors Scl and Lmo2 act together during development of the hemangioblast in zebrafish. Blood *109*, 2389–2398.

Pereira, C.-F., Chang, B., Qiu, J., Niu, X., Papatsenko, D., Hendry, C.E., Clark, N.R., Nomura-Kitabayashi, A., Kovacic, J.C., Ma'ayan, A., et al. (2013). Induction of a hemogenic program in mouse fibroblasts. Cell Stem Cell *13*, 205–218.

Pouget, C., Peterkin, T., Simoes, F.C., Lee, Y., Traver, D., and Patient, R. (2014). FGF signalling restricts haematopoietic stem cell specification via modulation of the BMP pathway. Nat. Commun. *5*, 5588.

Ptasinska, A., Assi, S.A., Martinez-Soria, N., Imperato, M.R., Piper, J., Cauchy, P., Pickin, A., James, S.R., Hoogenkamp, M., Williamson, D., et al. (2014). Identification of a dynamic core transcriptional network in t(8;21) AML that regulates differentiation block and self-renewal. Cell Rep. *8*, 1974–1988.

Riddell, J., Gazit, R., Garrison, B.S., Guo, G., Saadatpour, A., Mandal, P.K., Ebina, W., Volchkov, P., Yuan, G.-C., Orkin, S.H., et al. (2014). Reprogramming committed murine blood cells to induced hematopoietic stem cells with defined factors. Cell *157*, 549–564.

Sandler, V.M., Lis, R., Liu, Y., Kedem, A., James, D., Elemento, O., Butler, J.M., Scandura, J.M., and Rafii, S. (2014). Reprogramming human endothelial cells to haematopoietic cells requires vascular induction. Nature *511*, 312–318.

Satyanarayana, A., Gudmundsson, K.O., Chen, X., Coppola, V., Tessarollo, L., Keller, J.R., and Hou, S.X. (2010). RapGEF2 is essential for embryonic hematopoiesis but dispensable for adult hematopoiesis. Blood *116*, 2921–2931.

Shivdasani, R.A., Mayer, E.L., and Orkin, S.H. (1995). Absence of blood formation in mice lacking the T-cell leukaemia oncoprotein tal-1/SCL. Nature *373*, 432–434.

Simoes, F.C., Peterkin, T., and Patient, R. (2011). Fgf differentially controls cross-antagonism between cardiac and haemangioblast regulators. Development *138*, 3235–3245.

Sroczynska, P., Lancrin, C., Pearson, S., Kouskoff, V., and Lacaud, G. (2009). In vitro differentiation of mouse embryonic stem cells as a model of early hematopoietic development. Methods Mol. Biol. *538*, 317–334.

Sturgeon, C.M., Ditadi, A., Awong, G., Kennedy, M., and Keller, G. (2014). Wnt signaling controls the specification of definitive and primitive hematopoiesis from human pluripotent stem cells. Nat. Biotechnol. *32*, 554–561.

Tanaka, Y., Joshi, A., Wilson, N.K., Kinston, S., Nishikawa, S., and Gottgens, B. (2012). The transcriptional programme controlled by Runx1 during early embryonic blood development. Dev. Biol. *366*, 404–419.

Thambyrajah, R., Mazan, M., Patel, R., Moignard, V., Stefanska, M., Marinopoulou, E., Li, Y., Lancrin, C., Clapes, T., Möröy, T., et al. (2016). GFI1 proteins orchestrate the emergence of haematopoietic stem cells through recruitment of LSD1. Nat. Cell Biol. *18*, 21–32.

Tsankov, A.M., Gu, H., Akopian, V., Ziller, M.J., Donaghey, J., Amit, I., Gnirke, A., and Meissner, A. (2015). Transcription factor binding dynamics during human ES cell differentiation. Nature *518*, 344–349.

Van Nostrand, E.L., and Kim, S.K. (2011). Seeing elegance in gene regulatory networks of the worm. Curr. Opin. Genet. Dev. *21*, 776–786.

Wadman, I.A., Osada, H., Grutz, G.G., Agulnick, A.D., Westphal, H., Forster, A., and Rabbitts, T.H. (1997). The LIM-only protein Lmo2 is a bridging molecule assembling an erythroid, DNA-binding complex which includes the TAL1, E47, GATA-1 and Ldb1/NLI proteins. EMBO J. *16*, 3145–3157.

Wamstad, J.A., Alexander, J.M., Truty, R.M., Shrikumar, A., Li, F., Eilertson, K.E., Ding, H., Wylie, J.N., Pico, A.R., Capra, J.A., et al. (2012). Dynamic and coordinated epigenetic regulation of developmental transitions in the cardiac lineage. Cell *151*, 206–220.

Wang, A., Yue, F., Li, Y., Xie, R., Harper, T., Patel, N.A., Muth, K., Palmer, J., Qiu, Y., Wang, J., et al. (2015). Epigenetic priming of enhancers predicts developmental competence of hESC-derived endodermal lineage intermediates. Cell Stem Cell *16*, 386–399.

Wareing, S., Mazan, A., Pearson, S., Gottgens, B., Lacaud, G., and Kouskoff, V. (2012). The Flk1-Cre-mediated deletion of ETV2 defines its narrow temporal requirement during embryonic hematopoietic development. Stem Cells *30*, 1521–1531.

Whyte, W.A., Orlando, D.A., Hnisz, D., Abraham, B.J., Lin, C.Y., Kagey, M.H., Rahl, P.B., Lee, T.I., and Young, R.A. (2013). Master transcription factors and mediator establish super-enhancers at key cell identity genes. Cell *153*, 307–319.

Wilson, N.K., Miranda-Saavedra, D., Kinston, S., Bonadies, N., Foster, S.D., Calero-Nieto, F., Dawson, M.A., Donaldson, I.J., Dumon, S., Frampton, J., et al. (2009). The transcriptional program controlled by the stem cell leukemia gene Scl/Tal1 during early embryonic hematopoietic development. Blood *113*, 5456–5465.

Wilson, N.K., Foster, S.D., Wang, X., Knezevic, K., Schutte, J., Kaimakis, P., Chilarska, P.M., Kinston, S., Ouwehand, W.H., Dzierzak, E., et al. (2010). Combinatorial transcriptional control in blood stem/progenitor cells: genome-wide analysis of ten major transcriptional regulators. Cell Stem Cell *7*, 532–544.

Yu, F.-X., and Guan, K.-L. (2013). The Hippo pathway: regulators and regulations. Genes Dev. *27*, 355–371.

Zinzen, R.P., Girardot, C., Gagneur, J., Braun, M., and Furlong, E.E. (2009). Combinatorial binding predicts spatio-temporal cis-regulatory activity. Nature *462*, 65–70.

Developmental Cell, Volume *36*

## Supplemental Information

# Dynamic Gene Regulatory Networks Drive Hematopoietic Specification and Differentiation

**Debbie K. Goode, Nadine Obier, M.S. Vijayabaskar, Michael Lie-A-Ling, Andrew J. Lilly, Rebecca Hannah, Monika Lichtinger, Kiran Batta, Magdalena Florkowska, Rahima Patel, Mairi Challinor, Kirstie Wallace, Jane Gilmour, Salam A. Assi, Pierre Cauchy, Maarten Hoogenkamp, David R. Westhead, Georges Lacaud, Valerie Kouskoff, Berthold Göttgens, and Constanze Bonifer**

# Goode et al., Supplemental Materials

## 1. SUPPLEMENTAL FIGURES, FIGURE LEGENDS AND TABLES

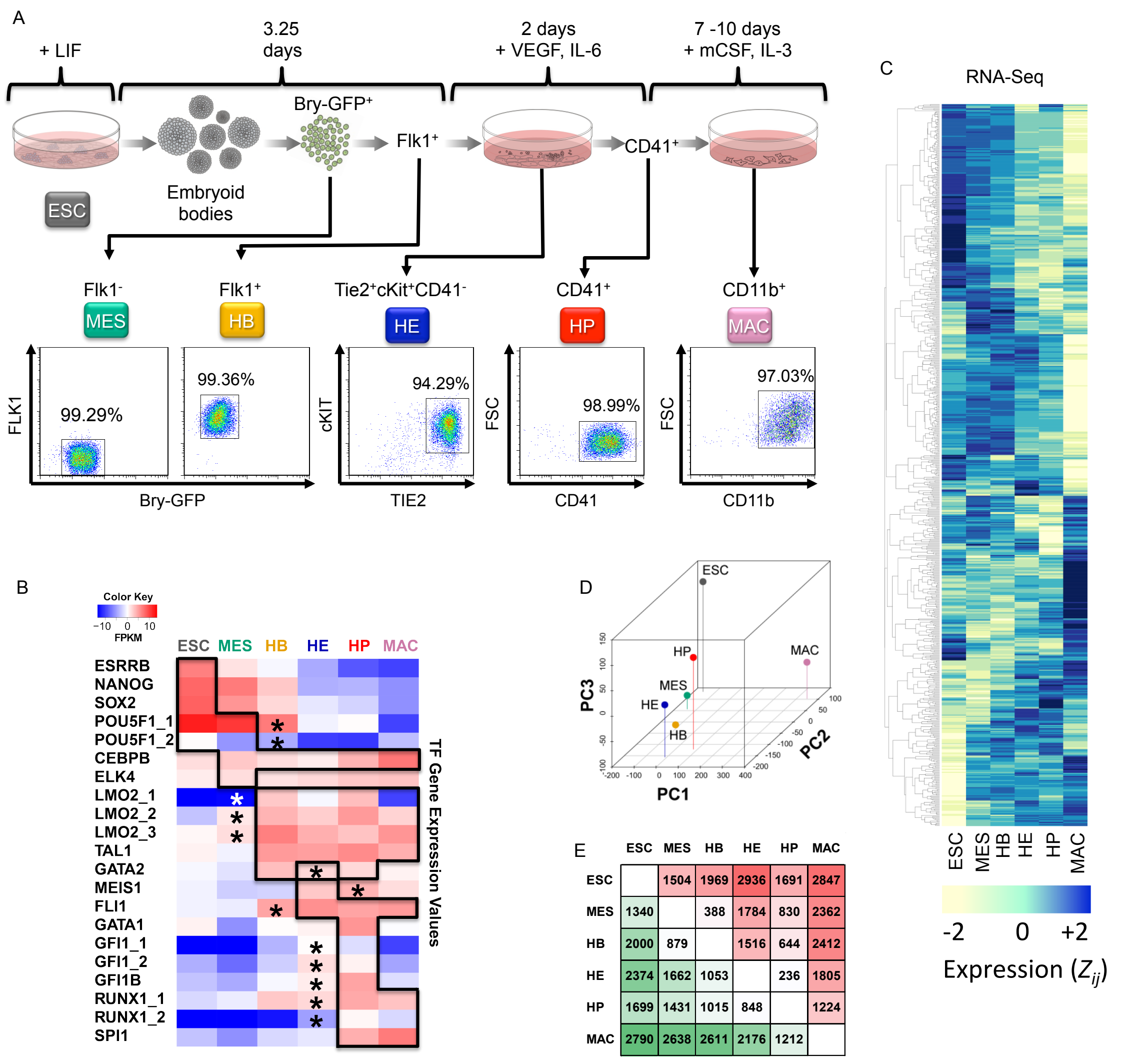


**Figure S1 (related to Figure 1):** (**A**) Schematic diagram of *in vitro* differentiation time course and isolation of cells at six different stages of development. ES cells are maintained in media supplemented with LIF, then allowed to differentiate into embryoid bodies. After just over three days, GFP positive cells (expressing Brachyury, Bry) are sorted into Flk1 negative (mesoderm, MES) and Flk1 positive (hemangioblast, HB) populations. Supplemented by cytokines (indicated), Flk1 positive cells are allowed to differentiate further towards hemogenic endothelium (HE), hematopoietic progenitors (HP) and finally macrophages (MAC). Each population is isolated using the sorting strategy indicated below. (**B**) Heatmap displaying of the expression of transcription factor genes

*($e_{ij}$*, see supplemental methods) that are two fold up- or down- regulated between any two pairs of cell lines across the developmental time course and computed as detailed in the methods section. Stages selected for TF ChIP experiments that passed our quality control are outlined by the black border and those that failed are asterisked. Note that some genes generate different isoforms and are listed multiple times. (**C)** Clustering analysis of the expression of genes ($Z_{ij}$, see supplementary methods) that are two fold up- (yellow) or down- (blue) regulated between any two pairs of cell lines computed as given in the methods section. (**D)** Principal Component Analyses of RNA-Seq data. The cell lines are projected onto the top three principal components that contribute to almost 90% of the variance in the gene expression. The contribution of a given principal component to the variation observed in the gene expression data are: PC1=56.5%; PC2=21.5%; PC3=10.8%. **(E)** Number of differentially expressed genes (Fold change ≥ 2 and FPKM ≥ 1) with down-regulated genes indicated in green, and up-regulated genes indicated in red.

A

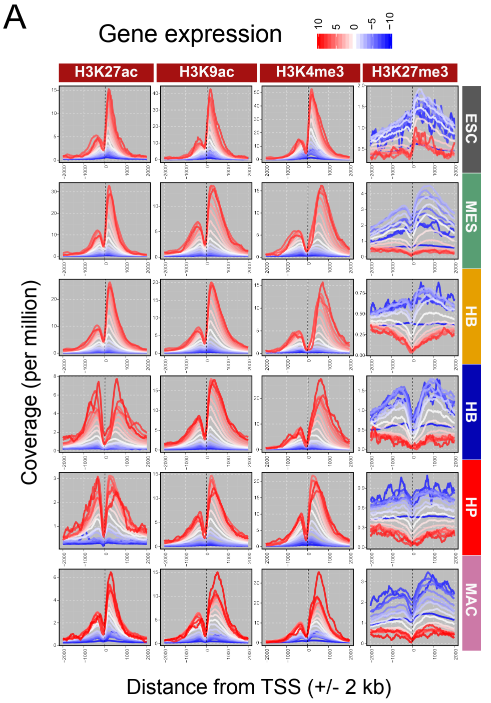


B

| Chromatin State ID | DHS | H3K27AC | H3K9AC | H3K4ME3 | H3K27ME3 | Coarse grained state |
|---|---|---|---|---|---|---|
| | Emission Probabilities | | | | | |
| 1 | 0.53 | 0.92 | 0.98 | 0.99 | 0.02 | Active |
| 2 | 0.31 | 0.10 | 0.71 | 0.81 | 0.02 | Active |
| 18 | 0.56 | 0.92 | 0.88 | 0.07 | 0.00 | Active |
| 19 | 0.33 | 0.47 | 0.13 | 0.04 | 0.01 | Active |
| 22 | 0.07 | 0.13 | 0.63 | 0.00 | 0.00 | Active |
| 3 | 0.10 | 0.02 | 0.50 | 0.71 | 0.37 | Poised |
| 6 | 0.38 | 0.31 | 0.42 | 0.67 | 0.83 | Poised |
| 11 | 0.04 | 0.04 | 0.03 | 0.01 | 0.78 | Repressed |
| 4 | 0.00 | 0.00 | 0.00 | 0.01 | 0.00 | None |
| 5 | 0.00 | 0.00 | 0.00 | 0.03 | 0.01 | None |
| 7 | 0.00 | 0.00 | 0.00 | 0.00 | 0.00 | None |
| 8 | 0.01 | 0.00 | 0.00 | 0.00 | 0.21 | None |
| 9 | 0.00 | 0.00 | 0.00 | 0.00 | 0.00 | None |
| 10 | 0.00 | 0.00 | 0.00 | 0.00 | 0.00 | None |
| 12 | 0.00 | 0.01 | 0.01 | 0.00 | 0.04 | None |
| 13 | 0.00 | 0.00 | 0.00 | 0.00 | 0.01 | None |
| 14 | 0.00 | 0.03 | 0.02 | 0.00 | 0.09 | None |
| 15 | 0.02 | 0.26 | 0.12 | 0.01 | 0.25 | None |
| 16 | 0.01 | 0.27 | 0.05 | 0.00 | 0.01 | None |
| 17 | 0.01 | 0.03 | 0.03 | 0.00 | 0.00 | None |
| 23 | 0.00 | 0.00 | 0.02 | 0.00 | 0.00 | None |
| 21 | 0.01 | 0.00 | 0.02 | 0.00 | 0.01 | None |
| 20 | 0.78 | 0.13 | 0.09 | 0.07 | 0.07 | DHSOnly |

C

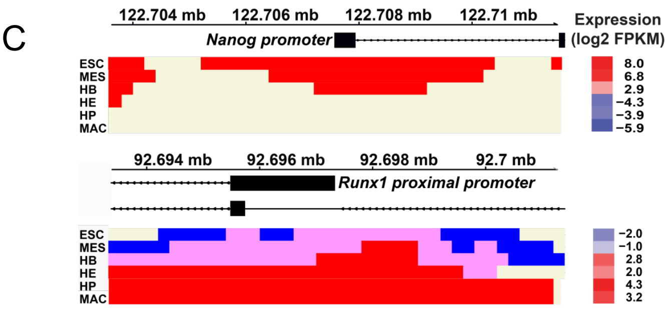


D

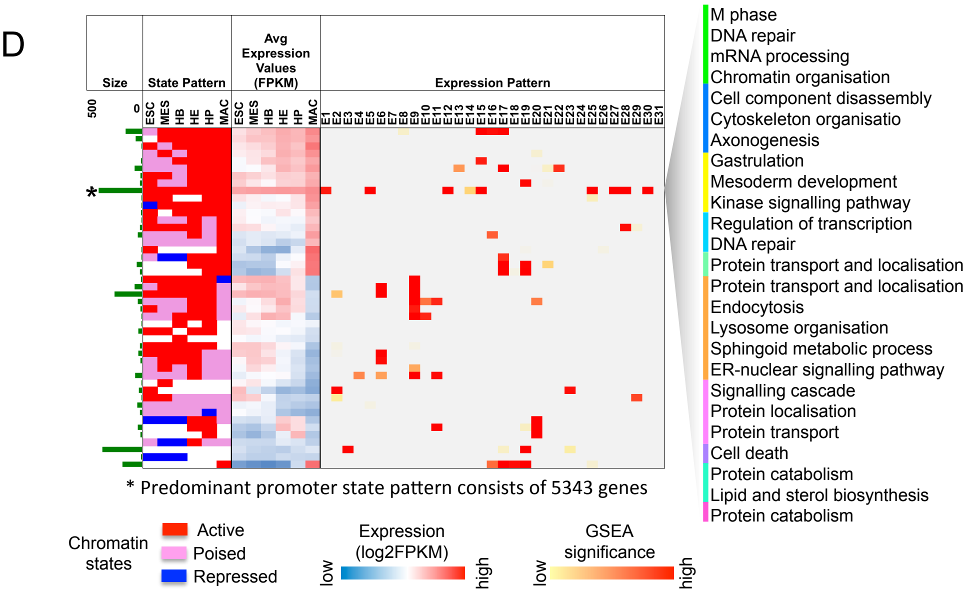


**Figure S2 (related to Figure 2)**: **(A)** The average tag density profiles, calculated as coverage per million (cpm), of histone marks around the TSSs clustered according to the expression values of their associated genes. For each chromatin mark (column) in a given cell line (row) the tag counts (bin size of 20bp) were computed within ± 2kb of the TSSs of all coding regions and normalised as per million aligned reads. The TSSs were grouped

according to their expression values ($\log_2(FPKM)$) and for each group the tag counts were averaged for each bin and plotted. Therefore the plot shows the average tag density profile of a given histone mark around the TSS clustered according to the gene expression values. **(B)** Emission probabilities of the 23 states identified from the chromHMM (see supplementary methods section) model obtained from all four histone marks ChIP-seq and the DNaseI-seq data. The coarse-grained states used further in the analyses are given in the last column. **(C)** Example of the chromatin states at the promoters (promoter states) of *Nanog* and *Runx1* with their expression values given at the right. **(D)** Correlation between the promoter state patterns and gene expression patterns. The predominant chromatin states at the promoters were identified as described in the supplementary data analysis section. Promoters with the same state pattern (second column) were clustered together and their population sizes are given as a bar chart in the first column. The average expression values for the genes belonging to the promoters are given in the third column. The significance of overlap between a gene set belonging to a state pattern and an expression pattern (E1 to E31) is denoted as yellow to red boxes scaled according to *p*-values obtained from hypergeometric tests (Gene Set Enrichment Analyses, GSEA, see scale bar). *p*-values greater than 0.05 are given in grey. The functional descriptions given on the right relate to GO term enrichment for the 10 clusters of genes (denoted by colour coded bars, see Table S3) identified in Figure 2B. These genes are associated with the predominant promoter pattern (always active) indicated by the asterisk in the left hand bar chart.

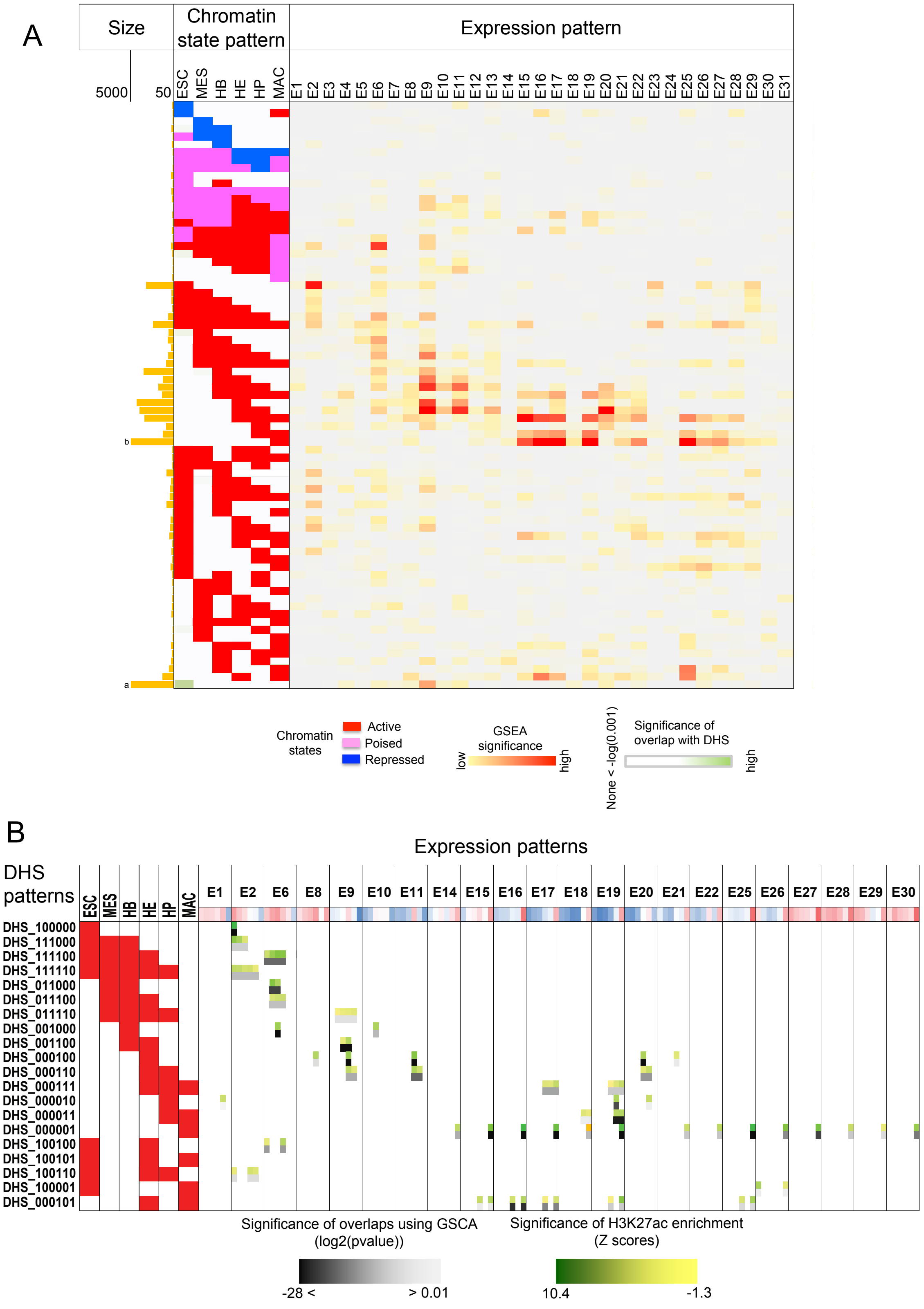


**Figure S3 (related to Figure 3): (A)** The predominant chromatin states for the distal DHSs were identified from the coarse-grained chromHMM model, similar to Figure S2D. The distal sites were clustered together based on matching chromatin state patterns (CSP) across all the six cell populations and only CSPs consisting of 50 or more elements were considered. In the heatmap (left panel) each row represents a CSP with the yellow bar chart to its left displaying the total number of regulatory sites (size) in each CSP. For a given set of genes associated with CSP (rows) and a specific expression pattern

(columns), the overlap significance is computed using hypergeometric testing and is adjusted for multiple testing using the Benjamini Hochberg method. The heatmap on the right panel shows the significance of overlaps (-log(p-value)) where the non-significant overlaps (p-value > 0.01) are shown in grey. a: total peaks 23114, b: total peaks 9837. Integrated into the CSP heatmap is a green heatmap showing the significance of overlap of each CSP with DHS in each cell population calculated from hypergeometric testing. A green colour observed in the CSP that is "None" indicates DHS without accompanying histone modifications. This is evident in the 23114 peaks in the last row ('a' in the left hand bar chart) which consists of DHSs without accompanying histone modifications. The next most prevalent CSP, b (total peaks 9837), consist of a stage specific pattern in macrophages. **(B)** Correlation of DHS-pattern frequencies (rows) with gene expression patterns (columns). The average log2(*FPKM*) for genes in a given expression pattern is plotted as the blue-red heatmap in the top row. DHS patterns are indicated at the left, using both a binary code (1=presence) and a grid depiction (red=presence) to denote presence at a specific developmental stage. GSCA was used to calculate the significance of overlaps between a given DHS pattern and an expression pattern and is plotted as a grey scale bar. Only those patterns that were significantly enriched are shown (p-value <0.01). For each DHS pattern, if the hypersensitive sites are open at a given cell stage, the significance of H3K27ac enrichment was calculated by bootstrapping and this is plotted as a yellow-green scale bar.

A

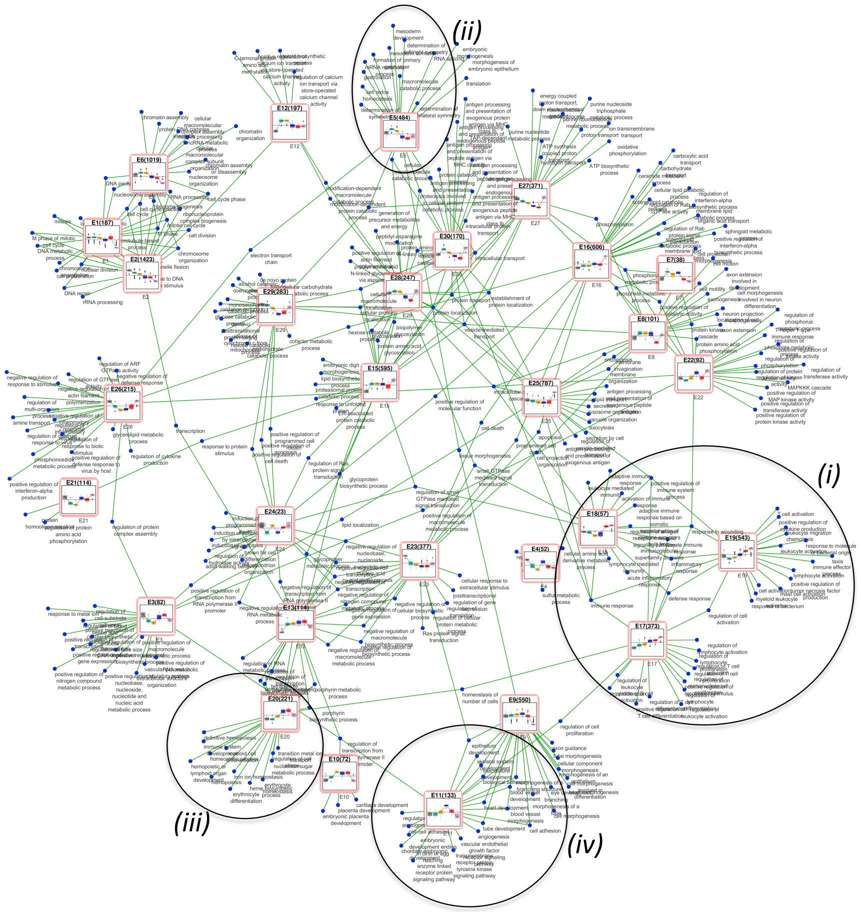


http://www.bioinformatics.leeds.ac.uk/labpages/hematopoiesis/wp-content/uploads/sites/3/2015/02/goNet_2.svg

B

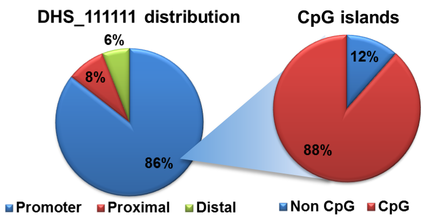


C

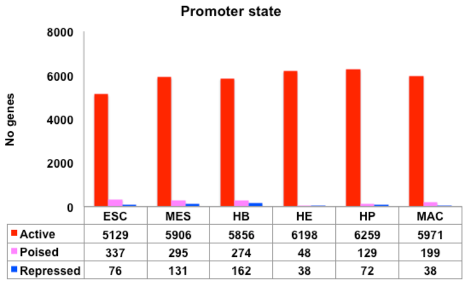


**Figure S4 (related to Figure 3 and 4): (A)** Network of the top 20 GO terms with the best *p*-values in each of the 31 expression patterns depicted in Figure 1F. The node set comprises patterns E1 to E31 and the enriched GO terms and edges are drawn from the GO terms to the pattern in which it is over-represented. Details can be seen by zooming in or by visiting http://www.bioinformatics.leeds.ac.uk/labpages/hematopoiesis/expression-pattern-gene-ontology-network/. The layout aims at grouping the expression patterns with shared functions in the centre, with the periphery populated by expression patterns with unique functional annotations. A few important biological processes and the associated expression patterns are highlighted in circles (i) Immune response (ii) Mesoderm development (iii) Hematopoiesis and (iv) Vascular and endothelial development. The expression patterns in the centre share a large number of enriched GO terms and interestingly form a backbone of essential cellular housekeeping processes. **(B)**

Distribution of DHS_111111 relative to TSS: Promoter (±2kb), proximal (±20kb) and distal (> 20kb). The majority occur at promoters which predominantly consist of CpG islands (right hand pie chart). (**C)** The state of the promoter (active/poised/repressed) at genes associated with DHS_111111 regions.

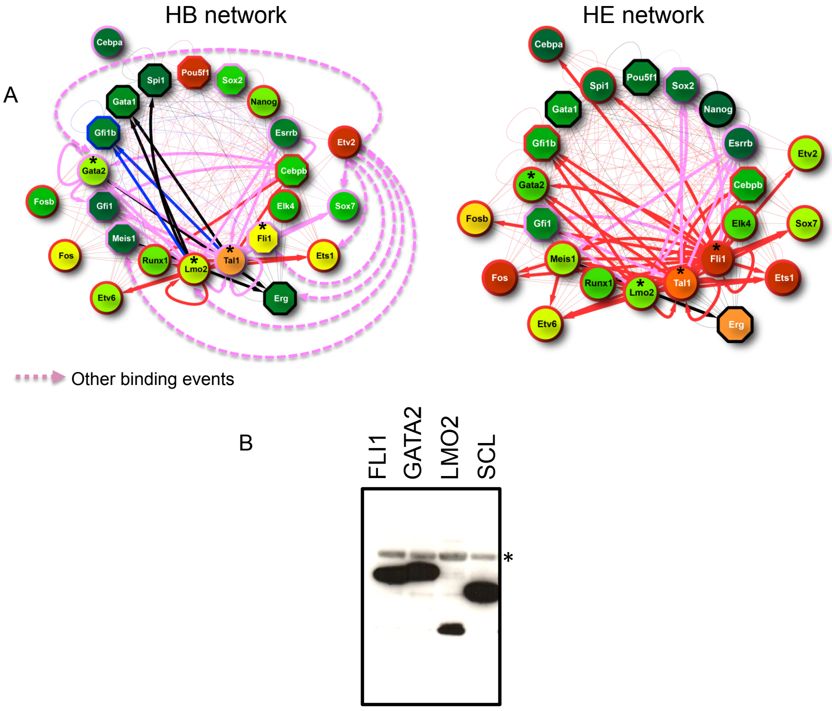


**Figure S5 (related to Figure 4 and 5): (A)** Expanded gene regulatory networks bordering the major hematopoietic transition T3. The 4 core reprogramming TFs identified in Figure 5 are indicated by asterisks. Additional TFs previously found to influence haematopoietic reprogramming are shown in peripheral nodes with binding events to these loci indicated by arrows. Dashed arrows indicate evidence of reciprocal binding events from independent ChIP-seq experiments at equivalent *in vitro* stages. Evidence from the ETV2-*Sox7* interaction comes from qPCR analyses of HA-tagged Etv2 ChIP experiments from day 3.5 EBs and pull-down assays (Behrens et al 2014). qPCR of *Etv2* null mice indicates that early *Fli1* expression (E8.0) is Etv2 dependent. This is corroborated by luciferase reporter gene assays and targeted mutagenesis of conserved binding sites in the Fli1 promoter and found to be cell-type specific (Abedin et al 2014). ChIP analyses of day 3-3.5 EBs identified an ETV2 binding event 1 kb upstream of the *Lmo2* TSS, corroborated by EMSA (Koyano-Nakagawa et al 2012). **(B)** Transduced cells express all four constructs. MEFs were transduced with indicated FLAG tagged transcription factor expressing viruses. Cells were harvested at day 4 and cell lysates were subjected to western blotting with anti-FLAG antibodies. * Nonspecific band indicating equal loading.

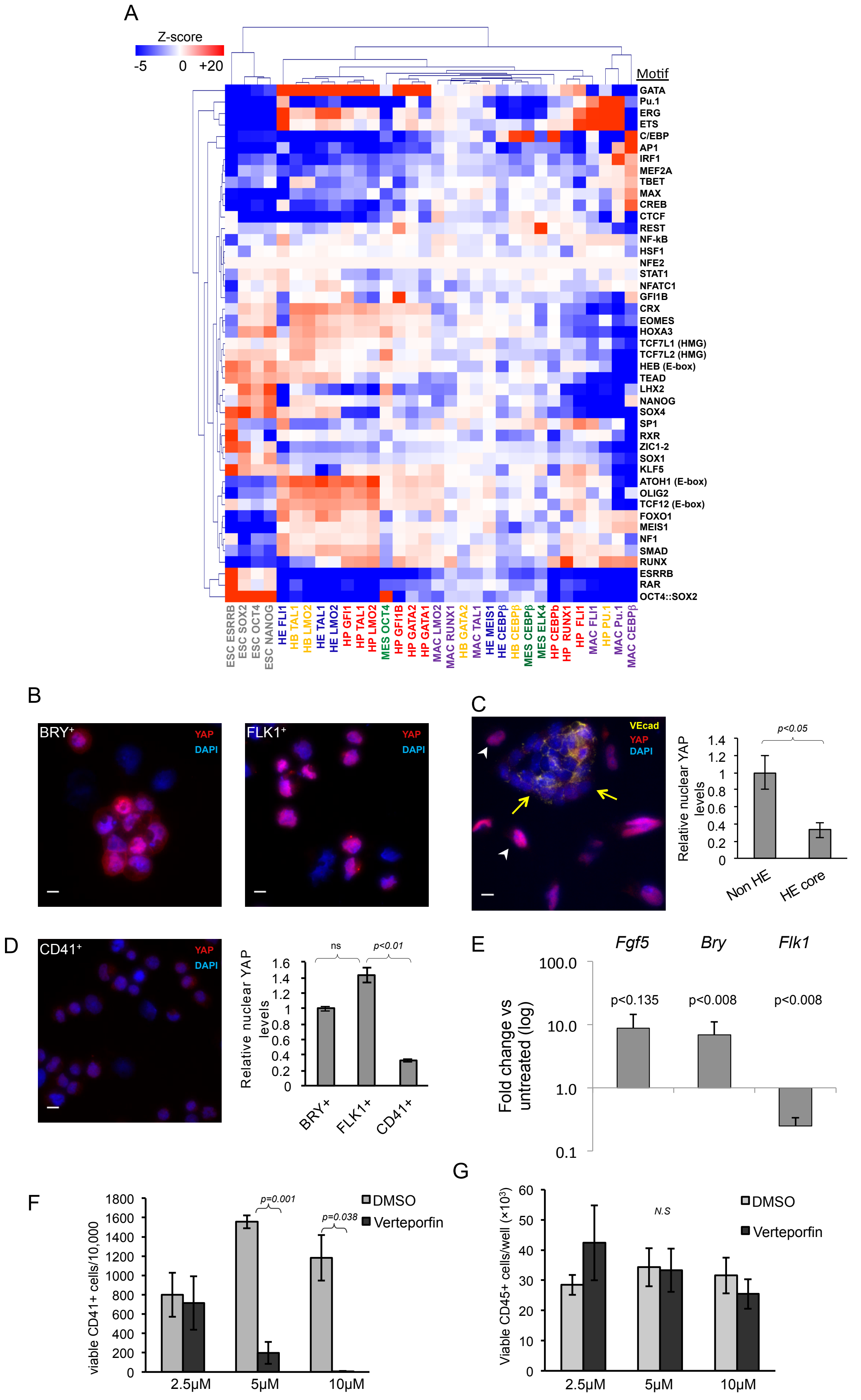


**Figure S6 (related to Figure 6 and 7): (A)** Relative co-localizing motif frequencies within ChIP peaks of the 32 transcription factors examined in this study (for used motifs see

Table S6). The motif Relative Enrichment (RE) scores within the ChIP peaks of 32 TFs were compared to RE scores of those motifs for a random set of peaks. Z scores were calculated from the mean and standard deviation of random RE scores using bootstrapping analysis. **(B, C & D)** TEAD activity peaks during the early phase of hematopoietic commitment. **(B,D)** Cytospins of EB derived $BRY^+/FLK1^-$ ME ($BRY^+$), $BRY^+/FLK1^+$ HB ($FLK1^+$) and $CD41^+$ HP populations. Cytospins were stained for TEAD co-activator YAP (red) and nuclei were visualised with DAPI (blue), scale bars represent 10μm. Graph shows the quantified relative nuclear YAP levels. Data are presented as mean intensity ± SEM, *n*=5, *t*-test. (**C)** HE culture stained for TEAD co-activator YAP (red). HE cores are identified as VE-cadherin positive (yellow) clustered cells. Relative nuclear YAP levels were calculated for the cells within HE cores (arrow) compared with non-HE surrounding cells (arrow head). Data are presented as mean intensity ± SEM, *n*=5 *t*-test. **(E)** $BRY^+/FLK1^-$ ME cells were cultured in the presence or absence of verteporfin (9.6 μM). After 48h RNA was isolated and the expression of *Fgf5*, *Bry* and *Flk1* was measured by qPCR. Gene expression in verteporfin treated cells was normalised to expression in DMSO control treated cells. Data for five biological replicates are presented as mean + SEM, Mann Whitney U. **(F)** Inhibition of TEAD – YAP interaction blocks hematopoietic differentiation and survival in explant cultures of cells from early but not late stages of hematopoietic specification. $FLK1^+/CD41^-$ HE enriched cells were sorted from E7.5 embryos by FACS and cultured for 4 days on irradiated OP9 treated with 2.5μM, 5μM and 10μM verteporfin or DMSO. Hematopoietic differentiation of HE cells was measured by staining with a CD41-PE antibody. **(G)** Verteporfin treatment of $CD45^+$ committed hematopoietic progenitor cells sorted from E10.5 embryos by FACS and cultured on irradiated OP9 in hematopoietic cell culture media (see Stroczynsca et al., 2009). Growth and survival of hematopoietic cells was assessed by staining with a CD45-FITC antibody.

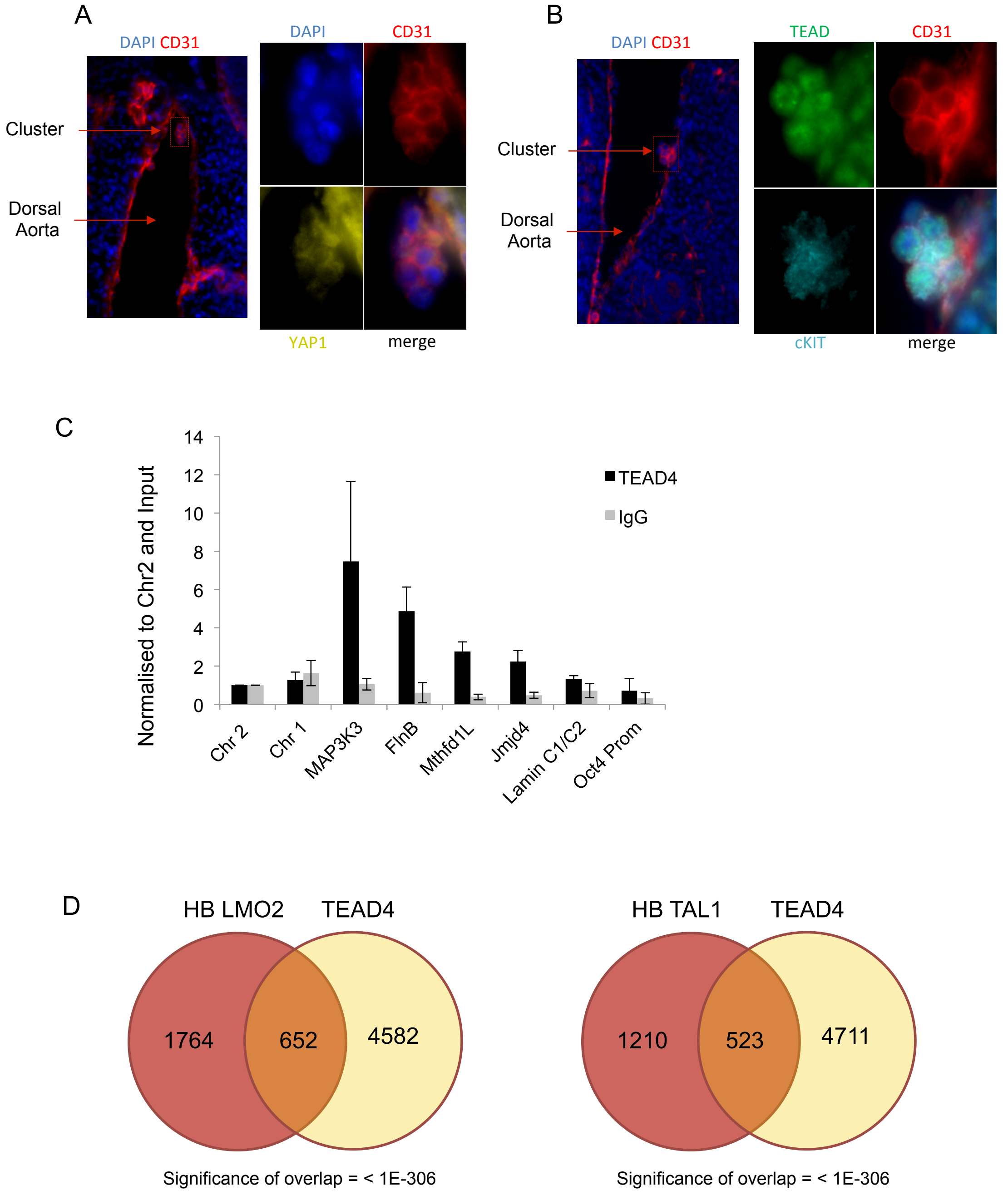


**Figure S7 (related to Figure 7)**: **(A)** TEAD and YAP do not localize to the nucleus of hematopoietic cells in E10.5 dorsal aorta clusters. E10.5 embryo sections were stained as indicated. (A and B) Left panels 40x magnification of DAPI and CD31; right panels 100x magnification of individual and merged stainings as indicated. **(B)** Manual validation of ChIP-Seq experiments. Binding of TEAD4 to TEAD-motif carrying elements (*Map3k3, FlnB, Mthfd1L, LaminC1/C2, JmjD4)* but not to control genes (all others). Data presented are mean ± SD (*n*=3). **(C)** Venn diagrams demonstrating that a significant number of TEAD4 peaks overlap with TAL1 and LMO2 peaks in hemangioblasts (HBs). The significances were calculated as p-values from hypergeometric tests.

## Supplementary Tables

**2. Table S1 (related to Figure 1, 2)**
(A) Antibodies used for ChIP-Experiments and details of sequencing runs, (B) Quality control of ChIP-Seq experiments

**3. Table S2 (related to Figures 1, 2, S1, S2):**

(A) Genes that are 2 fold differentially regulated during different transitions along the differentiation pathway, (B) Transcription factor genes that are 2-fold differentially regulated, (C) Over-represented GO terms in differentially regulated genes with FPKM >= 1 in at least one cell line

**4. Table S3 (related to Figures 1, 2, 3, S1, S2, S4):**
(A) Raw expression values of different expression clusters, (B) Enriched GO terms of expression clusters

**5. Table S4 (Related to Figures 2 and S2):** Enriched GO terms of genes with constitutively active promoters.

**6. Table S5 (related to Figure 5)**: Sequence coverage of ChIP and DNaseI experiments, RNA-Seq data obtained in reprogramming experiments

**7. Table S6 (related to Figures 6 and S6)**: Position weight matrices used for motif clustering

**8. Table S7 (related to Figures 6 and S6):** Z-scores determined for clustering of motifs enriched in pairwise comparisons of DHSs

**9. Table S8 (related to Figures 7 and S7)**: KEGG pathway analysis of genes associated with TEAD4 peaks

**Table S6:**

**Position Weight Matrices used for motif clustering analyses**

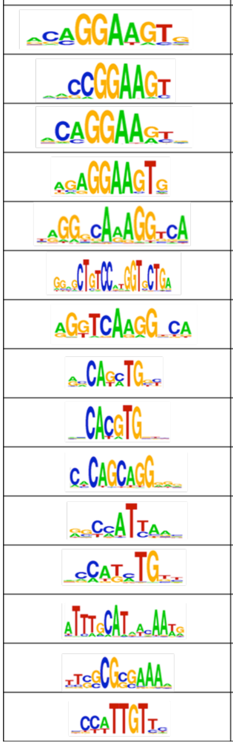

| LOGO | NAME |
|---|---|
| | ETS1 |
| | ETS |
| | ERG |
| | Pu.1 |
| | RXR |
| | REST |
| | RAR |
| | ATOH1 |
| | MAX |
| | HEB |
| | NANOG |
| | OLIG2 |
| | OCT4::SOX2 |
| | E2F |
| | SOX2 |

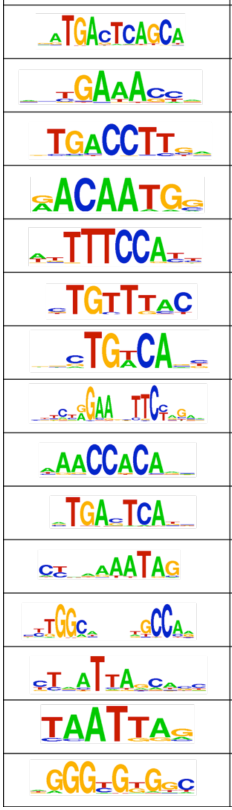

| LOGO | NAME |
|---|---|
| | NFE2 |
| | IRF4 |
| | ESSRB |
| | SOX9 |
| | NFATC1 |
| | FOXO1 |
| | MEIS1 |
| | HSF1 |
| | RUNX |
| | AP1 |
| | MEF2A |
| | NF1 |
| | HOXA3 |
| | LHX2 |
| | KLF5 |

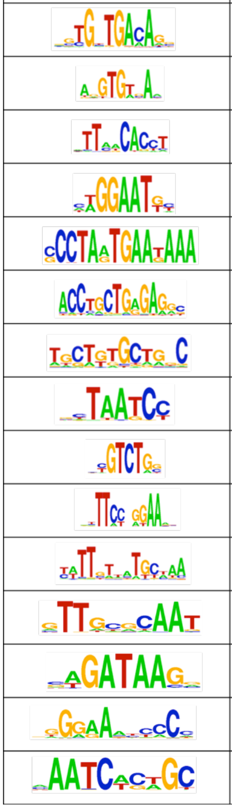

| LOGO | NAME |
|---|---|
| | TBX20 |
| | TBET |
| | EOMES |
| | TEAD |
| | SOX1 |
| | ZIC1-2 |
| | ZIC3-2 |
| | CRX |
| | SMAD4 |
| | STAT1 |
| | OCT4 |
| | C/EBP |
| | GATA |
| | NF-kB |
| | GFI1B |

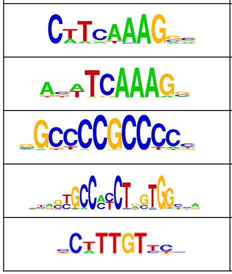

| LOGO | NAME |
|---|---|
| | TCF7L1 |
| | TCF7L2 |
| | SP1 |
| | CTCF |
| | SOX4 |

## 2. SUPPLEMENTAL EXPERIMENTAL PROCEDURES

### Purification of cell populations

A mouse ES cell line carrying a Brachyury-GFP reporter gene (Fehling et al., 2003) was cultured on MEFs then differentiated as described previously (Sroczynska et al 2009). Both GFP and cell surface markers were used to isolate each cell population, as outlined in Figure S1A. Briefly, after differentiation of ESC into embryoid bodies, mesodermal cells were isolated by FACs sorting GFP/Brachyury (Bry) positive cells that have yet to express the endothelial marker Flk1. A proportion of these cells were allowed to differentiate towards hemangioblasts (HB, $Bry^+/Flk1^+$), hemogenic endothelium (HE, $Tie2^+/cKit^+/CD41^-$) and hematopoietic progenitors (HP, $CD41^+$). Finally macrophages were isolated by terminal differentiation of $CD41^+$ cells to those expressing the macrophage marker CD11b.

**Isolation of cells for TEAD4 ChIP - Seq**: A single cell suspension of ES cells was transferred into IVD media on 15 cm low adherence bacteriological plates (Sterilin) at a concentration of 2.5 X 104/ml. IVD media - IMDM supplemented with 15 % FCS, 100 units/ml Penicillin and 100 µg/ml Streptomycin, 1 mM glutamine, 0.15 mM MTG, 0.18 mg/ml Human transferrin (Roche 652202) and 50 µg/ml ascorbic acid. After 3.25 days the embryoid bodies were collected, briefly digested in Tryple-Express protease mix (Life Technologies) and gently dissociated. A single cell suspension was obtained by passing the cells through a cell strainer and resuspending in IMDM + 20 % FCS. $Flk1^+$ (CD309) cells were isolated using a biotinylated Flk1 antibody (eBioscience 13-5821) at 5 µl per 107 cells for 15 minutes on ice, followed by 2 washes with MACS buffer (PBS + 5 % BSA and 0.5 mM EDTA). Cells bound by the antibody were then isolated using MACS anti-biotin beads and MACS LS columns (Miltenyi Biotec) according to the manufacturer's instructions. Isolated Flk1+ve cells were subsequently used for chromatin immunoprecipitation.

### Chromatin immunoprecipitation assays

**ChIP-Seq:** For each stage of development, cells were sorted, crosslinked and stored either as frozen cells (histone modification ChIP) or nuclei (TF ChIP) for subsequent ChIP assays, performed as described in detail previously (respectively Lichtinger et al 2012 and Wilson et al 2009; Forsberg et al 2000). The antibodies used in remaining ChIP assays are given in Table S1. For histone modifications around 2 million cells were used per experiment whereas 6-12.5 million cells were used for each TF ChIP assay, with the

exception of HEs, which constitute a rare transient cell population. In these cases an adapted carrier ChIP protocol (Zwart et al 2013) was used on around 1-2 million cells. Specifically 20 µg/ml recombinant histone 2B (M2505S; New England Biolabs) and 1 µg/ml mouse mRNA (cat# 338114, Qiagen) were added before the antibody at the immunoprecipitation step.

**Manual ChIP from Flk1$^+$ hemangioblast cells:** Chromatin immunoprecipitation was performed essentially as described in Lichtinger et al 2012 with the exception that the material was crosslinked with Di(N-succinimidyl)-glutarate for 45mins (DSG; Sigma) and 1% Formaldehyde for 10mins (Thermo Fisher) before quenching with 1/10th volume 2 M glycine. Primers used for ChIP analysis are shown in the table below.

| Gene | Forward Primer | Reverse Primer |
|---|---|---|
| Oct4 promoter | TGGGCTGAAATACTGGGTTC | TTGAATGTTCGTGTGCCAAT |
| MAP3K3 | TCACTGGTCTGGGGAGACTT | CCTTGCTATCTGATGGGCGA |
| FlnB | TGGGGTAGGGGCCAGATTAG | GACAATGGCTCCCTAGAGCTT |
| Jmjd4 | TGCAAGCTTCGACAATGACC | TGCAGCTACGTACTCTTCCC |
| Lamin C1/C2 | TCTCAGGAAACCGACCCATTC | CCCTCCGGTGAGTGTGAAAG |
| Mthfd1L | ATGCAAGCTGGGCAAATTCC | CCCAGAGTGCGGACAAAGAA |

**Reprogramming experiments:** Embryonic day 14.5 murine embryonic fibroblasts (MEFs) were prepared as described previously (Sroczynska et al., 2009). Cells were isolated from iScl-2A-GFP transgenic mouse carrying rtTA and TRE-SCL cassettes allowing inducible expression of SCL upon addition of doxycycline. Cells negative for CD45/CD41/CD31/CD117 were sorted from low passage MEFs and seeded on gelatine coated 12 well plates (1.5x10$^4$ cells/well). The next day the cells were transduced with different combinations of lentiviral vectors expressing FLI1, GATA2 or LMO2 in the presence of 10µg/ml DEAE-dextran. After four hours, cells were placed in haematopoietic medium (1X IMDM supplemented with plasma-derived serum (PDS; Antech), 10% protein-free hybridoma medium (PFM; Gibco), 0.5 mM Ascorbic Acid, 4.5x10$^{-4}$ M MTG, 2 mM L-glutamine, 80 mg/ml transferrin, 1% c-KIT ligand, 1% IL3, 1% of GM-CSF and 1% Thrombopoietin conditioned media, 4 U/ml Erythropoietin (Ortho-Biotech), 10 ng/ml M-CSF, 10 ng/ml IL6, 5 ng/ml IL11 (all from R&D Systems) and 50 µg/ml penicillin-streptomycin). Doxycycline (1µg/ml) was also added in some cases to induce SCL

overexpression and was replenished on days 5 and 9 to achieve continuous expression of SCL. Cultures were incubated at 37$^{o}$C in 5%$C0_2$ for 19 days at which point the number of reprogrammed blood cell colonies was scored.

**Immunostaining of in vitro differentiating cells:** BRY$^+$/FLK1$^-$ ME, BRY$^+$/FLK1$^+$ HB and CD41$^+$ HB populations were sorted from day 3, 4 and 7 EB cultures respectively, and adhered to slides by cytospin (5 minutes 400 rpm). In order to generate HE, day 3 EB-derived FLK1$^+$ cells were cultured for 48 hours on gelatinised µ-Slides (Ibidi). Cells were fixed with 2% paraformaldehyde (Sigma), permeabilised in PBS 0.2% Triton X-100 (Sigma), washed and stained with PBS, 10% FCS, 0.2% Tween 20 (Sigma). Cells were pre-blocked for 15 minutes with PBS, 10% horse serum and incubated with 1:100 anti-YAP (YAP_D8H1X XP® #14074 NEB) overnight at 4°C, followed by anti-rabbit AF647 or AF488 secondary antibodies (both Invitrogen; 1:1000 dilution) for 1 hour. HE cores in blast culture were identified by staining with anti-VEcadherin AF647 conjugated antibody (Alexa Fluor® 647 anti-mouse CD144 # 138005, Biolegend) overnight at 4°C. Slides were mounted with ProLong Gold antifade reagent with DAPI (Invitrogen). Images were captured using an Axiovert 200M (Zeiss) at 40x magnification, and nuclear YAP levels were calculated using Image J software as previously described (Arques et al., 2012).

**Immunostaining of embryos**

E7.5 in decidua and E10.5 embryos were fixed in 4% Paraformaldehyde (PFA) for two hours, soaked in 30% sucrose and mounted in OCT compound. 7µm sections were blocked in PBS with 10% FCS, 0.05% Tween20 and 10% goat serum (DAKO) for 1 hour before the sections were incubated with primary antibodies at 4°C overnight in blocking buffer. Primary antibodies used in this study were rabbit Anti Pan-TEAD (D3F7L) (1/100); rabbit Anti YAP (D8HIX XP) (1/100); Purified Anti-Mouse Tie2 (Tek-CD202B) (14-5987-85) (1/100). Sections were washed 3x in PBST for 15 minutes each and then incubated with fluorochrome-conjugated secondary antibody at room temperature for 1 hour. Secondary antibodies used in this study include Alexa Fluor® 488 Goat Anti-Rat IgG (A11006, Life Technologies); Alexa Fluor® 647 $F(ab')_2$ Fragment of Goat Anti-Rabbit IgG (H+L) (A21246, Life Technologies). All secondary antibodies were used at 1/400 dilution. Sections were further washed 3x in PBS and mounted using Prolong Gold anti-fade medium with DAPI (Life Technologies). Images were taken using a low-light time lapse microscope (Leica) using the Metamorph imaging software and processed using ImageJ.

**Inhibition of TEAD – YAP interaction with Verteporfin during ES cell differentiation.** The effect of TEAD inhibition during hematopoietic development was analysed in EB cultures in the absence or presence of the TEAD-YAP inhibitor Verteporfin (Liu-Chittenden et al., 2012, Brodowska et al., 2014) (Sigma). Verteporfin (stock solution 2.7 mM in DMSO) was added to day 1, 2, 3 or 4 EB cultures at a final concentration of 9.6µM. On day 7, EBs were disrupted with trypsin, stained with CD41-PE and CD45-brilliant violet 421(BD) and analysed by flow cytometry (LSRII BD). Cells were protected from light at all times. The $BRY^{+}/FLK1^{-}$ mesoderm enriched cell population was obtained from day 3 EB cultures using FACS and was re-plated in EB media in Ultra-Low Attachment plates (Corning/CoStar) at a concentration of 2E+5 cells / ml. The cells were cultured for 48 hours in the presence of 9.6 µM Verteporfin (Sigma) or vehicle (DMSO). Upon Verteporfin treatment the cells were protected from light at all times. RNA was extracted using the Spin Column RNA miniprep kit (NBS Biologicals). Complementary DNA was prepared using SuperScript III First-Strand Synthesis (Invitrogen). Quantitative PCR was performed using the Universal Probe Library System (Roche) using the following primers and probes: Fgf5 forward 5'-GTTTCCAGTGGAGCCCTTC-3', Fgf5 reverse 5'-GAGACACAGCAAATATTTCCAAAA-3' with probe 105; Bry forward 5'-CAGCCCACCTACTGGCTCTA-3', Bry reverse 5'-GAGCCTGGGGTGATGGTA-3' with probe 100; Flk1 forward 5'-AAAGCGGGACGAGGAGAG-3', Flk1 reverse 5'-CAGGTTGCACAGTAATTTCAGG-3' with probe 12.

**Inhibition of TEAD – YAP interaction *ex vivo***

$FLK1^{+}/CD41^{-}$ HE enriched cells were sorted from E7.5 embryos by FACs and cultured for 4 days on irradiated OP9 in HE media (IMDM containing 10%FBS, 4mM L-Glutamine, 50Units/ml Pen/Strep, 0.6% transferrin, 2% LIF, 48.75µg/ml monothiolglycerol, 25 µg/ml ascorbic acid, 2%LIF, 1%KL supernatant, 10ng/ml oncostatin M, 1ng/ml bFGF and 1µg/ml osteopontin) with 2.5µM, 5µM and 10µM verteporfin or DMSO. Hematopoietic differentiation of HE cells was measured by staining with a CD41-PE antibody and flow cytometry (LSRII BD). $CD45^{+}$ committed hematopoietic cells were sorted from E10.5 embryos by FACs and cultured on irradiated OP9 in hematopoietic cell culture media (see Stroczynsca et al., 2009) with increasing concentrations of verteporfin or DMSO. Growth and survival of hematopoietic cells was assessed by staining with a CD45-FITC antibody before the addition of counting beads (Thermo Fisher) and subsequent flow cytometry analysis. The number of viable $CD45^{+}$ cells/well was calculated by the ratio of viable

CD45$^{+}$ cells to counting beads according to the manufacturer's instructions (Thermo Fisher).

**RNA-Seq**

RNA preparation and sequencing were performed as described previously (Lie-A-Ling et al 2014), generating 50bp single-end reads from a SOLiD 5500xl sequencer (#4456991, Life Technologies).

**DNaseI-seq**

1-3 million freshly sorted cells were digested with DNase I enzyme as described previously, and size selected for 50 - 300 bp fragments as described in detail in (Ptasinska et al., 2012).

**Library generation and Sequencing**

DNaseI and ChIP samples were amplified and sequenced according to the manufacturer's instructions. Initial samples (MES, HB and HP H3K4me3, HP H3K27me3 and HP H3K27ac) were processed using an ABI SOLiD 4 sequencer, for all other libraries sequencing was conducted on either the Illumina 2G Genome Analyzer,the Hi-Seq 2000 or the Hi-Seq 2500.

**DATA ANALYSIS**

**Alignment**

Sequences from all experiments were mapped onto the reference mouse genome version mm10, Genome Reference Consortium GRCm38. The adapters were removed from reads from the Illumina sequencer using CutAdapt (http://code.google.com/p/cutadapt/) and trimmed for low quality reads (less than 20). The quality control statistics for the reads were obtained using FastQC (http://www.bioinformatics.babraham.ac.uk/projects/fastqc/). The trimmed Illumina reads were aligned to the mouse genome using Bowtie (Langmead et al., 2009). The colour-space reads from the SOLiD sequencer (see Table S1) were aligned using SHRiMP (Rumble et al., 2009). The total number of aligned reads for all the experiments in this study is given in Table S5. Reads that were aligned to unique chromosomal positions were retained and duplicate reads were removed from the aligned data using Picard (http://broadinstitute.github.io/picard/). All further calculations were

performed on the filtered alignment data. The aligned RNA-seq and ChIP-seq reads were extended by 200bp from their 3' ends to generate density profiles in BigWig format. In the case of DNaseI-seq data, only the 5' ends of aligned reads were used for generating the density profiles. Separate density profiles for the positive and negative strand were generated for RNA-seq data. These tag densities can be viewed from our local instance of the UCSC genome browser (www.haemopoiesis.leeds.ac.uk).

**Peak Calling**

TF peaks were called using MACS (Feng et al 2011; Zhang et al 2008), whereby the p-value cutoffs were manually assessed after visually inspecting known reference genes (see Table S1). The quality of TF ChIP-seq data was further refined using the polyaPeak R-package (Wu and Ji, 2014). Enriched regions of histone acetylation (H3K27ac, and H3K9ac) were obtained using DFilter (default parameters) and MACS and the coordinates from DFilter (Kumar et al., 2013) that overlap with MACS peaks were considered as acetylation peaks for subsequent analyses. For each cell population, the respective ChIP inputs were used as control data during peak calling. DNaseI Hypersensitive sites (DHSs) are those that are called as peaks by DFilter (default parameters) in the DNaseI-seq data. The total number of TF and histone modification peaks and DHSs in the dataset is provided in Table S1.

**RNA-seq data analysis**

***Identification of differentially expressed genes***

The total number of tags that overlap with the genes (exonic region only) were obtained using HTSeq (Anders et al., 2015) and differential expression analysis for the coding genes were performed using DESeq (Anders and Huber, 2010; Anders et al., 2013) using the count data. Genes were said to be differentially expressed (DE) if there was a twofold change in expression between any two cell lines with a *p*-value less than or equal to 0.01. Over-represented Gene Ontology (GO) terms (Ashburner et al., 2000) identified using DAVID (Huang da et al., 2009a, b) as well as gene family classification (MSigDB (Liberzon et al., 2011)) can be seen in Table S2. We identified 8986 genes to be differentially expressed between any pair of cell types from our DESeq analysis (Figure S1E) that also have an expression value of at least 1 FPKM (from cufflinks) in any of our cell types.

***Gene expression quantification***

Cufflinks (Trapnell et al., 2012) was used to calculate the expression values as Fragments Per Kilobase per Million aligned reads (FPKM) from the aligned RNA-seq data. Both gene-

wise expression values and TSS based expression values were obtained using the *cuffdiff* function of cufflinks. The correlation between any two cell lines was obtained as the Pearson correlation coefficient of expression values over all genes. A correlation matrix was thus generated for all the cell lines and hierarchically clustered to study the relationship among cell lines as given in Figure 1B. The gene-wise expression values for each developmental stage were used to calculate the principal components using *princomp* package from R. The top three principal components were found to contribute to almost 90% (Figure S1D) of the variance observed in the expression data. Figure S1D shows the cell stages projected onto these three principal components.

***Clustering of differentially expressed genes***

The 9627 unique TSSs belonging to the 8986 DE genes with FPKM greater than 1 in at least one cell population (Table S2) were then clustered into 31 expression patterns (Figure 1F) using *k*-means and hierarchical clustering as follows. For each TSS (*i*) of a DE gene a pseudo-count $\gamma = 0.001$ was added to the TSS-based FPKM values and the binary logarithm of this value was considered as the expression value of the TSS in each cell type (*j*), $e_{ij} = log_2(FPKM_{ij} + \gamma)$. The expression data was further quartile normalised using the *LPE* package in R to give standardised expression values, and then converted to Z-scores ($z_{ij} = \frac{e_{ij} - \mu_i}{\sigma_i}$) where $\mu_i$, is the mean expression and $\sigma_i$ is standard deviation of the gene "*i*" across all the cell lines for clustering purposes. The Z-scores of the genes (TSSs) were initially clustered using *k*-means clustering with Euclidean distances (*stats* package in R) and the optimal number of clusters was determined to be 650 based on the lowest Bayesian Information Criterion (BIC) scores (Schwarz, 1978). The centroids of the *k*=650 clusters were calculated as the mean of Z scores of the members of the clusters. These centroids were then hierarchically clustered using the "complete linkage" agglomeration method (Lance, 1967) and the tree was cut into 31 patterns.

The GO terms that are over-represented (*p*-values less than 0.05) in genes that are up and down-regulated at different transition points (T1 to T5), and within different expression patterns, were obtained using DAVID and are summarised in Figure 1E, and also in Table S2 and Table S5, respectively. RNA-seq data for each cell-type were performed as replicates and both DESeq and Cufflinks, used in our analysis, are designed to account for the significance of reproducibility among the replicates

**Functional network of expression patterns**

The top 20 over-represented GO terms with the best *p*-values were identified for each of the 31 expression patterns. Undirected edges were drawn between the pattern and the shortlisted GO terms, and therefore GO terms that are shared among expression patterns will have a vertex degree of greater than one. Cytoscape (Cline et al., 2007) was used to generate a visual representation of the network using an edge-weighted spring embedded layout consisting of expression patterns and the associated GO terms (Figure S4A; http://www.haemopoiesis.leeds.ac.uk/data/networks/goNet.svg).

**DNaseI-seq data analysis**

The 50bp reads were aligned to mouse genome and the bigWig files were generated for visualisation as mentioned earlier. DNaseI-seq peaks or DNaseI Hypersensitive Sites (DHSs) were obtained using DFilter with recommended parameters (-bs=100 -ks=50 –refine). Pair-wise peak overlaps between DHSs of any two cell lines *a* and *b* were performed using BedTools (Quinlan and Hall, 2010) in order to calculate the fraction ($M_{ab}$)

$$M_{ab} = \frac{N_{ab}}{N_a}$$

where $N_{ab}$ is the total peaks that overlap (minimal overlap of 1bp), $N_a$ is the total number peaks in set *a* and $N_b$ is the total peaks in *b*. Given $N_a$, $N_b$, $N_{ab}$ and the total DHSs in all cell lines, a hypergeometric test was used to compute the statistical significance of the overlap. It should be noted that M is an asymmetric matrix as $M_{ab} \neq M_{ba}$. $M_{ab}$ is not considered, i.e. $M_{ab}$=0, if the p-value for the overlap is greater than 0.05, thereby generating a corrected overlap matrix *M'*. Pearson correlation coefficients were calculated between any two rows of the matrix *M'* and this resultant correlation matrix was hierarchically clustered. Finally, the R package cba was used for optimal ordering of nodes in the dendrogram and a heatmap was generated using the gplots R package and presented in Figure 1D.

**Clustering of DHSs based on their binary patterns**

The DHSs from all cell lines were standardised to 500 bp around the summit and merged using BedTools (Quinlan and Hall, 2010) if DHS sites overlap by at least 125bp, to form a master set of 96195 regions. The dynamics of chromatin accessibility is captured as binary (open=1; closed=0) clusters of DHSs. Each of the merged regions was given in a binary code of 6 digits based on the presence (*1*) or absence (*0*) of DHS in all the six cell types. For example, DHS_100000 is a hypersensitive site that is open only in ESC while DHS_000011 denotes a site that is open both in HP and MAC. All 96195 merged regions were then clustered based on their binary patterns and Figure 2D shows the most

predominant patterns (population size of the pattern greater than 100). A hypersensitive site is deemed as "distal" if it is greater than 2kb from the TSS of the nearest coding gene. *De novo* motifs were identified for the distal DHS sites and matched to known motifs using HOMER (Heinz et al., 2010). A motif was termed as over-represented if its *p*-value was less than 0.01 and its frequency of occurrence in the peaks (HOMER default cutoff) was at least 10%. Over-represented motifs obtained from stage specific distal DHSs (DHS_100000, DHS_010000, DHS_001000, DHS_000100, DHS_000010 and DHS_000001) were used to assemble a master set of PWMs for interrogating all distal DHSs (Table S6). Figure 6B shows the hierarchically clustered relative enrichment (RE) scores for each of these motifs across various DHS sets. RE scores were calculated as given below.

**Motif identification and clustering**

To identify motifs (identified by HOMER as above) that are relatively enriched in the distal DHS sites of one cell type compared to another we considered all possible cell-type to cell-type comparisons (Figure 6A). For each cell type (A) we defined 5 sets of DHS sites, these being DHS sites in (A) which are not shared with each of the 5 other cell types (B). For 6 cell types (A) this yields a total of 6x5=30 sets of DHS sites. For a given set *j* of DHSs (in Figure 4A), we defined a Relative Enrichment (RE) score for motif *i* in peak set *j* as

$$RE_{ij} = \frac{n_{ij}/M_j}{\sum_j n_{ij}/\sum_j M_j}$$

where $n_{ij}$ is the number of peaks in each subset *j* *(j=1,2,…,30)* containing motif *i* *(i=1, 2,….,I), I* is the total number of motifs used in the test, and $M_j$ the total number of peaks in each subset *j* *(j=1,2,…,30).* A matrix was generated and the relative motif scores were displayed as a heatmap after hierarchical clustering with Euclidean distance and complete linkage. The heatmap was generated using Mev from TM4 microarray software suite (Saeed et al., 2006). The relative motif enrichments were also calculated in similar way within the TF ChIP-Seq peak sets (Figure S6A). A matrix was generated and the motif relative enrichments were displayed as a heatmap after hierarchical clustering with Euclidean distance and complete linkage. The TEAD motif co-localization within the ChIP-Seq peaks (Figure S6C) shows the log2 enrichment score for visualization. The statistical significance for a $RE_{ij}$ score of a given motif *i* in peak set *j* is computed as Z-scores using bootstrapping (N=1000), where a random set of peaks is extracted from a global set of regions and *RE* is calculated. After N iterations the mean ($\mu_{ij}$) and the standard deviation ($\sigma_{ij}$) are computed and the z-scores are computed as $Z_{ij} = \frac{RE_{ij} - \mu_{ij}}{\sigma_{ij}}$. The global set of

regions is a merged set of all the DHS for Figure 6B and a merged set of all TF peaks in Figure S6A. These Z-scores are provided in Table S7.

**ChIP-seq data analysis**

The single-end 50 bp reads were aligned to the mouse genome as mentioned earlier. The TF peaks were called using MACS (Feng et al 2011; Zhang et al 2008) and were standardised to 400bp around the peak summits. Fractional overlap matrix (*M'*) was calculated for the TF ChIP-seq peak sets similar to the methodology used for computing correlation among DHSs, as mentioned in DNaseI-seq analysis section. For robust analyses, correlation values among the TF ChIP-seq peaks were calculated from binding events that occurred in at least two different experiments. The hierarchically clustered correlations among the different TF binding sites (TFBS) are shown in Figure 1D.

The histone mark peaks were identified using DFilter with the following recommended parameters. For H3K27ac and H3K9ac the peaks were called using -bs=100 -ks=60 -std=2, while for H3K27me3 peaks were called using -bs=100 -ks=30 -nonzero -std=2, and H3K4me3 peaks were called using -bs=100 -ks=100 -std=2 options. The total peaks in each experiment are given in Table S1. The H3K27ac peaks were further refined as the consensus peak regions of both DFilter and MACS (default parameters) and this peak set is used as in our integrated data viewer.

http://www.bioinformatics.leeds.ac.uk/labpages/hematopoiesis/)

The average tag density profiles of the histone marks around the TSSs of coding genes, given in Figure S2A, were calculated as a function of the expression values of the TSS as given below.

**Average tag density profile of histone marks**

The average tag density profiles for each histone mark in a given cell line, around the TSS were generated by calculating the mean tag density normalised as coverage per million (CPM), within 2kb of the TSSs of all genes. The TSSs were grouped into different classes based on their expression values at each developmental stage. For example, a TSS is classified as 1 if its expression value $e_{ij}$ defined above on log scale lies between 1 and 2, 2 if its expression value lies between 2 and 3 and so on until 10 which are given to any expression value greater than 10. The same classification applies to the negative scale of the expression values. The tag density profiles were computed individually for each expression class and rolling averages over 50bp windows were used to generate the

density profiles. The rolling averages were computed using *zoo* and the profiles were plotted using *ggplot2* in R.

### Chromatin state identification

The chromatin states were learned using the DNaseI-seq and the histone marks ChIP-seq alignment data using chromHMM (Ernst and Kellis, 2012). The optimal number of states were derived from BIC scores (for three random initialisations, and 5000 iterations with a convergence value of less than 0.001) calculated as $-\ln L + k\ln(n)$ where $k=s(s+5)$, $s$ is the number of states = $\{1,2,..,31\}$, $n$ is the total number of bins the genome was divided into and $-\ln L$ is the log likelihood estimation obtained from chromHMM. The lowest BIC score was obtained for 23 states in all the three random initialisations. Based on the emission probabilities of the 23 optimal states, the states were further manually coarse-grained into four, namely the Active, Poised, Repressed or None (Figure S2B).

### Promoter state clustering

The fractional contribution profile (FCP) of each of the four states to a promoter in a given cell line is obtained as given below (Figure 2A and Figure 2B). A promoter is defined as ±1kb from the TSS and the fractional occurrence of each of the four states is computed as the fraction of the total number of bases designated as Active, Poised, Repressed or None and is termed as Fractional Contribution Profile (FCP). FCPs were calculated for all the promoters at each stage of development and were used to hierarchical cluster the TSSs with the "complete linkage" method, based on euclidean distances. A single predominant state (with at least 30% occupancy) was assigned to each promoter and colour coded in the heatmap (Figure 2B left panel, Figure S2D). The standardised expression values (z) of the genes belonging to promoters that are always active (Figure 2B; right panel) were hierarchically clustered and the tree was cut into 10 different sub-clusters. The functional annotations of these clusters are given in Figure S2D (and in Table S3).

## Integration of 4-dimensional data

### Comparison of TF binding events and chromatin accessibility

The binary DHS patterns were obtained as given earlier and plotted with heatmap.2 (gplots package in R http://www.r-project.org/). Genomic coordinates of these patterns were intersected with the TF binding events that occurred in at least two different experiments using BedTools. The statistical significances of such overlaps were calculated as *p*-values using hypergeometric testing. Significant overlaps ($p$-value < $10^{-4}$) were

overlaid onto the DHS heatmap as a dot plot (geom_point function from the ggplot2 package in R) and given in Figure 2D.

**Integration of chromatin states at the promoter and expression pattern**

Figure S2D describes the correlation between promoter states and expression patterns. The promoters were clustered if they have same Active/Repressed/Poised/None state in the six cell lines. In a similar process to Gene Set Enrichment Analysis (GSEA), the statistical significance of overlap between a given set of genes with the same promoter state pattern and the genes of an expression pattern is computed using hypergeometric testing (*stats* package from R). Figure S3A similarly shows the correlation between chromatin states of distal DHSs and expression patterns. A merged set of distal DHSs were taken and clustered based on their predominant chromatin state (see "Promoter state clustering" section). The statistical significance of the overlap between a set of distal regions with the same chromatin state pattern, and the expression pattern is computed similar to Figure S2D.

**Correlation of TF binding events, DHS patterns, Histone H3K27 acetylation and gene expression dynamics**

TF binding events with significant overlap ($p$-value < $10^{-4}$) with the loci associated with 31 the gene set expression patterns were identified using Gene Set Control Analysis (GSCA) (Joshi et al., 2013). These data were overlaid as dotplots (see above) onto an expression heatmap (Figure 3B). The H3K27ac enrichment was calculated as the mean of the total number of H3K27ac tags ($X$) that overlaps with the TF binding events. After bootstrap analyses (5000 iterations of random sampling) $z$-scores were calculated as $\frac{X-\mu}{\sigma}$ where μ is the average of the mean enrichments over all iterations and σ is the standard deviation. The same analysis was done to examine the correlation between DHS patterns and histone acetylation levels in Figure S3B.

**Transcription Factor Gene Regulatory Network**

The 16 TFs for which we have ChIP-seq data were considered as nodes and the nodes coloured according to their expression values at each cell line (Figure 4). The shape of the node signifies whether there exists a DHS within 1kb of TSS and border colour signifies whether the promoter is in the state Active, Repressed, Poised or None. A directed edge from $TF_a$ to $TF_b$ indicates binding of a $TF_a$ to the locus of the $TF_b$ and the edge is

prominently displayed if $TF_a$ binds to the locus at that stage. The edge is classified and colour coded according to the promoter state of $TF_b$.

**Annotation data**

Genomic definitions for genes annotated as coding genes by RefSeq (Pruitt et al 2014), were obtained using the table browser (Karolchik et al., 2004) from the UCSC genome browser (Kent et al., 2002). The gene names for transcription factors in mouse were obtained from AnimalTFDB (Zhang et al., 2012). Genomic segments that are CG rich were obtained from the Table Browser utility of the UCSC genome browser. The percentage of the DHS_111111 regions that contain one or more CpG island is given in Figure S4B.

**Software**

R (http://www.r-project.org/) was used extensively for statistical analyses and unless otherwise stated *p*-values were Benjamini Hochberg corrected. The heatmaps in Figure 6B and Figure S6A were generated using MeV (http://www.tm4.org/mev.html). All the peaks in this study were mapped to the TSS of nearest genes (RefSeq definitions) using ChIPpeakAnno (Zhu et al., 2010) package from Rusing default parameters. BedTools and GenomicRanges were used for relevant operations involving peaks. Over-represented Gene Ontology (GO) terms were identified using DAVID (Huang da et *al*., 2009a, b) with GO_FAT_BP ontology and the default set of all mouse genes as the standard background set. Over-represented KEGG pathways for gene sets were obtained using DAVID similar to GO term enrichment analysis. Genes that belong to various families as classified by MSigDB (Liberzon et al., 2011), are preferentially highlighted in the GO tables given in Table S2, Table S3 and Table S4. The networks in Figure 4, Figure S5 and Figure S4A were generated using Cytoscape.

## Supplementary References

Abedin MJ, Nguyen A, Jiang N, Perry CE, Shelton JM, Watson DK, Ferdous A. (2014). Fli1 acts downstream of Etv2 to govern cell survival and vascular homeostasis via positive autoregulation. Circ Res *114,* 1690-1699

Anders, S., and Huber, W. (2010). Differential expression analysis for sequence count data. Genome biology *11*, R106.

Anders, S., McCarthy, D.J., Chen, Y., Okoniewski, M., Smyth, G.K., Huber, W., and Robinson, M.D. (2013). Count-based differential expression analysis of RNA sequencing data using R and Bioconductor. Nature protocols *8*, 1765-1786.

Anders, S., Pyl, P.T., and Huber, W. (2015). HTSeq-a Python framework to work with high-throughput sequencing data. Bioinformatics *31*, 166-169.

Arqués O, Chicote, I., Tenbaum, S., Puig, I., and Palmer, G., H. (2012) Standardized relative quantification of immunofluorescence tissue staining. Protocol Exchange doi:101038/protex 2012008.

Ashburner, M., Ball, C.A., Blake, J.A., Botstein, D., Butler, H., Cherry, J.M., Davis, A.P., Dolinski, K., Dwight, S.S., Eppig, J.T., *et al.* (2000). Gene ontology: tool for the unification of biology. The Gene Ontology Consortium. Nature Genetics *25*, 25-29.

Behrens AN, Zierold C, Shi X, Ren Y, Koyano-Nakagawa N, Garry DJ, Martin CM.(2012). Sox7 is regulated by ETV2 during cardiovascular development. Stem Cells Dev. *23*,2004-2014.

Brodowska K, Al-Moujahed A, Marmalidou A, Meyer Zu Horste M, Cichy J, Miller, JW, Gragoudas E, Vavvas DG (2014). The clinically used photosensitizer Verteporfin (VP) inhibits YAP-TEAD and human retinoblastoma cell growth in vitro without light activation. Exp Eye Res.*124*:67

Cline, M.S., Smoot, M., Cerami, E., Kuchinsky, A., Landys, N., Workman, C., Christmas, R., Avila-Campilo, I., Creech, M., Gross, B., *et al.* (2007). Integration of biological networks and gene expression data using Cytoscape. Nature protocols *2*, 2366-2382.

Ernst, J., and Kellis, M. (2012). ChromHMM: automating chromatin-state discovery and characterization. Nature methods *9*, 215-216.

Fehling, H.J., Lacaud, G., Kubo, A., Kennedy, M., Robertson, S., Keller, G., and Kouskoff, V. (2003). Tracking mesoderm induction and its specification to the hemangioblast during embryonic stem cell differentiation. Development *130*, 4217-4227.

Feng, J., Liu, T., Qin, B., Zhang, Y., and Liu, X.S. (2012). Identifying ChIP-seq enrichment using MACS. Nature protocols 7, 1728-1740.

Forsberg EC, Downs KM, Bresnick EH. (2000). Direct interaction of NF-E2 with hypersensitive site 2 of the beta-globin locus control region in living cells. Blood *96*, 334-339.

Heinz, S., Benner, C., Spann, N., Bertolino, E., Lin, Y.C., Laslo, P., Cheng, J.X., Murre, C., Singh, H., and Glass, C.K. (2010). Simple combinations of lineage-determining transcription factors prime cis-regulatory elements required for macrophage and B cell identities. Mol Cell *38*, 576-589.

Huang da, W., Sherman, B.T., and Lempicki, R.A. (2009a). Bioinformatics enrichment tools: paths toward the comprehensive functional analysis of large gene lists. Nucleic acids research *37*, 1-13.

Huang da, W., Sherman, B.T., and Lempicki, R.A. (2009b). Systematic and integrative analysis of large gene lists using DAVID bioinformatics resources. Nature protocols *4*, 44-57.

Iacovino, M., Chong, D., Szatmari, I., Hartweck, L., Rux, D., Caprioli, A., Cleaver, O., and Kyba, M. (2011). HoxA3 is an apical regulator of haemogenic endothelium. Nat Cell Biol *13*, 72-78.

Joshi, A., Hannah, R., Diamanti, E., and Gottgens, B. (2013). Gene set control analysis predicts hematopoietic control mechanisms from genome-wide transcription factor binding data. Experimental hematology *41*, 354-366 e314.

Karolchik, D., Hinrichs, A.S., Furey, T.S., Roskin, K.M., Sugnet, C.W., Haussler, D., and Kent, W.J. (2004). The UCSC Table Browser data retrieval tool. Nucleic acids research *32*, D493-496.

Kent, W.J., Sugnet, C.W., Furey, T.S., Roskin, K.M., Pringle, T.H., Zahler, A.M., and Haussler, D. (2002). The human genome browser at UCSC. Genome research *12*, 996-1006.

Koyano-Nakagawa N, Kweon J, Iacovino M, Shi X, Rasmussen TL, Borges L, Zirbes KM, Li T, Perlingeiro RC, Kyba M, Garry DJ. (2012). Etv2 is expressed in the yolk sac hematopoietic and endothelial progenitors and regulates Lmo2 gene expression. Stem Cells. 30, 1611-23.

Kumar, V., Muratani, M., Rayan, N.A., Kraus, P., Lufkin, T., Ng, H.H., and Prabhakar, S. (2013). Uniform, optimal signal processing of mapped deep-sequencing data. Nature biotechnology *31*, 615-622.

Lance, G. N., and Williams, W. T. (1967). A General Theory of Classificatory Sorting Strategies

1. Hierarchical Systems. The Computer Journal *9*, 373-380.

Langmead, B., Trapnell, C., Pop, M., and Salzberg, S.L. (2009). Ultrafast and memory-efficient alignment of short DNA sequences to the human genome. Genome biology *10*, R25.

Liberzon, A., Subramanian, A., Pinchback, R., Thorvaldsdottir, H., Tamayo, P., and Mesirov, J.P. (2011). Molecular signatures database (MSigDB) 3.0. Bioinformatics *27*, 1739-1740.

Lichtinger, M., Ingram, R., Hannah, R., Muller, D., Clarke, D., Assi, S.A., Lie, A.L.M., Noailles, L., Vijayabaskar, M.S., Wu, M., *et al.* (2012). RUNX1 reshapes the epigenetic landscape at the onset of haematopoiesis. Embo J *31*, 4318-4333.

Liu-Chittenden Y, Huang B, Shim JS, Chen Q, Lee SJ, Anders RA, Liu JO, Pan D. (2012) Genetic and pharmacological disruption of the TEAD-YAP complex suppresses the oncogenic activity of YAP. Genes Dev.*26*, 1300-5.

Olive, M., Williams, S.C., Dezan, C., Johnson, P.F., and Vinson, C. (1996). Design of a C/EBP-specific, dominant-negative bZIP protein with both inhibitory and gain-of-function properties. J Biol Chem *271*, 2040-2047.

Pruitt KD, Brown GR, Hiatt SM, Thibaud-Nissen F, Astashyn A, Ermolaeva O, Farrell CM, Hart J, Landrum MJ, McGarvey KM, Murphy MR, O'Leary NA, Pujar S, Rajput B, Rangwala SH, Riddick LD, Shkeda A, Sun H, Tamez P, Tully RE, Wallin C, Webb D, Weber J, Wu W, DiCuccio M, Kitts P, Maglott DR, Murphy TD, Ostell JM. (2014). RefSeq: an update on mammalian reference sequences. Nucleic Acids Res *42*, D756-63

Ptasinska A, Assi SA, Mannari D, James SR, Williamson D, Dunne J, Hoogenkamp M, Wu M, Care M, McNeill H, Cauchy P, Cullen M, Tooze RM, Tenen DG, Young BD, Cockerill PN, Westhead DR, Heidenreich O, Bonifer C. (2012) Depletion of RUNX1/ETO in t(8;21) AML cells leads to genome-wide changes in chromatin structure and transcription factor binding. Leukemia *26*):1829-41

Quinlan, A.R., and Hall, I.M. (2010). BEDTools: a flexible suite of utilities for comparing genomic features. Bioinformatics *26*, 841-842.

Rumble, S.M., Lacroute, P., Dalca, A.V., Fiume, M., Sidow, A., and Brudno, M. (2009). SHRiMP: accurate mapping of short color-space reads. PLoS computational biology *5*, e1000386.

Saeed, A.I., Sharov, V., White, J., Li, J., Liang, W., Bhagabati, N., Braisted, J., Klapa, M., Currier, T., Thiagarajan, M., et al. (2003). TM4: a free, open-source system for microarray data management and analysis. BioTechniques 34, 374-378.

Schwarz, G. (1978). Estimating the Dimension of a Model. 461-464.

Sroczynska, P., Lancrin, C., Pearson, S., Kouskoff, V., and Lacaud, G. (2009). In vitro differentiation of mouse embryonic stem cells as a model of early hematopoietic development. Methods Mol Biol *538*, 317-334.

Trapnell, C., Roberts, A., Goff, L., Pertea, G., Kim, D., Kelley, D.R., Pimentel, H., Salzberg, S.L., Rinn, J.L., and Pachter, L. (2012). Differential gene and transcript expression analysis of RNA-seq experiments with TopHat and Cufflinks. Nature protocols *7*, 562-578.

Wilson, N.K., Miranda-Saavedra, D., Kinston, S., Bonadies, N., Foster, S.D., Calero-Nieto, F., Dawson, M.A., Donaldson, I.J., Dumon, S., Frampton, J.*, et al.* (2009). The transcriptional program controlled by the stem cell leukemia gene Scl/Tal1 during early embryonic hematopoietic development. Blood *113*, 5456-5465.

Wu, H., and Ji, H. (2014). PolyaPeak: detecting transcription factor binding sites from ChIP-seq using peak shape information. PloS one *9*, e89694.

Zhang, Y., Liu, T., Meyer, C.A., Eeckhoute, J., Johnson, D.S., Bernstein, B.E., Nusbaum, C., Myers, R.M., Brown, M., Li, W., et al. (2008). Model-based analysis of ChIP-Seq (MACS). Genome biology 9, R137.

Zhang, H.M., Chen, H., Liu, W., Liu, H., Gong, J., Wang, H., and Guo, A.Y. (2012). AnimalTFDB: a comprehensive animal transcription factor database. Nucleic acids research *40*, D144-149.

Zhu, L.J., Gazin, C., Lawson, N.D., Pages, H., Lin, S.M., Lapointe, D.S., and Green, M.R. (2010). ChIPpeakAnno: a Bioconductor package to annotate ChIP-seq and ChIP-chip data. BMC bioinformatics *11*, 237.

Zwart W, Koornstra R, Wesseling J, Rutgers E, Linn S, Carroll JS.(2013). A carrier-assisted ChIP-seq method for estrogen receptor-chromatin interactions from breast cancer core needle biopsy samples. BMC Genomics *14,* 232
